# iGPCR-Drug: A Web Server for Predicting Interaction between GPCRs and Drugs in Cellular Networking

**DOI:** 10.1371/journal.pone.0072234

**Published:** 2013-08-27

**Authors:** Xuan Xiao, Jian-Liang Min, Pu Wang, Kuo-Chen Chou

**Affiliations:** 1 Computer Department, Jing-De-Zhen Ceramic Institute, Jing-De-Zhen, China; 2 Information School, ZheJiang Textile and Fashion College, NingBo, China; 3 Center of Excellence in Genomic Medicine Research (CEGMR), King Abdulaziz University, Jeddah, Saudi Arabia; 4 Gordon Life Science Institute, Belmont, Massachusetts, United States of America; University of South Alabama Mitchell Cancer Institute, United States of America

## Abstract

Involved in many diseases such as cancer, diabetes, neurodegenerative, inflammatory and respiratory disorders, G-protein-coupled receptors (GPCRs) are among the most frequent targets of therapeutic drugs. It is time-consuming and expensive to determine whether a drug and a GPCR are to interact with each other in a cellular network purely by means of experimental techniques. Although some computational methods were developed in this regard based on the knowledge of the 3D (dimensional) structure of protein, unfortunately their usage is quite limited because the 3D structures for most GPCRs are still unknown. To overcome the situation, a sequence-based classifier, called “**iGPCR-drug**”, was developed to predict the interactions between GPCRs and drugs in cellular networking. In the predictor, the drug compound is formulated by a 2D (dimensional) fingerprint via a 256D vector, GPCR by the PseAAC (pseudo amino acid composition) generated with the grey model theory, and the prediction engine is operated by the fuzzy K-nearest neighbour algorithm. Moreover, a user-friendly web-server for **iGPCR-drug** was established at http://www.jci-bioinfo.cn/iGPCR-Drug/. For the convenience of most experimental scientists, a step-by-step guide is provided on how to use the web-server to get the desired results without the need to follow the complicated math equations presented in this paper just for its integrity. The overall success rate achieved by **iGPCR-drug** via the jackknife test was 85.5%, which is remarkably higher than the rate by the existing peer method developed in 2010 although no web server was ever established for it. It is anticipated that **iGPCR-Drug** may become a useful high throughput tool for both basic research and drug development, and that the approach presented here can also be extended to study other drug – target interaction networks.

## Introduction

**Figure 1:**
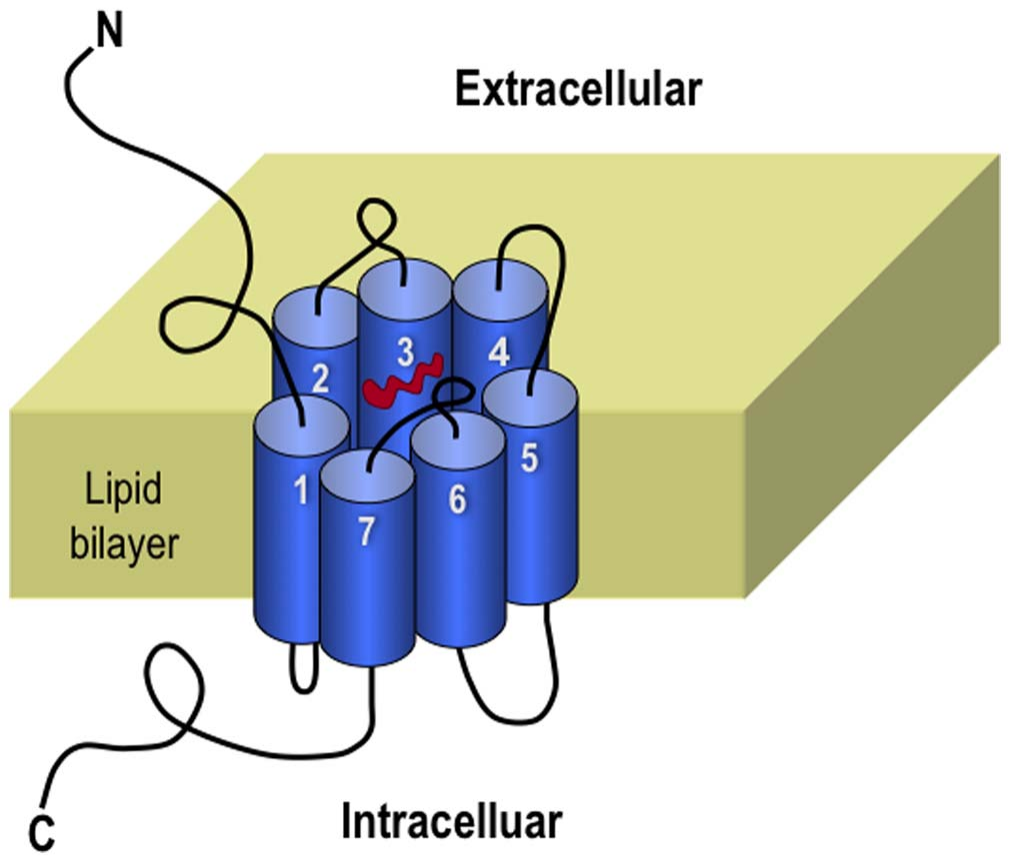
Schematic drawing of a GPCR. It consists of seven transmembrane alpha helices, intracellular C-terminal, an extracellular N-terminal, three intracellular loops and three extracellular loops. Reproduced from [4] with permission.

G-protein-coupled receptors (GPCRs), also known as G protein-linked receptors (GPLR), serpentine receptor, seven-transmembrane domain receptors, and 7 TM (transmembrane), form the largest family of cell surface receptors. GPCRs share a common global topology that consists of seven transmembrane alpha helices, intracellular C-terminal, an extracellular N-terminal, three intracellular loops and three extracellular loops (**Fig. 1**).

GPCR-associated proteins may play at least the following four distinct roles in receptor signaling: **(1)** directly mediate receptor signaling, as in the case of G proteins; **(2)** regulate receptor signaling through controlling receptor localization and/or trafficking; **(3)** act as a scaffold, physically linking the receptor to various effectors; **(4)** act as an allosteric modulator of receptor conformation, altering receptor pharmacology and/or other aspects of receptor function [1], [2], [3]. Involved in many diseases such as cancer, diabetes, neurodegenerative, inflammatory and respiratory disorders, GPCRs are among the most frequent targets of therapeutic drugs [4]. Over half of all prescription drugs currently on the market are actually acting by targeting GPCRs directly or indirectly [5], [6]. A lot of efforts have been invested for studying GPCRs in both academic institutions and pharmaceutical industries.

Identification of drug-target interactions is an essential step in the drug discovery process, which is the most important task for the new medicine development [7]. The methods commonly used in this regard are docking simulations [8], [9], literature text mining [10], as well as a combination of chemical structure, genomic sequence, and 3D (three-dimensional) structure information, among others [11]. Obviously, an experimental 3D structure of a target protein is the key for identifying the drug-protein interaction; if it is not available, the common approach is to create a homology model of the target protein based on the experimental structure of a related protein [12], [13], [14]. However, the above methods need further development due to the following reasons. (1) None of these methods has provided a web-server for the public usage, and hence their practical application value is quite limited. (2) The prediction quality needs to be improved with the state-of-the-art machine learning techniques and updated training datasets. (3) GPCRs belong to membrane proteins, which are very difficult to crystallize and most of them will not dissolve in normal solvents. Although a series of recent reports [15], [16], [17], [18], [19], [20], [21] have demonstrated that NMR is indeed a very powerful tool in determining the 3D structures of membrane proteins, it is time-consuming and costly. Also, although using various structural bioinformatics tools [12], particularly the homologous technique [22], [23], [24], [25], [26], can help acquire the structural and functional information of membrane proteins, unfortunately the number of templates for membrane proteins is quite limited. Therefore, it would be very useful to develop a computational method for predicting the interactions between drugs and GPCRs in cellular networking based on their sequences-derived features before a drug candidate was synthesized, so as to reduce the unnecessary waste of time and money [27]. And this is the goal of the current study.

According to a recent comprehensive review [28] and demonstrated by a series of recent publications (see, e.g., [29], [30], [31]), to establish a really useful statistical predictor for a protein system, we need to consider the following procedures: (i) construct or select a valid benchmark dataset to train and test the predictor; (ii) formulate the protein samples with an effective mathematical expression that can truly reflect their intrinsic correlation with the attribute to be predicted; (iii) introduce or develop a powerful algorithm (or engine) to operate the prediction; (iv) properly perform cross-validation tests to objectively evaluate the anticipated accuracy of the predictor; (v) establish a user-friendly web-server for the predictor that is accessible to the public. Below, let us describe how to deal with these steps.

## Methodology

### 1. Benchmark Dataset

The benchmark dataset 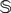 can be formulated as
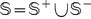
(1)where 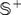 is the positive subset that consists of the interactive GPCR-drug pairs only, while 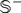 the negative subset that contains of the non-interactive GPCR-drug pairs only, and the symbol 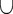 represents the union in the set theory. Here, the “interactive” pair means the pair whose two counterparts are interacted with each other in the drug-target networks as defined in the KEGG database at http://www.kegg.jp/kegg/; while the “non-interactive” pair means that its two counterparts are not interacted with each other in the drug-target networks. The positive dataset 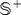 contains 620 GPCR-drug pairs, which were taken from [32]. The negative dataset 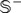 contains 1,240 non-interactive GPCR-drug pairs, which were derived according to the following procedures as done in [32]: (i) separating each of the pairs in 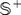 into single drug and GPCR; (ii) re-coupling each of the single drugs with each of the single GPCRs into pairs in a way that none of them occurred in 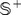; (iii) randomly picking the pairs thus formed until they reached the number two times as many as the pairs in 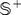. The 620 interactive GPCR-drug pairs and 1,240 non-interactive GPCR-drug pairs are given in Supporting Information S1. All the detailed information for the compounds or drugs listed there can be found in the KEGG database via their codes.

### 2. Sample Representation

Since each of the samples in the current network system contains a GPCR (protein) and a drug, a combination of the following two approaches were adopted to represent the GPCR
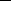
drug pair samples.

#### (a) Representing drugs with 2D molecular fingerprints

Although the number of drugs is extremely large, most of them are small organic molecules and are composed of some fixed small structures [33]. The identification of small molecules or structures can be used to detect the drug-target interactions [34]. Molecular fingerprints are bit-string representations of molecular structure and properties [35]. It should be pointed out that there are many types of structural representation that have been suggested for the description of drug molecules, including physicochemical properties [36], chemical graphs [37], topological indices [38], 3D pharmacophore patterns and molecular fields. In the current study, let us use the simple and generally adopted 2D molecular fingerprints to represent drug molecules, as described below.

First, for each of the drugs concerned, we can obtain a MOL file from the KEGG database [39] via its code that contains the detailed information of chemical structure. Second, we can convert the MOL file format into its 2D molecular fingerprint file format by using a chemical toolbox software called OpenBabel [40], which can be downloaded from the website at http://openbabel.org/. The current version of OpenBabel can generate four types of fingerprints: FP2, FP3, FP4 and MACCS. In the current study, we used the FP2 fingerprint format. It is a path-based fingerprint that identifies small molecule fragments based on all linear and ring substructures and maps them onto a bit-string using a hash function (somewhat similar to the Daylight fingerprints [41], [42]). It is a length of 256-bit hexadecimal string or a 256-bit vector, whose component values are an integer between 0 and 15. Let us suppose 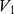 is the 1st value of the 256-bit vector, 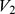 that of the 2nd value, and so forth. Thus, the 256-bit vector can be converted to a digit signal. In order to find the inwardness of the drug fingerprint values, we implement the discrete Fourier transform, with the frequency-domain values given by
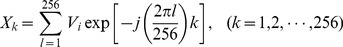
(2)where *j* represents the imaginary unit and 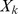 is a complex number whose complex modulus or amplitude is given by
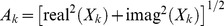
(3)where 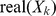 is the real part of 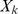 and 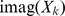 the corresponding image part. Thus we can generate the discrete Fourier spectrum as given by



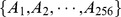
(4)The Fourier spectrum numbers contain substantial information about the digit signal, and hence can also be used to reflect certain characters of a drug. Thus, a drug compound 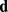 now can be formulated as a 256-D (dimensional) vector given by
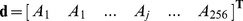
(5)where 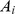 has the same meaning as in **Eq. 4**, and 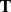 is the matrix transpose operator.

The 256-D vector thus obtained for each of the drug codes listed in Supporting Information S1 are given in Supporting Information S2.

#### (b) Representing GPCR sequences with grey model pseudo amino acid composition

The sequences of the GPCRs involved in this study are given in Supporting Information S3. Now the problem is how to effectively represent these receptor sequences for the current study. Generally speaking, there are two kinds of approaches to formulate protein sequences: the sequential model and the non-sequential or discrete model [43]. The most typical sequential representation for a protein sample with 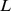 residues is its entire amino acid sequence, as can be formulated as
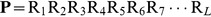
(6)where 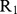 represents the 1^st^ residue of the protein sequence 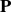, 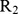 the 2^nd^ residue, and so forth. A protein thus formulated can contain its most complete information. This is an obvious advantage of the sequential representation. To get the desired results, the sequence-similarity-search-based tools, such as BLAST [44], [45], are usually utilized to conduct the prediction. However, this kind of approach failed to work when the query protein did not have significant homology to proteins of known characters. Thus, various non-sequential representation models were proposed. The simplest non-sequential model for a protein was based on its amino acid composition (AAC), as defined by
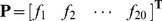
(7)where 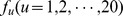 are the normalized occurrence frequencies of the 20 native amino acids [46], [47] in the protein 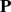, and 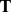 has the same meaning as in Eq. 5. The AAC-discrete model was widely used for identifying various attributes of proteins. However, as can be seen from Eq. 7, all the sequence order effects were lost by using the AAC-discrete model. This is its main shortcoming. To avoid completely losing the sequence-order information, the pseudo amino acid composition was proposed [48] to replace the simple amino acid composition (AAC) for representing the sample of a protein. Since the concept of PseAAC (also called “Chou’s PseAAC” [49]) was proposed in 2001 [48], it has been widely used to study various attributes of proteins, such as discriminating outer membrane proteins [50], identifying antibacterial peptides [51], identifying allergenic proteins [52], predicting metalloproteinase family [53], predicting protein structural class [54], identifying bacterial virulent proteins [55], predicting supersecondary structure [56], predicting protein subcellular location [57], [58], [59], [60], predicting membrane protein types [61], [62], identifying GPCRs and their types [63], identifying protein quaternary structural attributes [64], predicting protein submitochondria locations [65], identifying risk type of human papillomaviruses [66], identifying cyclin proteins [67], predicting GABA(A) receptor proteins [68], classifying amino acids [69], predicting cysteine S-nitrosylation sites in proteins [30], among many others (see a long list of papers cited in the References section of [28]). Recently, the concept of PseAAC was further extended to represent the feature vectors of DNA and nucleotides [29], [31], as well as other biological samples (see, e.g., [70], [71]). Because it has been widely and increasingly used, recently two powerful soft-wares called “PseAAC-Builder” [72] and “propy” [73] were established for generating various special Chou’s pseudo-amino acid compositions, in addition to the web-server PseAAC [74] built in 2008. According to a recent review [28], the general form of PseAAC for a protein 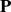 is formulated by
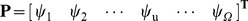
(8)where the subscript 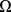 is an integer, and its value as well as the components 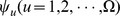 will depend on how to extract the desired information from the amino acid sequence of 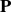 (cf. **Eq. 6**). Below, let us describe how to extract useful information from the benchmark dataset 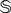 and Supporting Information S2 to define the GPCR samples concerned via Eq. 8.

First, let us represent the protein sequence by a series of real numbers. Listed in **Table 1** are the ten different kinds of physicochemical properties usually used for identifying protein attributes [75]. For the current study, however, it was found through many preliminary tests that when the 10^th^ physicochemical property (i.e., the “mean polarity”) was used, the best prediction quality was observed. This is quite consistent with the observations that polar amino acids play an important role in membrane protein receptors [12], [17]. Accordingly, the 20 numerical values of the mean polarity in **Table 1** were used to encode the 20 native amino acids in a GPCR sequence. Note that to ensure that each of these numerical codes was a positive number as required by the Grey model used later, during the encoding process, each of the mean polarity values in **Table 1** was added by a number of 1.20. Thus, for a given GPCR sequence with 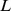 amino acids (cf. **Eq. 6**), we can convert it into a series of 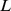 real number as formulated by
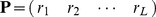
(9)where 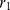 is the mean polarity value for the 1^st^ amino acid residue in the GPCR protein 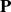, e.g., if the 1^st^ residue is A, then we have 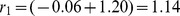; 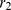 is the mean polarity value for the 2^nd^ amino acid residue plus 1.20; and so forth. Now, we can use the grey system model to extract the useful information of 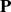 via **Eq. 8** to formulate its PseAAC.

**Table 1:** Ten physicochemical property codes for each of the 20 native amino acids^a^.

Amino acid	Ten physicochemical property codes^b^									
	1st	2nd	3rd	4th	5th	6th	7th	8th	9th	10th
A	0.62	−0.50	15	2.35	9.87	6.11	91.50	89.09	27.5	−0.06
C	0.29	−1.00	47	1.71	10.78	5.02	117.7	121.2	44.6	1.36
D	−0.90	3.00	59	1.88	9.60	2.98	124.5	133.1	40.0	−0.80
E	−0.74	3.00	73	2.19	9.67	3.08	155.1	147.1	62.0	−0.77
F	1.19	22.50	91	2.58	9.24	5.91	203.4	165.2	115.5	1.27
G	0.48	0.00	1	2.34	9.60	6.06	66.40	75.07	0.0	−0.41
H	−0.40	20.50	82	1.78	8.97	7.64	167.3	155.2	79.0	0.49
I	1.38	21.80	57	2.32	9.76	6.04	168.8	131.2	93.5	1.31
K	−1.50	3.00	73	2.20	8.90	9.47	171.3	146.2	100.0	−1.18
L	1.06	21.80	57	2.36	9.60	6.04	167.9	131.2	93.5	1.21
M	0.64	21.30	75	2.28	9.21	5.74	170.8	149.2	94.1	1.27
N	−0.78	0.20	58	2.18	9.09	10.76	135.2	132.1	58.7	−0.48
P	0.12	0.00	42	1.99	10.60	6.30	129.3	115.1	41.9	0.00
Q	−0.85	0.20	72	2.17	9.13	5.65	161.1	146.2	80.7	−0.73
R	−2.53	3.00	101	2.18	9.09	10.76	202.0	174.2	105	−0.84
S	−0.18	0.30	31	2.21	9.15	5.68	99.10	105.1	29.3	−0.50
T	−0.05	20.40	45	2.15	9.12	5.60	122.1	119.1	51.3	−0.27
V	1.08	21.50	43	2.29	9.74	6.02	141.7	117.2	71.5	1.09
W	0.81	23.40	130	2.38	9.39	5.88	237.6	204.2	145.5	0.88
Y	0.26	22.30	107	2.20	9.11	5.63	203.6	181.2	117.3	0.33

a The numerical codes of the physicochemical properties can be obtained from the text biochemistry book (e.g., [101]) and the papers [102], [103].

b The 1^st^ physicochemical property is for “hydrophobicity”, 2^nd^ for “hydrophilicity”, 3^rd^ for “side-chain mass”, 4^th^ for “pK1 (C^a^-COOH)”, 5^th^ for “pK2 (NH3)”, 6^th^ for “PI (25°C)”, 7^th^ for “average buried volume”, 8^th^ for “molecular weight”, 9^th^ for “side-chain volume”, and 10^th^ for “mean polarity”.

According to the grey system theory [76], if the information of a system investigated is fully known, it is called a “white system”; if completely unknown, a “black system”; if partially known, a “grey system”. The model developed based on such a theory is called “grey model”, which is a kind of nonlinear and dynamic model formulated by a differential equation. The grey model is particularly useful for solving complicated problems [77] that are lack of sufficient information, or need to process uncertain information and reduce random effects of acquired data. In the grey system theory, an important and generally used model is called GM(1,1) [76]. By following the similar procedures as described in [78], [79], [80], [81], **Eq. 8** would become a feature vector with dimension 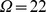 and each of its components defined by
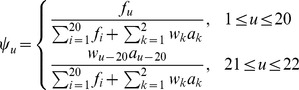
(10)where 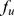 has the same meaning as **Eq. 7**, 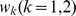 is the weight factor (in this study we choose 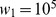 and 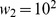 to get the best results), and 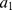 and 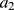 are given by 
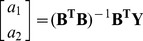
(11) where 
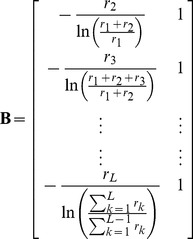
(12) and 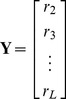
(13)


#### (c) Representing GPCR-drug pairs

Now the pair between a GPCR protein 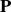 and a drug compound **D** can be formulated by combing **Eq. 5** and **Eq. 8**, as given by
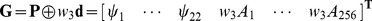
(14)where **G** represents the GPCR-drug pair, 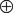 the orthogonal sum [43], 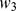 the weight factor that was chosen as 1/700 in this study to get the best results, and 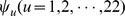 are given in **Eq. 10**.

### 3. Fuzzy *K*-Nearest Neighbor Algorithm

The fuzzy *K*-Nearest Neighbour (*K*NN) classification method [82] is quite popular in the pattern recognition community owing to its good performance and ease of use. It is particularly effective in dealing with complicated biological systems, such as identifying nuclear receptor subfamilies [83], characterizing the structure of fast-folding proteins [84], classifying G protein-coupled receptors [85], predicting protein quaternary structural attributes [86], predicting protein structural classes [87], [88], [89], [90], identifying membrane protein types [91], and so forth. The rationale of the fuzzy method is based on the fact that it is impossible to define a feature vector that can contain all the entire information of a complicated system. Therefore, it is logically more reasonable to treat this kind of object as a fuzzy system. Below, let us give a brief introduction how to use the fuzzy *K*NN approach to identify the interactions between the GPCR and the drug compounds in the network concerned.

Suppose 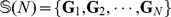 is a set of vectors representing 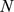 GPCR-drug pairs in a training set classified into two classes 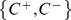, where 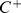 denotes the interactive pair class while 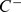 the non-interactive pair class; 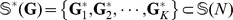 is the subset of the *K* nearest neighbor pairs to the query pair 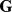. Thus, the fuzzy membership value for the query pair 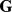 in the two classes of 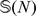 is given by [92]

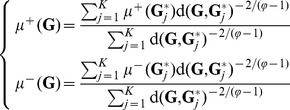
(15)where *K* is the number of the nearest neighbors counted for the query pair 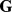; 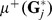 and 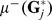, the fuzzy membership values of the training sample 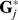 to the class 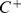 and 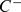, respectively, as will be further defined below; 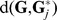 **,** the Euclidean distance between 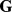 and its *j*th nearest pair 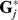 in the training dataset 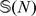; 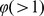, the fuzzy coefficient for determining how heavily the distance is weighted when calculating each nearest neighbor’s contribution to the membership value. Note that the parameters *K* and 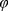 will affect the computation result of **Eq. 15**, and they will be optimized by a grid-search as will be described later. Also, various other metrics can be chosen for 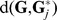, such as Hamming distance [93] and Mahalanobis distance [94], [95].

The quantitative definitions for the aforementioned 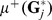 and 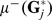 in **Eq. 15** are given by
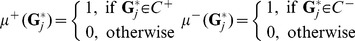
(16)


Substituting the results obtained by **Eq. 16** into **Eq. 15**, it follows that if 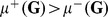 then the query pair 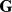 is an interactive couple; otherwise, non-interactive. In other words, the outcome can be formulated as
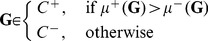
(17)


**Figure 2:**
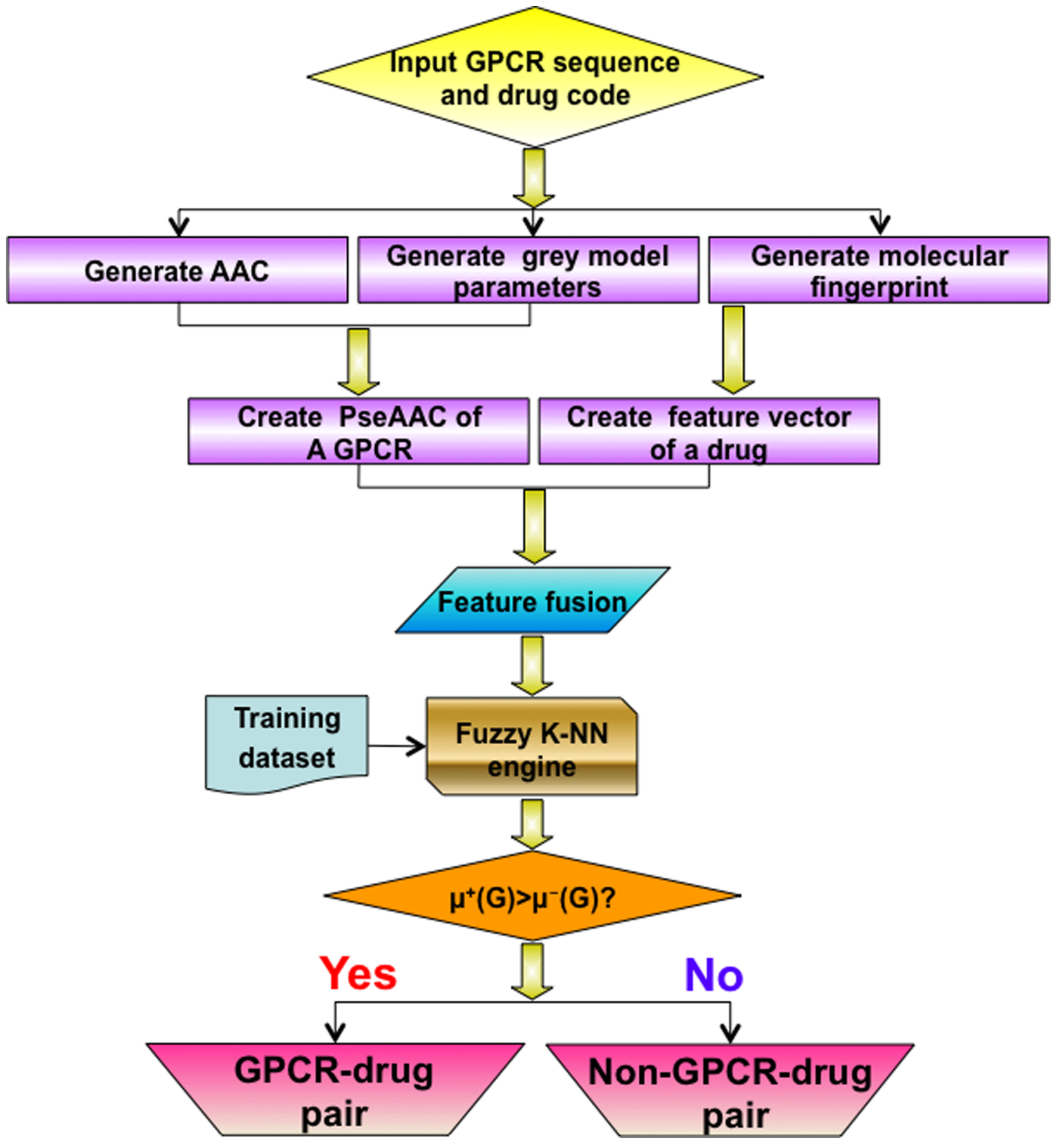
A flowchart to show the operation process of the iGPCR-Drug predictor. See the text for further explanation.

The predictor thus established is called **iGPCR-Drug**. To provide an intuitive overall picture of how the classifier works, a flowchart is provided in **Fig. 2** to show its operation process.

## Results and Discussion

### 1. Metrics for Performance Evaluation

To provide a more intuitive and easier-to-understand method to measure the prediction quality, here the criteria proposed in [96] was adopted. According to those criteria, the rates of correct predictions for the interactive GPCR-drug pairs in dataset 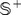 and the non-interactive GPCR-drug pairs in dataset 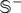 are respectively defined by (cf. **Eq. 1**).
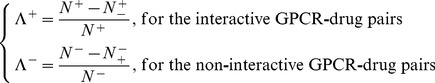
(18)where 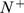 is the total number of the interactive GPCR-drug pairs investigated while 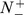 the number of the interactive GPCR-drug pairs incorrectly predicted as the non-interactive GPCR-drug pairs; 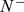 the total number of the non-interactive GPCR-drug pairs investigated while 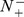 the number of the non-interactive GPCR-drug pairs incorrectly predicted as the interactive GPCR-drug pairs. The overall success prediction rate is given by [97]



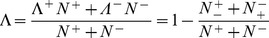
(19)It is obvious from **Eqs. 18–19** that, if and only if none of the interactive GPCR-drug pairs and the non-interactive GPCR-drug pairs are mispredicted, i.e., 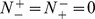 and 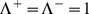, we have the overall success rate 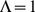. Otherwise, the overall success rate would be smaller than 1.

On the other hand, it is interesting to point out that the following equation set is often used in literatures for examining the performance quality of a predictor.
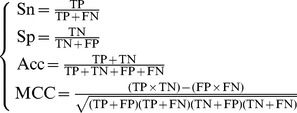
(20)where TP represents the true positive; TN, the true negative; FP, the false positive; FN, the false negative; Sn, the sensitivity; Sp, the specificity; Acc, the accuracy; MCC, the Mathew’s correlation coefficient.

Obviously, the relations between the symbols in **Eq. 18** or **Eq. 19** and those in **Eq. 20** are given by
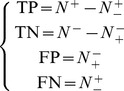
(21)


Substituting **Eq. 21** into **Eq. 20** and also noting **Eqs. 18–19,** we obtain.
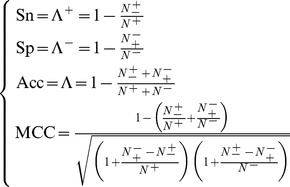
(22)


Now we can easily see: when 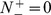 meaning none of the interactive GPCR-drug pairs was mispredicted to be a non-interactive GPCR-drug pair, we have the sensitivity 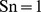; while 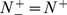 meaning that all the interactive GPCR-drug pairs were mispredicted to be the non-interactive GPCR-drug pairs, we have the sensitivity 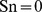. Likewise, when 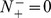 meaning none of the non-interactive GPCR-drug pairs was mispredicted, we have the specificity 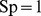; while 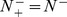 meaning all the non-interactive GPCR-drug pairs were incorrectly predicted as interactive GPCR-drug pairs, we have the specificity 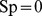. When 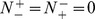 meaning that none of the interactive GPCR-drug pairs in the dataset 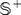 and none of the non-interactive GPCR-drug pairs in 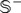 was incorrectly predicted, we have the overall accuracy 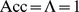; while 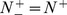 and 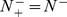 meaning that all the interactive GPCR-drug pairs in the dataset 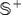 and all the non-interactive GPCR-drug pairs in 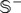 were mispredicted, we have the overall accuracy 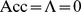. The MCC correlation coefficient is usually used for measuring the quality of binary (two-class) classifications. When 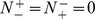 meaning that none of the interactive GPCR-drug pairs in the dataset 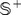 and none of the non-interactive GPCR-drug pairs in 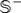 was mispredicted, we have 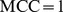; when 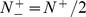 and 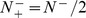 we have 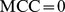 meaning no better than random prediction; when 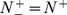 and 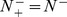
we have 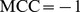 meaning total disagreement between prediction and observation. As we can see from the above discussion, it is much more intuitive and easier-to-understand when using **Eq. 22** to examine a predictor for its sensitivity, specificity, overall accuracy, and Mathew’s correlation coefficient.

### 2. Cross-Validation

**Figure 3:**
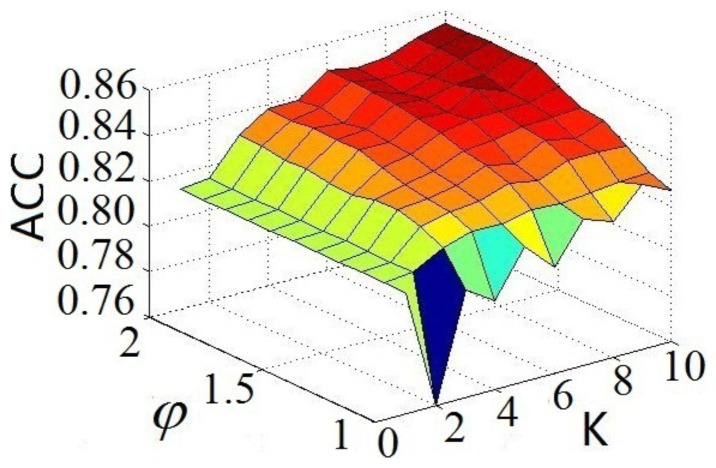
A 3D graph to show how to optimize the two parameters *K* and 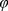 for the iGPCR-Drug predictor.

How to properly examine the prediction quality is a key for developing a new predictor and estimating its potential application value. Generally speaking, to avoid the “memory effect” [43] of the resubstitution test in which a same dataset was used to train and test a predictor, the following three cross-validation methods are often used to examine a predictor for its effectiveness in practical application: independent dataset test, subsampling or *K*-fold (such as 5-fold, 7-fold, or 10-fold) test, and jackknife test [93]. However, as elaborated by a penetrating analysis in [98], considerable arbitrariness exists in the independent dataset test. Also, as demonstrated by Eqs. 28–30 in [28], the subsampling test (or K-fold cross validation) cannot avoid arbitrariness either. Only the jackknife test is the least arbitrary that can always yield a unique result for a given benchmark dataset. Therefore, the jackknife test has been widely recognized and increasingly adopted by investigators to examine the quality of various predictors (see, e.g., [51], [52], [99]). In view of this, the success rate by the jackknife test was also used to optimize the two uncertain parameters *K* and 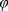 in **Eq. 15**. The result thus obtained is shown in **Fig. 3**, from which we obtain when 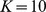 and 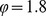 the **iGPCR-Drug** predictor reaches its optimized status.

The success rates thus obtained by the jackknife test in identifying interactive GPCR-drug pairs or non-interactive GPCR-drug pairs are given in **Table 2**, from which we can see that the overall success rate by **iGPCR-Drug** on the benchmark dataset 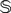 was about 85.5%. In contrast, the corresponding success rate obtained by He et al. [32] in using six biochemical and physicochemical features to formulate GPCR-Drug samples was only 78.49%. The remarkable improved success rate indicates that introducing 2D molecular fingerprints to represent drug samples and using the grey PseAAC to represent GPCR samples are really a promising approach for studying the interactions of GPCRs and drugs in cellular network, where the former can catch the essence of the drug sample whereas the latter can catch the essence of the GPCR sample.

**Table 2:** The jackknife success rates obtained iGPCR-Drug in identifying interactive GPCR-drug pairs and non-interactive GPCR-drug pairs for the benchmark dataset 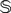 (cf. Supporting Information S1).

Performance evaluation (cf. Eq. 10 or 22)	iGPCR-Drug^a^	Method by He et al.^b^
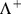 Sn or	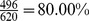	N/A
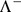 Sp or	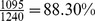	N/A
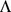 Acc or	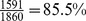	78.49%
MCC	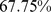	N/A

a The parameters used: 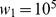 and 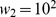 (cf. **Eq. 10), 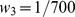
** (cf. **Eq. 14**)**,** and 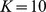 and 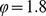 (cf. **Eq. 15**).

b See ref. [32].

It is instructive to point out that, compared with the existing sequence-based methods, although the current approach could get better results because of introducing the 2D molecular fingerprints to represent drug samples and using grey PseAAC to represent the GPCR samples, it is still a sequence-based or “sequence-derived” approach, and hence could not avoid some limitation. Particularly, it cannot be used to predict the binding site and binding energy between GPCR and drug. Only when the 3D structures for both the GPCR receptor and its drug ligand are known or well defined, can we try to predict their binding details via molecular docking (see, e.g., [9]). Nevertheless, before their 3D structures are available, the current sequence-derived approach can serve as a high throughput tool for predicting GPCR–drug interactions in cellular networking. This is particularly useful in conducting large-scale analysis for the avalanche of biological sequences generated in the post-genomic age.

Besides, to further validate the current predictor, we took 314 GPCR-drug pairs from the study by Yamanishi et al. [100] that had been confirmed by experiments as interactive pairs and none of them occurred in the current benchmark dataset used to train our predictor. It was observed that, of the 314 pairs in such an independent dataset, 271 were correctly identified by **iGPCR-Drug** as interactive pairs; i.e., the success rate was 86.33%, quite consistent with the above-mentioned jackknife success rate (85.55%) achieved by the predictor on the benchmark dataset 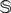 (cf. **Eq. 1**).

To enhance the value of its practical applications, the web server for **iGPCR-Drug** has been established that can be freely accessible at http://www.jci-bioinfo.cn/iGPCR-Drug/. It is anticipated that the web server will become a useful high throughput tool for both basic research and drug development in the relevant areas, or at the very least play a complementary role to the existing method [32] for which so far no web-server whatsoever has been provided yet.

### 3. The Protocol or User Guide

For the convenience of the vast majority of biologists and pharmaceutical scientists, here let us provide a step-by-step guide to show how the users can easily get the desired result by means of the web server without the need to follow the complicated mathematical equations presented in this paper for the process of developing the predictor and its integrity.

#### Step 1

**Figure 4:**
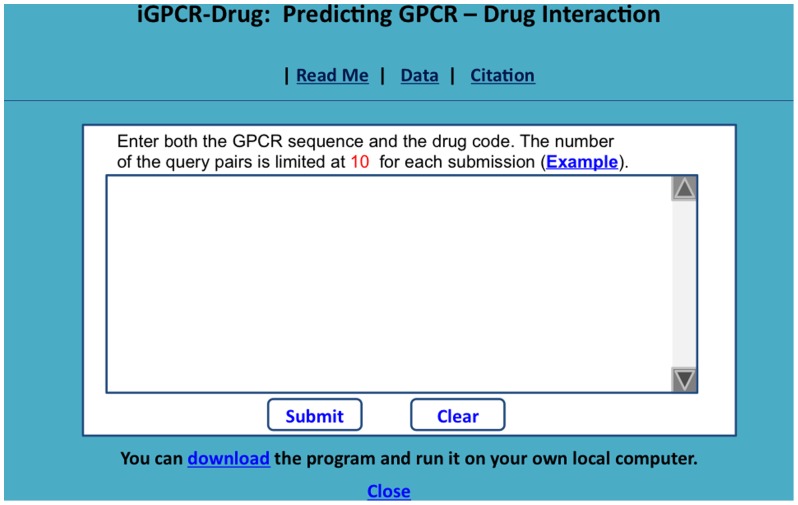
A semi-screenshot to show the top page of the iGPCR-Drug web-server. Its web-site address is at http://www.jci-bioinfo.cn/iGPCR-Drug.

Open the web server at the site http://www.jci-bioinfo.cn/iGPCR-Drug/and you will see the top page of the predictor on your computer screen, as shown in **Fig. 4**. Click on the Read Me button to see a brief introduction about **iGPCR-Drug** predictor and the caveat when using it.

#### Step 2

Either type or copy/paste the query pairs into the input box at the center of **Fig. 4**. Each query pair consists of two parts: one is for the protein sequence, and the other for the drug. The GPCR sequence should be in FASTA format, while the drug in the KEGG code. Examples for the query pairs input can be seen by clicking on the Example button right above the input box.

#### Step 3

Click on the Submit button to see the predicted result. For example, if you use the four query pairs in the Example window as the input, after clicking the Submit button, you will see on your screen that the “hsa:10161” GPCR and the “D00528” drug are an interactive pair, and that the “hsa:10800” GPCR and the “D00411” drug are also an interactive pair, but that the “hsa:1909” GPCR and the “D02566” drug are not an interactive pair, and that the “hsa:2913” GPCR and the “D01699” drug are not an interactive pair either. All these results are fully consistent with the experimental observations. It takes about 10 seconds before the results are shown on the screen.

#### Step 4

Click on the Citation button to find the relevant paper that documents the detailed development and algorithm of **iGPCR-Durg**.

#### Step 5

Click on the Data button to download the benchmark dataset used to train and test the **iGPCR-Durg** predictor.

#### Step 6

The program code is also available by clicking the button download on the lower panel of **Fig. 4**.

## Supporting Information

Supporting Information S1 The benchmark dataset contains 1,860 GPCR-drug pair samples, of which 620 are interactive and 1,240 non-interactive. The codes listed here were from the KEGG database at http://www.kegg.jp/kegg/.
(PDF)

Supporting Information S2 The fingerprints for the drug codes listed in Supporting Information S1. Each of these fingerprints is a 256-D vectors generated by the OpenBabel software downloaded from http://openbabel.org/.
(PDF)

Supporting Information S3 The protein sequences for the GPCRs listed in Supporting Information S1.
(PDF)
