# Supplementary material for: iGPCR-Drug: A Web Server for Predicting Interaction between GPCRs and Drugs in Cellular Networking
Source: PLoS One. 2013 Aug 27;8(8):e72234. doi: 10.1371/journal.pone.0072234 (PMC3754978; doi:10.1371/journal.pone.0072234)
Supplement: Supporting Information S1 — The benchmark dataset contains 1,860 GPCR-drug pair samples, of which 620 are interactive and 1,240 non-interactive. The codes listed here were from the KEGG database at http://www.kegg.jp/kegg/. (PDF) [file pone.0072234.s001.pdf]

Online Supporting Information S1. The benchmark dataset $\mathbb{S} = \mathbb{S}^{+} \cup \mathbb{S}^{-}$ contains 1,860 GPCR-drug pair samples, of which 620 are interactive ($\mathbb{S}^{+}$) and 1,240 non-interactive ($\mathbb{S}^{-}$). The codes listed here were from the KEGG database at http://www.kegg.jp/kegg/.

Pair attribute	Target GPCRs gene code in KEGG	Drug code
Interactive	hsa:10161	D00528
Interactive	hsa:10800	D00411
Interactive	hsa:10800	D01828
Interactive	hsa:11255	D00234
Interactive	hsa:11255	D00300
Interactive	hsa:11255	D00318
Interactive	hsa:11255	D00494
Interactive	hsa:11255	D02566
Interactive	hsa:1128	D00113
Interactive	hsa:1128	D00232
Interactive	hsa:1128	D00274
Interactive	hsa:1128	D00283
Interactive	hsa:1128	D00397
Interactive	hsa:1128	D00454
Interactive	hsa:1128	D00465
Interactive	hsa:1128	D00494
Interactive	hsa:1128	D00524
Interactive	hsa:1128	D00525
Interactive	hsa:1128	D00540
Interactive	hsa:1128	D00646
Interactive	hsa:1128	D00715
Interactive	hsa:1128	D00726
Interactive	hsa:1128	D00779
Interactive	hsa:1128	D01103
Interactive	hsa:1128	D01118
Interactive	hsa:1128	D01269
Interactive	hsa:1128	D01297
Interactive	hsa:1128	D01871
Interactive	hsa:1128	D02070
Interactive	hsa:1128	D02327
Interactive	hsa:1128	D02354
Interactive	hsa:1128	D02356
Interactive	hsa:1128	D02361
Interactive	hsa:1128	D03621
Interactive	hsa:1128	D03858
Interactive	hsa:1129	D00113
Interactive	hsa:1129	D00232
Interactive	hsa:1129	D00274
Interactive	hsa:1129	D00283

Interactive	hsa:1129	D00397
Interactive	hsa:1129	D00454
Interactive	hsa:1129	D00465
Interactive	hsa:1129	D00494
Interactive	hsa:1129	D00524
Interactive	hsa:1129	D00540
Interactive	hsa:1129	D00646
Interactive	hsa:1129	D00760
Interactive	hsa:1129	D00765
Interactive	hsa:1129	D00779
Interactive	hsa:1129	D01871
Interactive	hsa:1129	D02070
Interactive	hsa:1129	D02354
Interactive	hsa:1129	D02356
Interactive	hsa:1129	D02361
Interactive	hsa:1129	D03621
Interactive	hsa:1129	D03858
Interactive	hsa:1131	D00113
Interactive	hsa:1131	D00232
Interactive	hsa:1131	D00454
Interactive	hsa:1131	D00494
Interactive	hsa:1131	D01699
Interactive	hsa:1131	D01871
Interactive	hsa:1131	D02070
Interactive	hsa:1131	D02354
Interactive	hsa:1131	D02361
Interactive	hsa:1131	D03621
Interactive	hsa:1131	D03654
Interactive	hsa:1131	D03858
Interactive	hsa:1132	D00113
Interactive	hsa:1132	D00397
Interactive	hsa:1132	D00454
Interactive	hsa:1132	D00494
Interactive	hsa:1132	D01871
Interactive	hsa:1132	D02070
Interactive	hsa:1132	D02354
Interactive	hsa:1132	D02361
Interactive	hsa:1133	D00113
Interactive	hsa:1133	D00454
Interactive	hsa:1133	D00494
Interactive	hsa:1133	D01871
Interactive	hsa:1133	D02070
Interactive	hsa:1133	D02354
Interactive	hsa:1133	D02361
Interactive	hsa:1234	D03210
Interactive	hsa:1241	D00411
Interactive	hsa:1268	D00306

Interactive	hsa:134	D00227
Interactive	hsa:134	D00332
Interactive	hsa:134	D00528
Interactive	hsa:134	D01712
Interactive	hsa:134	D02884
Interactive	hsa:135	D00227
Interactive	hsa:135	D00528
Interactive	hsa:135	D01712
Interactive	hsa:135	D02884
Interactive	hsa:136	D00371
Interactive	hsa:136	D00528
Interactive	hsa:136	D04006
Interactive	hsa:140	D00227
Interactive	hsa:140	D00528
Interactive	hsa:146	D00255
Interactive	hsa:146	D00281
Interactive	hsa:146	D00283
Interactive	hsa:146	D00426
Interactive	hsa:146	D00437
Interactive	hsa:146	D00454
Interactive	hsa:146	D00509
Interactive	hsa:146	D00513
Interactive	hsa:146	D00604
Interactive	hsa:146	D00607
Interactive	hsa:146	D00609
Interactive	hsa:146	D00996
Interactive	hsa:146	D01020
Interactive	hsa:146	D01022
Interactive	hsa:146	D01024
Interactive	hsa:146	D01051
Interactive	hsa:146	D01358
Interactive	hsa:146	D01603
Interactive	hsa:146	D01713
Interactive	hsa:146	D01965
Interactive	hsa:146	D02149
Interactive	hsa:146	D02234
Interactive	hsa:146	D02237
Interactive	hsa:146	D02356
Interactive	hsa:146	D02361
Interactive	hsa:147	D00255
Interactive	hsa:147	D00281
Interactive	hsa:147	D00283
Interactive	hsa:147	D00426
Interactive	hsa:147	D00437
Interactive	hsa:147	D00454
Interactive	hsa:147	D00509
Interactive	hsa:147	D00513

Interactive	hsa:147	D00607
Interactive	hsa:147	D00609
Interactive	hsa:147	D00996
Interactive	hsa:147	D01020
Interactive	hsa:147	D01022
Interactive	hsa:147	D01024
Interactive	hsa:147	D01051
Interactive	hsa:147	D01358
Interactive	hsa:147	D01603
Interactive	hsa:147	D01713
Interactive	hsa:147	D01965
Interactive	hsa:147	D02149
Interactive	hsa:147	D02234
Interactive	hsa:147	D02237
Interactive	hsa:147	D02356
Interactive	hsa:147	D02361
Interactive	hsa:148	D00095
Interactive	hsa:148	D00255
Interactive	hsa:148	D00281
Interactive	hsa:148	D00283
Interactive	hsa:148	D00426
Interactive	hsa:148	D00437
Interactive	hsa:148	D00454
Interactive	hsa:148	D00494
Interactive	hsa:148	D00503
Interactive	hsa:148	D00509
Interactive	hsa:148	D00513
Interactive	hsa:148	D00607
Interactive	hsa:148	D00609
Interactive	hsa:148	D00954
Interactive	hsa:148	D00965
Interactive	hsa:148	D00996
Interactive	hsa:148	D01020
Interactive	hsa:148	D01022
Interactive	hsa:148	D01024
Interactive	hsa:148	D01051
Interactive	hsa:148	D01358
Interactive	hsa:148	D01603
Interactive	hsa:148	D01692
Interactive	hsa:148	D01713
Interactive	hsa:148	D01965
Interactive	hsa:148	D02149
Interactive	hsa:148	D02234
Interactive	hsa:148	D02237
Interactive	hsa:148	D02356
Interactive	hsa:148	D02361
Interactive	hsa:148	D02566

Interactive	hsa:148	D02910
Interactive	hsa:150	D00136
Interactive	hsa:150	D00255
Interactive	hsa:150	D00270
Interactive	hsa:150	D00281
Interactive	hsa:150	D00283
Interactive	hsa:150	D00332
Interactive	hsa:150	D00437
Interactive	hsa:150	D00454
Interactive	hsa:150	D00509
Interactive	hsa:150	D00513
Interactive	hsa:150	D00514
Interactive	hsa:150	D00563
Interactive	hsa:150	D00604
Interactive	hsa:150	D00606
Interactive	hsa:150	D00607
Interactive	hsa:150	D00609
Interactive	hsa:150	D00613
Interactive	hsa:150	D00996
Interactive	hsa:150	D01022
Interactive	hsa:150	D01603
Interactive	hsa:150	D01713
Interactive	hsa:150	D02076
Interactive	hsa:150	D02149
Interactive	hsa:150	D02237
Interactive	hsa:150	D02349
Interactive	hsa:150	D02356
Interactive	hsa:150	D03274
Interactive	hsa:150	D04034
Interactive	hsa:150	D04375
Interactive	hsa:151	D00136
Interactive	hsa:151	D00255
Interactive	hsa:151	D00270
Interactive	hsa:151	D00281
Interactive	hsa:151	D00283
Interactive	hsa:151	D00437
Interactive	hsa:151	D00454
Interactive	hsa:151	D00509
Interactive	hsa:151	D00513
Interactive	hsa:151	D00563
Interactive	hsa:151	D00604
Interactive	hsa:151	D00606
Interactive	hsa:151	D00607
Interactive	hsa:151	D00609
Interactive	hsa:151	D00613
Interactive	hsa:151	D00996
Interactive	hsa:151	D01022

Interactive	hsa:151	D01603
Interactive	hsa:151	D01713
Interactive	hsa:151	D02076
Interactive	hsa:151	D02149
Interactive	hsa:151	D02237
Interactive	hsa:151	D02356
Interactive	hsa:151	D03274
Interactive	hsa:151	D04034
Interactive	hsa:152	D00281
Interactive	hsa:152	D00509
Interactive	hsa:152	D00604
Interactive	hsa:152	D00606
Interactive	hsa:152	D00607
Interactive	hsa:152	D00609
Interactive	hsa:152	D00613
Interactive	hsa:152	D00996
Interactive	hsa:152	D01022
Interactive	hsa:152	D01603
Interactive	hsa:152	D02076
Interactive	hsa:152	D02149
Interactive	hsa:152	D02237
Interactive	hsa:152	D03274
Interactive	hsa:152	D04034
Interactive	hsa:153	D00095
Interactive	hsa:153	D00235
Interactive	hsa:153	D00255
Interactive	hsa:153	D00432
Interactive	hsa:153	D00437
Interactive	hsa:153	D00454
Interactive	hsa:153	D00483
Interactive	hsa:153	D00513
Interactive	hsa:153	D00598
Interactive	hsa:153	D00601
Interactive	hsa:153	D00632
Interactive	hsa:153	D00635
Interactive	hsa:153	D00645
Interactive	hsa:153	D00996
Interactive	hsa:153	D01390
Interactive	hsa:153	D01454
Interactive	hsa:153	D02066
Interactive	hsa:153	D02149
Interactive	hsa:153	D02150
Interactive	hsa:153	D02338
Interactive	hsa:153	D02342
Interactive	hsa:153	D02358
Interactive	hsa:153	D02374
Interactive	hsa:153	D02614

Interactive	hsa:153	D02910
Interactive	hsa:153	D03415
Interactive	hsa:153	D03490
Interactive	hsa:153	D03879
Interactive	hsa:153	D03880
Interactive	hsa:153	D03881
Interactive	hsa:153	D04625
Interactive	hsa:154	D00095
Interactive	hsa:154	D00235
Interactive	hsa:154	D00255
Interactive	hsa:154	D00432
Interactive	hsa:154	D00437
Interactive	hsa:154	D00454
Interactive	hsa:154	D00483
Interactive	hsa:154	D00513
Interactive	hsa:154	D00598
Interactive	hsa:154	D00601
Interactive	hsa:154	D00632
Interactive	hsa:154	D00635
Interactive	hsa:154	D00645
Interactive	hsa:154	D00683
Interactive	hsa:154	D00684
Interactive	hsa:154	D00687
Interactive	hsa:154	D00688
Interactive	hsa:154	D00996
Interactive	hsa:154	D01386
Interactive	hsa:154	D01390
Interactive	hsa:154	D01454
Interactive	hsa:154	D02066
Interactive	hsa:154	D02147
Interactive	hsa:154	D02149
Interactive	hsa:154	D02150
Interactive	hsa:154	D02338
Interactive	hsa:154	D02342
Interactive	hsa:154	D02359
Interactive	hsa:154	D02374
Interactive	hsa:154	D03415
Interactive	hsa:154	D03490
Interactive	hsa:154	D03879
Interactive	hsa:154	D03880
Interactive	hsa:154	D03881
Interactive	hsa:154	D05792
Interactive	hsa:155	D00255
Interactive	hsa:155	D00432
Interactive	hsa:155	D00437
Interactive	hsa:155	D00454
Interactive	hsa:155	D00483

Interactive	hsa:155	D00513
Interactive	hsa:155	D00996
Interactive	hsa:155	D01390
Interactive	hsa:155	D01454
Interactive	hsa:155	D02066
Interactive	hsa:155	D02149
Interactive	hsa:155	D02150
Interactive	hsa:155	D02338
Interactive	hsa:155	D02374
Interactive	hsa:155	D03415
Interactive	hsa:155	D03879
Interactive	hsa:1812	D00059
Interactive	hsa:1812	D00110
Interactive	hsa:1812	D00270
Interactive	hsa:1812	D00283
Interactive	hsa:1812	D00454
Interactive	hsa:1812	D00493
Interactive	hsa:1812	D00503
Interactive	hsa:1812	D00560
Interactive	hsa:1812	D00613
Interactive	hsa:1812	D00790
Interactive	hsa:1812	D01295
Interactive	hsa:1812	D02354
Interactive	hsa:1812	D02361
Interactive	hsa:1812	D02671
Interactive	hsa:1813	D00059
Interactive	hsa:1813	D00136
Interactive	hsa:1813	D00270
Interactive	hsa:1813	D00283
Interactive	hsa:1813	D00454
Interactive	hsa:1813	D00493
Interactive	hsa:1813	D00494
Interactive	hsa:1813	D00503
Interactive	hsa:1813	D00560
Interactive	hsa:1813	D00726
Interactive	hsa:1813	D00780
Interactive	hsa:1813	D00790
Interactive	hsa:1813	D00987
Interactive	hsa:1813	D01164
Interactive	hsa:1813	D01295
Interactive	hsa:1813	D01462
Interactive	hsa:1813	D01745
Interactive	hsa:1813	D02340
Interactive	hsa:1813	D02354
Interactive	hsa:1813	D02361
Interactive	hsa:1813	D02671
Interactive	hsa:1813	D03165

Interactive	hsa:1814	D00110
Interactive	hsa:1814	D00136
Interactive	hsa:1814	D00270
Interactive	hsa:1814	D00454
Interactive	hsa:1814	D00493
Interactive	hsa:1814	D00503
Interactive	hsa:1814	D00559
Interactive	hsa:1814	D00560
Interactive	hsa:1814	D00726
Interactive	hsa:1814	D01164
Interactive	hsa:1814	D01295
Interactive	hsa:1814	D02340
Interactive	hsa:1814	D02671
Interactive	hsa:1815	D00283
Interactive	hsa:1815	D00454
Interactive	hsa:1815	D02354
Interactive	hsa:1815	D02361
Interactive	hsa:1816	D00270
Interactive	hsa:1816	D00283
Interactive	hsa:1816	D00454
Interactive	hsa:1816	D00493
Interactive	hsa:1816	D00560
Interactive	hsa:1816	D00613
Interactive	hsa:1816	D01295
Interactive	hsa:1816	D02671
Interactive	hsa:185	D00400
Interactive	hsa:185	D00443
Interactive	hsa:185	D00522
Interactive	hsa:185	D00523
Interactive	hsa:185	D00627
Interactive	hsa:185	D02082
Interactive	hsa:185	D04040
Interactive	hsa:185	D05246
Interactive	hsa:1909	D01227
Interactive	hsa:1910	D01227
Interactive	hsa:222545	D01126
Interactive	hsa:222545	D02278
Interactive	hsa:222545	D02279
Interactive	hsa:23620	D01717
Interactive	hsa:2550	D00241
Interactive	hsa:2846	D00528
Interactive	hsa:2911	D00775
Interactive	hsa:2912	D00775
Interactive	hsa:2913	D00775
Interactive	hsa:2914	D00775
Interactive	hsa:2915	D00775
Interactive	hsa:2916	D00775

Interactive	hsa:2917	D00775
Interactive	hsa:2918	D00775
Interactive	hsa:2918	D01346
Interactive	hsa:3269	D00234
Interactive	hsa:3269	D00283
Interactive	hsa:3269	D00300
Interactive	hsa:3269	D00364
Interactive	hsa:3269	D00454
Interactive	hsa:3269	D00480
Interactive	hsa:3269	D00493
Interactive	hsa:3269	D00494
Interactive	hsa:3269	D00520
Interactive	hsa:3269	D00521
Interactive	hsa:3269	D00665
Interactive	hsa:3269	D00666
Interactive	hsa:3269	D01242
Interactive	hsa:3269	D01295
Interactive	hsa:3269	D01324
Interactive	hsa:3269	D01332
Interactive	hsa:3269	D01713
Interactive	hsa:3269	D01717
Interactive	hsa:3269	D01782
Interactive	hsa:3269	D02327
Interactive	hsa:3269	D02354
Interactive	hsa:3269	D02361
Interactive	hsa:3269	D02566
Interactive	hsa:3269	D03621
Interactive	hsa:3274	D00295
Interactive	hsa:3274	D00318
Interactive	hsa:3274	D00422
Interactive	hsa:3274	D00440
Interactive	hsa:3274	D00673
Interactive	hsa:3274	D01713
Interactive	hsa:3274	D03503
Interactive	hsa:3350	D00283
Interactive	hsa:3350	D00451
Interactive	hsa:3350	D00513
Interactive	hsa:3350	D00726
Interactive	hsa:3350	D01051
Interactive	hsa:3350	D01164
Interactive	hsa:3350	D01973
Interactive	hsa:3351	D00283
Interactive	hsa:3351	D00415
Interactive	hsa:3351	D00451
Interactive	hsa:3351	D00513
Interactive	hsa:3351	D00675
Interactive	hsa:3351	D00676

Interactive	hsa:3351	D00726
Interactive	hsa:3351	D01973
Interactive	hsa:3351	D02826
Interactive	hsa:3351	D05740
Interactive	hsa:3352	D00283
Interactive	hsa:3352	D00415
Interactive	hsa:3352	D00451
Interactive	hsa:3352	D00513
Interactive	hsa:3352	D00675
Interactive	hsa:3352	D00676
Interactive	hsa:3352	D00726
Interactive	hsa:3352	D01973
Interactive	hsa:3352	D02826
Interactive	hsa:3352	D05740
Interactive	hsa:3354	D00283
Interactive	hsa:3354	D00451
Interactive	hsa:3354	D00513
Interactive	hsa:3354	D00726
Interactive	hsa:3354	D01973
Interactive	hsa:3355	D00283
Interactive	hsa:3355	D00451
Interactive	hsa:3355	D00513
Interactive	hsa:3355	D00674
Interactive	hsa:3355	D00676
Interactive	hsa:3355	D00726
Interactive	hsa:3355	D01973
Interactive	hsa:3356	D00270
Interactive	hsa:3356	D00283
Interactive	hsa:3356	D00426
Interactive	hsa:3356	D00451
Interactive	hsa:3356	D00454
Interactive	hsa:3356	D00493
Interactive	hsa:3356	D00494
Interactive	hsa:3356	D00513
Interactive	hsa:3356	D00563
Interactive	hsa:3356	D00726
Interactive	hsa:3356	D01051
Interactive	hsa:3356	D01164
Interactive	hsa:3356	D01358
Interactive	hsa:3356	D01713
Interactive	hsa:3356	D02340
Interactive	hsa:3356	D02354
Interactive	hsa:3356	D02357
Interactive	hsa:3356	D02361
Interactive	hsa:3356	D02671
Interactive	hsa:3357	D00283
Interactive	hsa:3357	D00451

Interactive	hsa:3357	D00513
Interactive	hsa:3357	D00726
Interactive	hsa:3357	D01164
Interactive	hsa:3357	D01973
Interactive	hsa:3358	D00283
Interactive	hsa:3358	D00451
Interactive	hsa:3358	D00454
Interactive	hsa:3358	D00513
Interactive	hsa:3358	D00563
Interactive	hsa:3358	D00726
Interactive	hsa:3358	D01164
Interactive	hsa:3358	D02354
Interactive	hsa:3358	D02361
Interactive	hsa:3358	D02578
Interactive	hsa:3360	D00274
Interactive	hsa:3360	D00283
Interactive	hsa:3360	D00451
Interactive	hsa:3360	D00513
Interactive	hsa:3360	D00726
Interactive	hsa:3360	D01994
Interactive	hsa:3360	D06056
Interactive	hsa:3360	D06396
Interactive	hsa:3361	D00283
Interactive	hsa:3361	D00451
Interactive	hsa:3361	D00513
Interactive	hsa:3361	D00726
Interactive	hsa:3362	D00283
Interactive	hsa:3362	D00451
Interactive	hsa:3362	D00454
Interactive	hsa:3362	D00513
Interactive	hsa:3362	D00726
Interactive	hsa:3363	D00283
Interactive	hsa:3363	D01051
Interactive	hsa:3363	D01973
Interactive	hsa:338442	D00049
Interactive	hsa:3577	D00139
Interactive	hsa:3577	D00225
Interactive	hsa:3577	D00380
Interactive	hsa:3577	D00394
Interactive	hsa:3577	D00410
Interactive	hsa:3577	D00437
Interactive	hsa:3577	D00528
Interactive	hsa:3577	D00542
Interactive	hsa:3577	D00574
Interactive	hsa:3577	D01071
Interactive	hsa:4543	D02578
Interactive	hsa:4985	D00560

Interactive	hsa:4986	D00110
Interactive	hsa:4986	D00837
Interactive	hsa:4986	D00838
Interactive	hsa:4988	D00301
Interactive	hsa:4988	D00498
Interactive	hsa:4988	D00837
Interactive	hsa:4988	D00838
Interactive	hsa:4988	D00845
Interactive	hsa:4988	D04716
Interactive	hsa:4988	D05113
Interactive	hsa:4988	D05938
Interactive	hsa:5028	D00528
Interactive	hsa:5029	D00528
Interactive	hsa:5030	D00528
Interactive	hsa:5031	D00528
Interactive	hsa:5032	D00528
Interactive	hsa:552	D01236
Interactive	hsa:554	D01236
Interactive	hsa:56413	D00411
Interactive	hsa:57105	D00411
Interactive	hsa:5724	D01652
Interactive	hsa:5731	D00180
Interactive	hsa:5731	D00682
Interactive	hsa:5731	D02721
Interactive	hsa:5731	D02725
Interactive	hsa:5732	D00180
Interactive	hsa:5732	D00419
Interactive	hsa:5732	D03187
Interactive	hsa:5733	D00419
Interactive	hsa:5733	D01891
Interactive	hsa:5733	D02725
Interactive	hsa:5737	D00356
Interactive	hsa:5737	D01352
Interactive	hsa:5737	D01964
Interactive	hsa:5739	D00079
Interactive	hsa:5739	D00180
Interactive	hsa:5739	D01352
Interactive	hsa:5739	D02721
Interactive	hsa:59340	D00234
Interactive	hsa:59340	D00283
Interactive	hsa:59340	D00300
Interactive	hsa:59340	D00318
Interactive	hsa:59340	D00494
Interactive	hsa:59340	D02566
Interactive	hsa:6010	D05341
Interactive	hsa:64805	D00106
Interactive	hsa:64805	D00528

Interactive	hsa:64805	D00769
Interactive	hsa:6751	D00442
Interactive	hsa:6752	D00442
Interactive	hsa:6752	D02250
Interactive	hsa:6753	D02250
Interactive	hsa:6755	D02250
Interactive	hsa:6915	D00336
Interactive	hsa:6915	D03642
Interactive	hsa:7201	D01925
Interactive	hsa:7201	D02007
Interactive	hsa:7201	D02588
Interactive	hsa:8843	D00499
Interactive	hsa:8843	D00524
Interactive	hsa:8843	D00726
Interactive	hsa:9052	D00094
Interactive	hsa:9283	D01441
Interactive	hsa:9934	D00528
Non-interactive	hsa:10161	D00235
Non-interactive	hsa:10161	D00498
Non-interactive	hsa:10161	D00540
Non-interactive	hsa:10161	D00559
Non-interactive	hsa:10161	D00632
Non-interactive	hsa:10161	D00645
Non-interactive	hsa:10161	D00666
Non-interactive	hsa:10161	D00765
Non-interactive	hsa:10161	D01269
Non-interactive	hsa:10161	D01324
Non-interactive	hsa:10161	D03274
Non-interactive	hsa:10800	D00079
Non-interactive	hsa:10800	D00106
Non-interactive	hsa:10800	D00113
Non-interactive	hsa:10800	D00332
Non-interactive	hsa:10800	D00380
Non-interactive	hsa:10800	D00400
Non-interactive	hsa:10800	D00419
Non-interactive	hsa:10800	D00480
Non-interactive	hsa:10800	D00494
Non-interactive	hsa:10800	D00523
Non-interactive	hsa:10800	D00673
Non-interactive	hsa:10800	D01071
Non-interactive	hsa:10800	D01699
Non-interactive	hsa:10800	D01712
Non-interactive	hsa:10800	D02066
Non-interactive	hsa:10800	D02150
Non-interactive	hsa:10800	D02359
Non-interactive	hsa:10800	D05113
Non-interactive	hsa:11255	D00079

Non-interactive	hsa:11255	D00255
Non-interactive	hsa:11255	D00281
Non-interactive	hsa:11255	D00336
Non-interactive	hsa:11255	D00364
Non-interactive	hsa:11255	D00410
Non-interactive	hsa:11255	D00465
Non-interactive	hsa:11255	D00503
Non-interactive	hsa:11255	D00523
Non-interactive	hsa:11255	D00528
Non-interactive	hsa:11255	D00682
Non-interactive	hsa:11255	D00838
Non-interactive	hsa:11255	D00954
Non-interactive	hsa:11255	D01269
Non-interactive	hsa:11255	D01441
Non-interactive	hsa:11255	D01603
Non-interactive	hsa:11255	D01994
Non-interactive	hsa:11255	D03210
Non-interactive	hsa:11255	D03879
Non-interactive	hsa:11255	D03880
Non-interactive	hsa:11255	D06396
Non-interactive	hsa:1128	D00234
Non-interactive	hsa:1128	D00255
Non-interactive	hsa:1128	D00318
Non-interactive	hsa:1128	D00332
Non-interactive	hsa:1128	D00371
Non-interactive	hsa:1128	D00411
Non-interactive	hsa:1128	D00422
Non-interactive	hsa:1128	D00509
Non-interactive	hsa:1128	D01051
Non-interactive	hsa:1128	D01242
Non-interactive	hsa:1128	D01346
Non-interactive	hsa:1128	D01454
Non-interactive	hsa:1128	D01603
Non-interactive	hsa:1128	D01712
Non-interactive	hsa:1128	D02342
Non-interactive	hsa:1128	D03165
Non-interactive	hsa:1128	D03880
Non-interactive	hsa:1128	D05792
Non-interactive	hsa:1128	D05938
Non-interactive	hsa:1129	D00079
Non-interactive	hsa:1129	D00300
Non-interactive	hsa:1129	D00301
Non-interactive	hsa:1129	D00306
Non-interactive	hsa:1129	D00498
Non-interactive	hsa:1129	D00520
Non-interactive	hsa:1129	D00560
Non-interactive	hsa:1129	D00673

Non-interactive	hsa:1129	D00996
Non-interactive	hsa:1129	D01051
Non-interactive	hsa:1129	D01118
Non-interactive	hsa:1129	D01745
Non-interactive	hsa:1129	D02007
Non-interactive	hsa:1129	D02149
Non-interactive	hsa:1129	D02566
Non-interactive	hsa:1129	D02910
Non-interactive	hsa:1129	D03880
Non-interactive	hsa:1131	D00059
Non-interactive	hsa:1131	D00095
Non-interactive	hsa:1131	D00270
Non-interactive	hsa:1131	D00465
Non-interactive	hsa:1131	D00493
Non-interactive	hsa:1131	D00574
Non-interactive	hsa:1131	D00646
Non-interactive	hsa:1131	D00987
Non-interactive	hsa:1131	D00996
Non-interactive	hsa:1131	D01022
Non-interactive	hsa:1131	D02082
Non-interactive	hsa:1131	D02338
Non-interactive	hsa:1131	D02358
Non-interactive	hsa:1131	D02588
Non-interactive	hsa:1131	D03210
Non-interactive	hsa:1131	D03490
Non-interactive	hsa:1132	D00095
Non-interactive	hsa:1132	D00136
Non-interactive	hsa:1132	D00270
Non-interactive	hsa:1132	D00318
Non-interactive	hsa:1132	D00400
Non-interactive	hsa:1132	D00480
Non-interactive	hsa:1132	D00523
Non-interactive	hsa:1132	D00559
Non-interactive	hsa:1132	D00563
Non-interactive	hsa:1132	D00598
Non-interactive	hsa:1132	D00609
Non-interactive	hsa:1132	D00673
Non-interactive	hsa:1132	D01352
Non-interactive	hsa:1132	D01994
Non-interactive	hsa:1132	D02237
Non-interactive	hsa:1132	D02342
Non-interactive	hsa:1132	D02884
Non-interactive	hsa:1132	D03880
Non-interactive	hsa:1132	D05113
Non-interactive	hsa:1132	D05740
Non-interactive	hsa:1133	D00232
Non-interactive	hsa:1133	D00380

Non-interactive	hsa:1133	D00410
Non-interactive	hsa:1133	D00432
Non-interactive	hsa:1133	D00483
Non-interactive	hsa:1133	D00521
Non-interactive	hsa:1133	D00542
Non-interactive	hsa:1133	D00666
Non-interactive	hsa:1133	D00765
Non-interactive	hsa:1133	D00845
Non-interactive	hsa:1133	D01118
Non-interactive	hsa:1133	D02066
Non-interactive	hsa:1133	D02149
Non-interactive	hsa:1133	D02327
Non-interactive	hsa:1133	D02374
Non-interactive	hsa:1133	D02578
Non-interactive	hsa:1133	D03274
Non-interactive	hsa:1133	D03621
Non-interactive	hsa:1133	D05246
Non-interactive	hsa:1234	D00426
Non-interactive	hsa:1234	D01020
Non-interactive	hsa:1234	D02007
Non-interactive	hsa:1234	D02234
Non-interactive	hsa:1234	D02278
Non-interactive	hsa:1234	D04375
Non-interactive	hsa:1234	D04625
Non-interactive	hsa:1234	D05938
Non-interactive	hsa:1241	D00281
Non-interactive	hsa:1241	D00480
Non-interactive	hsa:1241	D00779
Non-interactive	hsa:1241	D01386
Non-interactive	hsa:1241	D01462
Non-interactive	hsa:1241	D02359
Non-interactive	hsa:1241	D02910
Non-interactive	hsa:1241	D03490
Non-interactive	hsa:1241	D03858
Non-interactive	hsa:1268	D00180
Non-interactive	hsa:1268	D00283
Non-interactive	hsa:1268	D00790
Non-interactive	hsa:1268	D01118
Non-interactive	hsa:1268	D01462
Non-interactive	hsa:1268	D01713
Non-interactive	hsa:1268	D01965
Non-interactive	hsa:1268	D02150
Non-interactive	hsa:1268	D02234
Non-interactive	hsa:1268	D02578
Non-interactive	hsa:1268	D03880
Non-interactive	hsa:1268	D04040
Non-interactive	hsa:1268	D06056

Non-interactive	hsa:134	D00225
Non-interactive	hsa:134	D00281
Non-interactive	hsa:134	D00380
Non-interactive	hsa:134	D00627
Non-interactive	hsa:134	D00632
Non-interactive	hsa:134	D00769
Non-interactive	hsa:134	D01020
Non-interactive	hsa:134	D01227
Non-interactive	hsa:134	D01297
Non-interactive	hsa:134	D01332
Non-interactive	hsa:134	D02349
Non-interactive	hsa:134	D02725
Non-interactive	hsa:135	D00225
Non-interactive	hsa:135	D00432
Non-interactive	hsa:135	D00673
Non-interactive	hsa:135	D00779
Non-interactive	hsa:135	D00954
Non-interactive	hsa:135	D00996
Non-interactive	hsa:135	D02007
Non-interactive	hsa:135	D03880
Non-interactive	hsa:136	D00356
Non-interactive	hsa:136	D00498
Non-interactive	hsa:136	D00524
Non-interactive	hsa:136	D00769
Non-interactive	hsa:136	D00845
Non-interactive	hsa:136	D00996
Non-interactive	hsa:136	D01164
Non-interactive	hsa:136	D01352
Non-interactive	hsa:136	D01652
Non-interactive	hsa:136	D01891
Non-interactive	hsa:136	D02361
Non-interactive	hsa:136	D03858
Non-interactive	hsa:140	D00380
Non-interactive	hsa:140	D00437
Non-interactive	hsa:140	D00442
Non-interactive	hsa:140	D00540
Non-interactive	hsa:140	D00646
Non-interactive	hsa:140	D00684
Non-interactive	hsa:140	D00838
Non-interactive	hsa:140	D00965
Non-interactive	hsa:140	D01390
Non-interactive	hsa:140	D01454
Non-interactive	hsa:140	D01964
Non-interactive	hsa:140	D02070
Non-interactive	hsa:140	D02884
Non-interactive	hsa:140	D03210
Non-interactive	hsa:146	D00332

Non-interactive	hsa:146	D00674
Non-interactive	hsa:146	D02066
Non-interactive	hsa:146	D02076
Non-interactive	hsa:146	D02349
Non-interactive	hsa:146	D03880
Non-interactive	hsa:146	D04040
Non-interactive	hsa:147	D00136
Non-interactive	hsa:147	D00270
Non-interactive	hsa:147	D00394
Non-interactive	hsa:147	D00443
Non-interactive	hsa:147	D00574
Non-interactive	hsa:147	D00604
Non-interactive	hsa:147	D00632
Non-interactive	hsa:147	D00987
Non-interactive	hsa:147	D01297
Non-interactive	hsa:147	D01352
Non-interactive	hsa:147	D02150
Non-interactive	hsa:147	D05246
Non-interactive	hsa:148	D00079
Non-interactive	hsa:148	D00180
Non-interactive	hsa:148	D00234
Non-interactive	hsa:148	D00300
Non-interactive	hsa:148	D00301
Non-interactive	hsa:148	D00715
Non-interactive	hsa:148	D00780
Non-interactive	hsa:148	D01269
Non-interactive	hsa:148	D02082
Non-interactive	hsa:148	D02349
Non-interactive	hsa:148	D02354
Non-interactive	hsa:148	D03490
Non-interactive	hsa:148	D04716
Non-interactive	hsa:148	D05246
Non-interactive	hsa:150	D00295
Non-interactive	hsa:150	D00318
Non-interactive	hsa:150	D00364
Non-interactive	hsa:150	D00574
Non-interactive	hsa:150	D00775
Non-interactive	hsa:150	D01164
Non-interactive	hsa:150	D01692
Non-interactive	hsa:150	D01994
Non-interactive	hsa:150	D06396
Non-interactive	hsa:151	D00400
Non-interactive	hsa:151	D00451
Non-interactive	hsa:151	D00520
Non-interactive	hsa:151	D00574
Non-interactive	hsa:151	D00645
Non-interactive	hsa:151	D00765

Non-interactive	hsa:151	D01699
Non-interactive	hsa:151	D01973
Non-interactive	hsa:151	D02250
Non-interactive	hsa:151	D02278
Non-interactive	hsa:151	D04716
Non-interactive	hsa:151	D05341
Non-interactive	hsa:152	D00336
Non-interactive	hsa:152	D00371
Non-interactive	hsa:152	D00451
Non-interactive	hsa:152	D00514
Non-interactive	hsa:152	D00645
Non-interactive	hsa:152	D00837
Non-interactive	hsa:152	D01712
Non-interactive	hsa:152	D02007
Non-interactive	hsa:152	D02278
Non-interactive	hsa:152	D02279
Non-interactive	hsa:152	D02826
Non-interactive	hsa:152	D03503
Non-interactive	hsa:152	D03881
Non-interactive	hsa:153	D00059
Non-interactive	hsa:153	D00364
Non-interactive	hsa:153	D00394
Non-interactive	hsa:153	D00765
Non-interactive	hsa:153	D00954
Non-interactive	hsa:153	D02361
Non-interactive	hsa:153	D03187
Non-interactive	hsa:153	D04040
Non-interactive	hsa:154	D00281
Non-interactive	hsa:154	D00306
Non-interactive	hsa:154	D00682
Non-interactive	hsa:154	D01022
Non-interactive	hsa:154	D01071
Non-interactive	hsa:154	D02358
Non-interactive	hsa:154	D02725
Non-interactive	hsa:155	D00235
Non-interactive	hsa:155	D00300
Non-interactive	hsa:155	D00336
Non-interactive	hsa:155	D00397
Non-interactive	hsa:155	D00410
Non-interactive	hsa:155	D01242
Non-interactive	hsa:155	D02007
Non-interactive	hsa:155	D02237
Non-interactive	hsa:155	D05246
Non-interactive	hsa:155	D06396
Non-interactive	hsa:1812	D00234
Non-interactive	hsa:1812	D00241
Non-interactive	hsa:1812	D00336

Non-interactive	hsa:1812	D00410
Non-interactive	hsa:1812	D00432
Non-interactive	hsa:1812	D00451
Non-interactive	hsa:1812	D00542
Non-interactive	hsa:1812	D00607
Non-interactive	hsa:1812	D00996
Non-interactive	hsa:1812	D01071
Non-interactive	hsa:1812	D01352
Non-interactive	hsa:1812	D01652
Non-interactive	hsa:1812	D01828
Non-interactive	hsa:1812	D02721
Non-interactive	hsa:1812	D04040
Non-interactive	hsa:1812	D06396
Non-interactive	hsa:1813	D00094
Non-interactive	hsa:1813	D00180
Non-interactive	hsa:1813	D00442
Non-interactive	hsa:1813	D00498
Non-interactive	hsa:1813	D00522
Non-interactive	hsa:1813	D01346
Non-interactive	hsa:1813	D01358
Non-interactive	hsa:1813	D02342
Non-interactive	hsa:1813	D02357
Non-interactive	hsa:1813	D02725
Non-interactive	hsa:1813	D03858
Non-interactive	hsa:1813	D04716
Non-interactive	hsa:1813	D05113
Non-interactive	hsa:1813	D05792
Non-interactive	hsa:1814	D00113
Non-interactive	hsa:1814	D00332
Non-interactive	hsa:1814	D00356
Non-interactive	hsa:1814	D00451
Non-interactive	hsa:1814	D00523
Non-interactive	hsa:1814	D00688
Non-interactive	hsa:1814	D01713
Non-interactive	hsa:1814	D01782
Non-interactive	hsa:1814	D02342
Non-interactive	hsa:1814	D03415
Non-interactive	hsa:1814	D03642
Non-interactive	hsa:1814	D05740
Non-interactive	hsa:1814	D05938
Non-interactive	hsa:1815	D00180
Non-interactive	hsa:1815	D00300
Non-interactive	hsa:1815	D00371
Non-interactive	hsa:1815	D00465
Non-interactive	hsa:1815	D00520
Non-interactive	hsa:1815	D00574
Non-interactive	hsa:1815	D00775

Non-interactive	hsa:1815	D01358
Non-interactive	hsa:1815	D01994
Non-interactive	hsa:1815	D03187
Non-interactive	hsa:1815	D03621
Non-interactive	hsa:1815	D05341
Non-interactive	hsa:1816	D00234
Non-interactive	hsa:1816	D00422
Non-interactive	hsa:1816	D00432
Non-interactive	hsa:1816	D00480
Non-interactive	hsa:1816	D00494
Non-interactive	hsa:1816	D00574
Non-interactive	hsa:1816	D00632
Non-interactive	hsa:1816	D00676
Non-interactive	hsa:1816	D01051
Non-interactive	hsa:1816	D01891
Non-interactive	hsa:1816	D01965
Non-interactive	hsa:1816	D02349
Non-interactive	hsa:1816	D02356
Non-interactive	hsa:1816	D03274
Non-interactive	hsa:1816	D03490
Non-interactive	hsa:1816	D05740
Non-interactive	hsa:185	D00255
Non-interactive	hsa:185	D00380
Non-interactive	hsa:185	D00480
Non-interactive	hsa:185	D00493
Non-interactive	hsa:185	D00521
Non-interactive	hsa:185	D00645
Non-interactive	hsa:185	D00682
Non-interactive	hsa:185	D00775
Non-interactive	hsa:185	D00780
Non-interactive	hsa:185	D00790
Non-interactive	hsa:185	D01236
Non-interactive	hsa:185	D01297
Non-interactive	hsa:185	D01652
Non-interactive	hsa:185	D01871
Non-interactive	hsa:185	D02358
Non-interactive	hsa:185	D02826
Non-interactive	hsa:1909	D00113
Non-interactive	hsa:1909	D00274
Non-interactive	hsa:1909	D00432
Non-interactive	hsa:1909	D00451
Non-interactive	hsa:1909	D00542
Non-interactive	hsa:1909	D00666
Non-interactive	hsa:1909	D01346
Non-interactive	hsa:1909	D01441
Non-interactive	hsa:1909	D01717
Non-interactive	hsa:1909	D01782

Non-interactive	hsa:1909	D02354
Non-interactive	hsa:1909	D02566
Non-interactive	hsa:1909	D03642
Non-interactive	hsa:1909	D03654
Non-interactive	hsa:1909	D04006
Non-interactive	hsa:1909	D04375
Non-interactive	hsa:1909	D05740
Non-interactive	hsa:1910	D00274
Non-interactive	hsa:1910	D00295
Non-interactive	hsa:1910	D00364
Non-interactive	hsa:1910	D00422
Non-interactive	hsa:1910	D01164
Non-interactive	hsa:1910	D02237
Non-interactive	hsa:1910	D05341
Non-interactive	hsa:222545	D00079
Non-interactive	hsa:222545	D00139
Non-interactive	hsa:222545	D00295
Non-interactive	hsa:222545	D00493
Non-interactive	hsa:222545	D00521
Non-interactive	hsa:222545	D00674
Non-interactive	hsa:222545	D00675
Non-interactive	hsa:222545	D00765
Non-interactive	hsa:222545	D00775
Non-interactive	hsa:222545	D00845
Non-interactive	hsa:222545	D01103
Non-interactive	hsa:222545	D01236
Non-interactive	hsa:222545	D01242
Non-interactive	hsa:222545	D01454
Non-interactive	hsa:222545	D02150
Non-interactive	hsa:222545	D02359
Non-interactive	hsa:222545	D05938
Non-interactive	hsa:23620	D00234
Non-interactive	hsa:23620	D00235
Non-interactive	hsa:23620	D00559
Non-interactive	hsa:23620	D00604
Non-interactive	hsa:23620	D00683
Non-interactive	hsa:23620	D00837
Non-interactive	hsa:23620	D01297
Non-interactive	hsa:23620	D01603
Non-interactive	hsa:23620	D01692
Non-interactive	hsa:23620	D01994
Non-interactive	hsa:23620	D02250
Non-interactive	hsa:23620	D02566
Non-interactive	hsa:23620	D03490
Non-interactive	hsa:23620	D04625
Non-interactive	hsa:23620	D06056
Non-interactive	hsa:2550	D00283

Non-interactive	hsa:2550	D00394
Non-interactive	hsa:2550	D00632
Non-interactive	hsa:2550	D00760
Non-interactive	hsa:2550	D01024
Non-interactive	hsa:2550	D01699
Non-interactive	hsa:2550	D02361
Non-interactive	hsa:2550	D02566
Non-interactive	hsa:2550	D03621
Non-interactive	hsa:2846	D00139
Non-interactive	hsa:2846	D00234
Non-interactive	hsa:2846	D00270
Non-interactive	hsa:2846	D00274
Non-interactive	hsa:2846	D00442
Non-interactive	hsa:2846	D00480
Non-interactive	hsa:2846	D00525
Non-interactive	hsa:2846	D00673
Non-interactive	hsa:2846	D00845
Non-interactive	hsa:2846	D00996
Non-interactive	hsa:2846	D01022
Non-interactive	hsa:2846	D01891
Non-interactive	hsa:2846	D02349
Non-interactive	hsa:2846	D02721
Non-interactive	hsa:2911	D00301
Non-interactive	hsa:2911	D00318
Non-interactive	hsa:2911	D00371
Non-interactive	hsa:2911	D00480
Non-interactive	hsa:2911	D00483
Non-interactive	hsa:2911	D00514
Non-interactive	hsa:2911	D00542
Non-interactive	hsa:2911	D00676
Non-interactive	hsa:2911	D00996
Non-interactive	hsa:2911	D01297
Non-interactive	hsa:2911	D01462
Non-interactive	hsa:2911	D02338
Non-interactive	hsa:2912	D00480
Non-interactive	hsa:2912	D00528
Non-interactive	hsa:2912	D00540
Non-interactive	hsa:2912	D00627
Non-interactive	hsa:2912	D00665
Non-interactive	hsa:2912	D01692
Non-interactive	hsa:2912	D01699
Non-interactive	hsa:2912	D02150
Non-interactive	hsa:2912	D04034
Non-interactive	hsa:2912	D04716
Non-interactive	hsa:2913	D00110
Non-interactive	hsa:2913	D00113
Non-interactive	hsa:2913	D00380

Non-interactive	hsa:2913	D00514
Non-interactive	hsa:2913	D01699
Non-interactive	hsa:2913	D02374
Non-interactive	hsa:2913	D02566
Non-interactive	hsa:2913	D03879
Non-interactive	hsa:2914	D00180
Non-interactive	hsa:2914	D00498
Non-interactive	hsa:2914	D00604
Non-interactive	hsa:2914	D00682
Non-interactive	hsa:2914	D01352
Non-interactive	hsa:2914	D01386
Non-interactive	hsa:2914	D01712
Non-interactive	hsa:2914	D01713
Non-interactive	hsa:2914	D01717
Non-interactive	hsa:2914	D01973
Non-interactive	hsa:2914	D02066
Non-interactive	hsa:2914	D02250
Non-interactive	hsa:2914	D02340
Non-interactive	hsa:2914	D02358
Non-interactive	hsa:2914	D02614
Non-interactive	hsa:2914	D03654
Non-interactive	hsa:2914	D03880
Non-interactive	hsa:2915	D00301
Non-interactive	hsa:2915	D00443
Non-interactive	hsa:2915	D00574
Non-interactive	hsa:2915	D00613
Non-interactive	hsa:2915	D00726
Non-interactive	hsa:2915	D01164
Non-interactive	hsa:2915	D01390
Non-interactive	hsa:2915	D01603
Non-interactive	hsa:2915	D01717
Non-interactive	hsa:2915	D02278
Non-interactive	hsa:2915	D02349
Non-interactive	hsa:2915	D03274
Non-interactive	hsa:2916	D00049
Non-interactive	hsa:2916	D00095
Non-interactive	hsa:2916	D00113
Non-interactive	hsa:2916	D00306
Non-interactive	hsa:2916	D00503
Non-interactive	hsa:2916	D00598
Non-interactive	hsa:2916	D00779
Non-interactive	hsa:2916	D01324
Non-interactive	hsa:2916	D01699
Non-interactive	hsa:2916	D01925
Non-interactive	hsa:2916	D02150
Non-interactive	hsa:2916	D02354
Non-interactive	hsa:2916	D02356

Non-interactive	hsa:2916	D02361
Non-interactive	hsa:2916	D02374
Non-interactive	hsa:2916	D06396
Non-interactive	hsa:2917	D00180
Non-interactive	hsa:2917	D00235
Non-interactive	hsa:2917	D00300
Non-interactive	hsa:2917	D00332
Non-interactive	hsa:2917	D00422
Non-interactive	hsa:2917	D00542
Non-interactive	hsa:2917	D00779
Non-interactive	hsa:2917	D01051
Non-interactive	hsa:2917	D01652
Non-interactive	hsa:2917	D01717
Non-interactive	hsa:2917	D01745
Non-interactive	hsa:2917	D02066
Non-interactive	hsa:2917	D02342
Non-interactive	hsa:2917	D02361
Non-interactive	hsa:2917	D02566
Non-interactive	hsa:2917	D02884
Non-interactive	hsa:2917	D03858
Non-interactive	hsa:2918	D00235
Non-interactive	hsa:2918	D00241
Non-interactive	hsa:2918	D00306
Non-interactive	hsa:2918	D00318
Non-interactive	hsa:2918	D00454
Non-interactive	hsa:2918	D00522
Non-interactive	hsa:2918	D00574
Non-interactive	hsa:2918	D00688
Non-interactive	hsa:2918	D01925
Non-interactive	hsa:2918	D02149
Non-interactive	hsa:2918	D02349
Non-interactive	hsa:2918	D03858
Non-interactive	hsa:2918	D04006
Non-interactive	hsa:2918	D04034
Non-interactive	hsa:2918	D06056
Non-interactive	hsa:3269	D00110
Non-interactive	hsa:3269	D00225
Non-interactive	hsa:3269	D00270
Non-interactive	hsa:3269	D00503
Non-interactive	hsa:3269	D00514
Non-interactive	hsa:3269	D00525
Non-interactive	hsa:3269	D00607
Non-interactive	hsa:3269	D00635
Non-interactive	hsa:3269	D00996
Non-interactive	hsa:3269	D02007
Non-interactive	hsa:3269	D02234
Non-interactive	hsa:3269	D02250

Non-interactive	hsa:3269	D02340
Non-interactive	hsa:3269	D02578
Non-interactive	hsa:3269	D06396
Non-interactive	hsa:3274	D00095
Non-interactive	hsa:3274	D00540
Non-interactive	hsa:3274	D00609
Non-interactive	hsa:3274	D00674
Non-interactive	hsa:3274	D00775
Non-interactive	hsa:3274	D01227
Non-interactive	hsa:3274	D02237
Non-interactive	hsa:3274	D02250
Non-interactive	hsa:3274	D02278
Non-interactive	hsa:3274	D05740
Non-interactive	hsa:3350	D00394
Non-interactive	hsa:3350	D00432
Non-interactive	hsa:3350	D00465
Non-interactive	hsa:3350	D00574
Non-interactive	hsa:3350	D00665
Non-interactive	hsa:3350	D00674
Non-interactive	hsa:3350	D00675
Non-interactive	hsa:3350	D00676
Non-interactive	hsa:3350	D01024
Non-interactive	hsa:3350	D02082
Non-interactive	hsa:3350	D02150
Non-interactive	hsa:3350	D02278
Non-interactive	hsa:3351	D00079
Non-interactive	hsa:3351	D00394
Non-interactive	hsa:3351	D00509
Non-interactive	hsa:3351	D00523
Non-interactive	hsa:3351	D00559
Non-interactive	hsa:3351	D00838
Non-interactive	hsa:3351	D01925
Non-interactive	hsa:3351	D02082
Non-interactive	hsa:3351	D02234
Non-interactive	hsa:3351	D04625
Non-interactive	hsa:3352	D00059
Non-interactive	hsa:3352	D00394
Non-interactive	hsa:3352	D00426
Non-interactive	hsa:3352	D00432
Non-interactive	hsa:3352	D00498
Non-interactive	hsa:3352	D00521
Non-interactive	hsa:3352	D00574
Non-interactive	hsa:3352	D00613
Non-interactive	hsa:3352	D00682
Non-interactive	hsa:3352	D01713
Non-interactive	hsa:3352	D01828
Non-interactive	hsa:3352	D02340

Non-interactive	hsa:3352	D03490
Non-interactive	hsa:3354	D00281
Non-interactive	hsa:3354	D00397
Non-interactive	hsa:3354	D00411
Non-interactive	hsa:3354	D00422
Non-interactive	hsa:3354	D00665
Non-interactive	hsa:3354	D00954
Non-interactive	hsa:3354	D00996
Non-interactive	hsa:3354	D01020
Non-interactive	hsa:3354	D01024
Non-interactive	hsa:3354	D01390
Non-interactive	hsa:3354	D01652
Non-interactive	hsa:3354	D01745
Non-interactive	hsa:3354	D01828
Non-interactive	hsa:3354	D02671
Non-interactive	hsa:3354	D04034
Non-interactive	hsa:3355	D00234
Non-interactive	hsa:3355	D00235
Non-interactive	hsa:3355	D00301
Non-interactive	hsa:3355	D00318
Non-interactive	hsa:3355	D00397
Non-interactive	hsa:3355	D00465
Non-interactive	hsa:3355	D00523
Non-interactive	hsa:3355	D00560
Non-interactive	hsa:3355	D00996
Non-interactive	hsa:3355	D01118
Non-interactive	hsa:3355	D03621
Non-interactive	hsa:3355	D03881
Non-interactive	hsa:3356	D00227
Non-interactive	hsa:3356	D00232
Non-interactive	hsa:3356	D00371
Non-interactive	hsa:3356	D00688
Non-interactive	hsa:3356	D01126
Non-interactive	hsa:3356	D01324
Non-interactive	hsa:3356	D01386
Non-interactive	hsa:3356	D01441
Non-interactive	hsa:3356	D02150
Non-interactive	hsa:3356	D02250
Non-interactive	hsa:3356	D02342
Non-interactive	hsa:3356	D02614
Non-interactive	hsa:3356	D02826
Non-interactive	hsa:3356	D03858
Non-interactive	hsa:3356	D03879
Non-interactive	hsa:3356	D03880
Non-interactive	hsa:3357	D00227
Non-interactive	hsa:3357	D00394
Non-interactive	hsa:3357	D00493

Non-interactive	hsa:3357	D00514
Non-interactive	hsa:3357	D00563
Non-interactive	hsa:3357	D00665
Non-interactive	hsa:3357	D01227
Non-interactive	hsa:3357	D01324
Non-interactive	hsa:3357	D01713
Non-interactive	hsa:3357	D01891
Non-interactive	hsa:3357	D02066
Non-interactive	hsa:3357	D02082
Non-interactive	hsa:3357	D02358
Non-interactive	hsa:3357	D02910
Non-interactive	hsa:3357	D03415
Non-interactive	hsa:3358	D00106
Non-interactive	hsa:3358	D00235
Non-interactive	hsa:3358	D00443
Non-interactive	hsa:3358	D00676
Non-interactive	hsa:3358	D00688
Non-interactive	hsa:3358	D00769
Non-interactive	hsa:3358	D01352
Non-interactive	hsa:3358	D01925
Non-interactive	hsa:3358	D02007
Non-interactive	hsa:3358	D02070
Non-interactive	hsa:3358	D02671
Non-interactive	hsa:3358	D03621
Non-interactive	hsa:3358	D04040
Non-interactive	hsa:3358	D04716
Non-interactive	hsa:3360	D00270
Non-interactive	hsa:3360	D00295
Non-interactive	hsa:3360	D00432
Non-interactive	hsa:3360	D00521
Non-interactive	hsa:3360	D00540
Non-interactive	hsa:3360	D00687
Non-interactive	hsa:3360	D00954
Non-interactive	hsa:3360	D01051
Non-interactive	hsa:3360	D01236
Non-interactive	hsa:3360	D01352
Non-interactive	hsa:3360	D01441
Non-interactive	hsa:3360	D01965
Non-interactive	hsa:3360	D02070
Non-interactive	hsa:3360	D02338
Non-interactive	hsa:3360	D03621
Non-interactive	hsa:3360	D03642
Non-interactive	hsa:3360	D05938
Non-interactive	hsa:3361	D00306
Non-interactive	hsa:3361	D00559
Non-interactive	hsa:3361	D00604
Non-interactive	hsa:3361	D00627

Non-interactive	hsa:3361	D00632
Non-interactive	hsa:3361	D00675
Non-interactive	hsa:3361	D02007
Non-interactive	hsa:3361	D02076
Non-interactive	hsa:3361	D02234
Non-interactive	hsa:3361	D02237
Non-interactive	hsa:3361	D02250
Non-interactive	hsa:3361	D02356
Non-interactive	hsa:3361	D02614
Non-interactive	hsa:3362	D00113
Non-interactive	hsa:3362	D00234
Non-interactive	hsa:3362	D00295
Non-interactive	hsa:3362	D00356
Non-interactive	hsa:3362	D00415
Non-interactive	hsa:3362	D00432
Non-interactive	hsa:3362	D00523
Non-interactive	hsa:3362	D00560
Non-interactive	hsa:3362	D00613
Non-interactive	hsa:3362	D00665
Non-interactive	hsa:3362	D00684
Non-interactive	hsa:3362	D00775
Non-interactive	hsa:3362	D00838
Non-interactive	hsa:3362	D01051
Non-interactive	hsa:3362	D01441
Non-interactive	hsa:3362	D01454
Non-interactive	hsa:3362	D01891
Non-interactive	hsa:3362	D02342
Non-interactive	hsa:3362	D03210
Non-interactive	hsa:3362	D04625
Non-interactive	hsa:3362	D05740
Non-interactive	hsa:3363	D00095
Non-interactive	hsa:3363	D00295
Non-interactive	hsa:3363	D00442
Non-interactive	hsa:3363	D00513
Non-interactive	hsa:3363	D00525
Non-interactive	hsa:3363	D00540
Non-interactive	hsa:3363	D00607
Non-interactive	hsa:3363	D00675
Non-interactive	hsa:3363	D00682
Non-interactive	hsa:3363	D01103
Non-interactive	hsa:3363	D01441
Non-interactive	hsa:3363	D01699
Non-interactive	hsa:3363	D01828
Non-interactive	hsa:3363	D02566
Non-interactive	hsa:3363	D02826
Non-interactive	hsa:338442	D00283
Non-interactive	hsa:338442	D00437

Non-interactive	hsa:338442	D00606
Non-interactive	hsa:338442	D02070
Non-interactive	hsa:338442	D02076
Non-interactive	hsa:338442	D02278
Non-interactive	hsa:338442	D02361
Non-interactive	hsa:338442	D04034
Non-interactive	hsa:338442	D05246
Non-interactive	hsa:338442	D05740
Non-interactive	hsa:3577	D00301
Non-interactive	hsa:3577	D00419
Non-interactive	hsa:3577	D00443
Non-interactive	hsa:3577	D00524
Non-interactive	hsa:3577	D01022
Non-interactive	hsa:3577	D01242
Non-interactive	hsa:3577	D01324
Non-interactive	hsa:3577	D01891
Non-interactive	hsa:3577	D02076
Non-interactive	hsa:3577	D02578
Non-interactive	hsa:3577	D02721
Non-interactive	hsa:4543	D00110
Non-interactive	hsa:4543	D00306
Non-interactive	hsa:4543	D00380
Non-interactive	hsa:4543	D00494
Non-interactive	hsa:4543	D00498
Non-interactive	hsa:4543	D00609
Non-interactive	hsa:4543	D00665
Non-interactive	hsa:4543	D01126
Non-interactive	hsa:4543	D01164
Non-interactive	hsa:4543	D01454
Non-interactive	hsa:4543	D01745
Non-interactive	hsa:4543	D01782
Non-interactive	hsa:4543	D01965
Non-interactive	hsa:4543	D02614
Non-interactive	hsa:4985	D00110
Non-interactive	hsa:4985	D00227
Non-interactive	hsa:4985	D00509
Non-interactive	hsa:4985	D00606
Non-interactive	hsa:4985	D00609
Non-interactive	hsa:4985	D00688
Non-interactive	hsa:4985	D01994
Non-interactive	hsa:4985	D02007
Non-interactive	hsa:4985	D02588
Non-interactive	hsa:4985	D02614
Non-interactive	hsa:4985	D03165
Non-interactive	hsa:4985	D03642
Non-interactive	hsa:4985	D04006
Non-interactive	hsa:4986	D00049

Non-interactive	hsa:4986	D00281
Non-interactive	hsa:4986	D00415
Non-interactive	hsa:4986	D00443
Non-interactive	hsa:4986	D00465
Non-interactive	hsa:4986	D00528
Non-interactive	hsa:4986	D00607
Non-interactive	hsa:4986	D00726
Non-interactive	hsa:4986	D00790
Non-interactive	hsa:4986	D01269
Non-interactive	hsa:4986	D01297
Non-interactive	hsa:4986	D01692
Non-interactive	hsa:4986	D01745
Non-interactive	hsa:4986	D02070
Non-interactive	hsa:4986	D02147
Non-interactive	hsa:4986	D04006
Non-interactive	hsa:4988	D00095
Non-interactive	hsa:4988	D00110
Non-interactive	hsa:4988	D00139
Non-interactive	hsa:4988	D00437
Non-interactive	hsa:4988	D00465
Non-interactive	hsa:4988	D00524
Non-interactive	hsa:4988	D00560
Non-interactive	hsa:4988	D00675
Non-interactive	hsa:4988	D00726
Non-interactive	hsa:4988	D00790
Non-interactive	hsa:4988	D01295
Non-interactive	hsa:4988	D01346
Non-interactive	hsa:4988	D01386
Non-interactive	hsa:4988	D02278
Non-interactive	hsa:4988	D02721
Non-interactive	hsa:4988	D03415
Non-interactive	hsa:4988	D03621
Non-interactive	hsa:4988	D04006
Non-interactive	hsa:5028	D00225
Non-interactive	hsa:5028	D00270
Non-interactive	hsa:5028	D00604
Non-interactive	hsa:5028	D00779
Non-interactive	hsa:5028	D00987
Non-interactive	hsa:5028	D02082
Non-interactive	hsa:5028	D02349
Non-interactive	hsa:5029	D00059
Non-interactive	hsa:5029	D00498
Non-interactive	hsa:5029	D00521
Non-interactive	hsa:5029	D00688
Non-interactive	hsa:5029	D00779
Non-interactive	hsa:5029	D00790
Non-interactive	hsa:5029	D01164

Non-interactive	hsa:5029	D01236
Non-interactive	hsa:5029	D01441
Non-interactive	hsa:5029	D02149
Non-interactive	hsa:5029	D02340
Non-interactive	hsa:5029	D02725
Non-interactive	hsa:5030	D00079
Non-interactive	hsa:5030	D00241
Non-interactive	hsa:5030	D00364
Non-interactive	hsa:5030	D00426
Non-interactive	hsa:5030	D00775
Non-interactive	hsa:5030	D01227
Non-interactive	hsa:5030	D01269
Non-interactive	hsa:5030	D01692
Non-interactive	hsa:5030	D02342
Non-interactive	hsa:5030	D02354
Non-interactive	hsa:5030	D02578
Non-interactive	hsa:5030	D02725
Non-interactive	hsa:5030	D03490
Non-interactive	hsa:5031	D00227
Non-interactive	hsa:5031	D00274
Non-interactive	hsa:5031	D00432
Non-interactive	hsa:5031	D00499
Non-interactive	hsa:5031	D00522
Non-interactive	hsa:5031	D00635
Non-interactive	hsa:5031	D00682
Non-interactive	hsa:5031	D00684
Non-interactive	hsa:5031	D00838
Non-interactive	hsa:5031	D01020
Non-interactive	hsa:5031	D01024
Non-interactive	hsa:5031	D01269
Non-interactive	hsa:5031	D02374
Non-interactive	hsa:5031	D02578
Non-interactive	hsa:5031	D03881
Non-interactive	hsa:5031	D04006
Non-interactive	hsa:5031	D06396
Non-interactive	hsa:5032	D00838
Non-interactive	hsa:5032	D00845
Non-interactive	hsa:5032	D01071
Non-interactive	hsa:5032	D01324
Non-interactive	hsa:5032	D01352
Non-interactive	hsa:5032	D01462
Non-interactive	hsa:5032	D01871
Non-interactive	hsa:5032	D02070
Non-interactive	hsa:5032	D02147
Non-interactive	hsa:5032	D02349
Non-interactive	hsa:5032	D02910
Non-interactive	hsa:552	D00106

Non-interactive	hsa:552	D00270
Non-interactive	hsa:552	D00422
Non-interactive	hsa:552	D00509
Non-interactive	hsa:552	D00514
Non-interactive	hsa:552	D00780
Non-interactive	hsa:552	D01118
Non-interactive	hsa:552	D01324
Non-interactive	hsa:552	D01713
Non-interactive	hsa:552	D01965
Non-interactive	hsa:552	D02076
Non-interactive	hsa:552	D02340
Non-interactive	hsa:552	D03165
Non-interactive	hsa:552	D03880
Non-interactive	hsa:554	D00300
Non-interactive	hsa:554	D00440
Non-interactive	hsa:554	D00442
Non-interactive	hsa:554	D00493
Non-interactive	hsa:554	D00499
Non-interactive	hsa:554	D01022
Non-interactive	hsa:554	D01126
Non-interactive	hsa:554	D01386
Non-interactive	hsa:554	D01454
Non-interactive	hsa:554	D01994
Non-interactive	hsa:554	D02070
Non-interactive	hsa:554	D03858
Non-interactive	hsa:554	D04006
Non-interactive	hsa:554	D04040
Non-interactive	hsa:554	D05740
Non-interactive	hsa:554	D06056
Non-interactive	hsa:56413	D00059
Non-interactive	hsa:56413	D00180
Non-interactive	hsa:56413	D00494
Non-interactive	hsa:56413	D00499
Non-interactive	hsa:56413	D00522
Non-interactive	hsa:56413	D00779
Non-interactive	hsa:56413	D01103
Non-interactive	hsa:56413	D01295
Non-interactive	hsa:56413	D01324
Non-interactive	hsa:56413	D01462
Non-interactive	hsa:56413	D01745
Non-interactive	hsa:56413	D01828
Non-interactive	hsa:56413	D02354
Non-interactive	hsa:56413	D02721
Non-interactive	hsa:56413	D03274
Non-interactive	hsa:56413	D03490
Non-interactive	hsa:56413	D04625
Non-interactive	hsa:57105	D00049

Non-interactive	hsa:57105	D00139
Non-interactive	hsa:57105	D00336
Non-interactive	hsa:57105	D00790
Non-interactive	hsa:57105	D01051
Non-interactive	hsa:57105	D01332
Non-interactive	hsa:57105	D01352
Non-interactive	hsa:57105	D01712
Non-interactive	hsa:57105	D01828
Non-interactive	hsa:57105	D02342
Non-interactive	hsa:57105	D03654
Non-interactive	hsa:57105	D03879
Non-interactive	hsa:5724	D00094
Non-interactive	hsa:5724	D00234
Non-interactive	hsa:5724	D00301
Non-interactive	hsa:5724	D00443
Non-interactive	hsa:5724	D00480
Non-interactive	hsa:5724	D00503
Non-interactive	hsa:5724	D00514
Non-interactive	hsa:5724	D00574
Non-interactive	hsa:5724	D00684
Non-interactive	hsa:5724	D00765
Non-interactive	hsa:5724	D00775
Non-interactive	hsa:5724	D00996
Non-interactive	hsa:5724	D01022
Non-interactive	hsa:5724	D02578
Non-interactive	hsa:5724	D03881
Non-interactive	hsa:5731	D00411
Non-interactive	hsa:5731	D00454
Non-interactive	hsa:5731	D00613
Non-interactive	hsa:5731	D00665
Non-interactive	hsa:5731	D00769
Non-interactive	hsa:5731	D00775
Non-interactive	hsa:5731	D01071
Non-interactive	hsa:5731	D01324
Non-interactive	hsa:5731	D01441
Non-interactive	hsa:5731	D01925
Non-interactive	hsa:5731	D01964
Non-interactive	hsa:5731	D02338
Non-interactive	hsa:5731	D02374
Non-interactive	hsa:5731	D04006
Non-interactive	hsa:5731	D05246
Non-interactive	hsa:5732	D00094
Non-interactive	hsa:5732	D00106
Non-interactive	hsa:5732	D00136
Non-interactive	hsa:5732	D00281
Non-interactive	hsa:5732	D00364
Non-interactive	hsa:5732	D00380

Non-interactive	hsa:5732	D00520
Non-interactive	hsa:5732	D01242
Non-interactive	hsa:5732	D02076
Non-interactive	hsa:5732	D02147
Non-interactive	hsa:5732	D02279
Non-interactive	hsa:5732	D02354
Non-interactive	hsa:5732	D02357
Non-interactive	hsa:5732	D02671
Non-interactive	hsa:5733	D00270
Non-interactive	hsa:5733	D00520
Non-interactive	hsa:5733	D00522
Non-interactive	hsa:5733	D00613
Non-interactive	hsa:5733	D00838
Non-interactive	hsa:5733	D01652
Non-interactive	hsa:5733	D01871
Non-interactive	hsa:5733	D02147
Non-interactive	hsa:5737	D00095
Non-interactive	hsa:5737	D00110
Non-interactive	hsa:5737	D00451
Non-interactive	hsa:5737	D00454
Non-interactive	hsa:5737	D00563
Non-interactive	hsa:5737	D00609
Non-interactive	hsa:5737	D00682
Non-interactive	hsa:5737	D00996
Non-interactive	hsa:5737	D01103
Non-interactive	hsa:5737	D01227
Non-interactive	hsa:5737	D01712
Non-interactive	hsa:5737	D01965
Non-interactive	hsa:5737	D02354
Non-interactive	hsa:5737	D02614
Non-interactive	hsa:5737	D05938
Non-interactive	hsa:5739	D00281
Non-interactive	hsa:5739	D00432
Non-interactive	hsa:5739	D00493
Non-interactive	hsa:5739	D00676
Non-interactive	hsa:5739	D01071
Non-interactive	hsa:5739	D01712
Non-interactive	hsa:5739	D02327
Non-interactive	hsa:5739	D02349
Non-interactive	hsa:5739	D02374
Non-interactive	hsa:5739	D03881
Non-interactive	hsa:5739	D05246
Non-interactive	hsa:59340	D00136
Non-interactive	hsa:59340	D00380
Non-interactive	hsa:59340	D00394
Non-interactive	hsa:59340	D00400
Non-interactive	hsa:59340	D00483

Non-interactive	hsa:59340	D00687
Non-interactive	hsa:59340	D01020
Non-interactive	hsa:59340	D01332
Non-interactive	hsa:59340	D02234
Non-interactive	hsa:59340	D02237
Non-interactive	hsa:59340	D02278
Non-interactive	hsa:59340	D02279
Non-interactive	hsa:59340	D03879
Non-interactive	hsa:6010	D00106
Non-interactive	hsa:6010	D00235
Non-interactive	hsa:6010	D00295
Non-interactive	hsa:6010	D00318
Non-interactive	hsa:6010	D00528
Non-interactive	hsa:6010	D00563
Non-interactive	hsa:6010	D02250
Non-interactive	hsa:6010	D02374
Non-interactive	hsa:6010	D03274
Non-interactive	hsa:6010	D04375
Non-interactive	hsa:64805	D00059
Non-interactive	hsa:64805	D00234
Non-interactive	hsa:64805	D00503
Non-interactive	hsa:64805	D00525
Non-interactive	hsa:64805	D01386
Non-interactive	hsa:64805	D01603
Non-interactive	hsa:64805	D02076
Non-interactive	hsa:64805	D02237
Non-interactive	hsa:64805	D02279
Non-interactive	hsa:64805	D02338
Non-interactive	hsa:64805	D02361
Non-interactive	hsa:64805	D02578
Non-interactive	hsa:64805	D02671
Non-interactive	hsa:6751	D00411
Non-interactive	hsa:6751	D00419
Non-interactive	hsa:6751	D00451
Non-interactive	hsa:6751	D00454
Non-interactive	hsa:6751	D00480
Non-interactive	hsa:6751	D00540
Non-interactive	hsa:6751	D00574
Non-interactive	hsa:6751	D00607
Non-interactive	hsa:6751	D00627
Non-interactive	hsa:6751	D00674
Non-interactive	hsa:6751	D00780
Non-interactive	hsa:6751	D00954
Non-interactive	hsa:6751	D01051
Non-interactive	hsa:6751	D01713
Non-interactive	hsa:6752	D00301
Non-interactive	hsa:6752	D00498

Non-interactive	hsa:6752	D00522
Non-interactive	hsa:6752	D00635
Non-interactive	hsa:6752	D01020
Non-interactive	hsa:6752	D01603
Non-interactive	hsa:6752	D02082
Non-interactive	hsa:6752	D02374
Non-interactive	hsa:6752	D03880
Non-interactive	hsa:6753	D00371
Non-interactive	hsa:6753	D00606
Non-interactive	hsa:6753	D00607
Non-interactive	hsa:6753	D00627
Non-interactive	hsa:6753	D00996
Non-interactive	hsa:6753	D01022
Non-interactive	hsa:6753	D01352
Non-interactive	hsa:6753	D01994
Non-interactive	hsa:6753	D02082
Non-interactive	hsa:6753	D02278
Non-interactive	hsa:6753	D02327
Non-interactive	hsa:6753	D02349
Non-interactive	hsa:6753	D02359
Non-interactive	hsa:6753	D02566
Non-interactive	hsa:6753	D04006
Non-interactive	hsa:6753	D04716
Non-interactive	hsa:6755	D00255
Non-interactive	hsa:6755	D00356
Non-interactive	hsa:6755	D00480
Non-interactive	hsa:6755	D00503
Non-interactive	hsa:6755	D00509
Non-interactive	hsa:6755	D00760
Non-interactive	hsa:6755	D00838
Non-interactive	hsa:6755	D01024
Non-interactive	hsa:6755	D01358
Non-interactive	hsa:6755	D01712
Non-interactive	hsa:6755	D01891
Non-interactive	hsa:6755	D02354
Non-interactive	hsa:6755	D02359
Non-interactive	hsa:6755	D02374
Non-interactive	hsa:6755	D02566
Non-interactive	hsa:6755	D02578
Non-interactive	hsa:6755	D05246
Non-interactive	hsa:6755	D05938
Non-interactive	hsa:6915	D00059
Non-interactive	hsa:6915	D00274
Non-interactive	hsa:6915	D00400
Non-interactive	hsa:6915	D00415
Non-interactive	hsa:6915	D00480
Non-interactive	hsa:6915	D00514

Non-interactive	hsa:6915	D00666
Non-interactive	hsa:6915	D00674
Non-interactive	hsa:6915	D00682
Non-interactive	hsa:6915	D01717
Non-interactive	hsa:6915	D01925
Non-interactive	hsa:6915	D02358
Non-interactive	hsa:6915	D02671
Non-interactive	hsa:6915	D02725
Non-interactive	hsa:6915	D03490
Non-interactive	hsa:6915	D03654
Non-interactive	hsa:6915	D03858
Non-interactive	hsa:6915	D03881
Non-interactive	hsa:6915	D05113
Non-interactive	hsa:6915	D05341
Non-interactive	hsa:7201	D00301
Non-interactive	hsa:7201	D00432
Non-interactive	hsa:7201	D00520
Non-interactive	hsa:7201	D00521
Non-interactive	hsa:7201	D00838
Non-interactive	hsa:7201	D00987
Non-interactive	hsa:7201	D01332
Non-interactive	hsa:7201	D01358
Non-interactive	hsa:7201	D02066
Non-interactive	hsa:7201	D02279
Non-interactive	hsa:7201	D03274
Non-interactive	hsa:7201	D05246
Non-interactive	hsa:8843	D00397
Non-interactive	hsa:8843	D00451
Non-interactive	hsa:8843	D00503
Non-interactive	hsa:8843	D00520
Non-interactive	hsa:8843	D00574
Non-interactive	hsa:8843	D00606
Non-interactive	hsa:8843	D00635
Non-interactive	hsa:8843	D00987
Non-interactive	hsa:8843	D01022
Non-interactive	hsa:8843	D01712
Non-interactive	hsa:8843	D01782
Non-interactive	hsa:8843	D02278
Non-interactive	hsa:8843	D02340
Non-interactive	hsa:8843	D02349
Non-interactive	hsa:8843	D03642
Non-interactive	hsa:9052	D00225
Non-interactive	hsa:9052	D00255
Non-interactive	hsa:9052	D00419
Non-interactive	hsa:9052	D00598
Non-interactive	hsa:9052	D00604
Non-interactive	hsa:9052	D00845

Non-interactive	hsa:9052	D01022
Non-interactive	hsa:9052	D01390
Non-interactive	hsa:9052	D03415
Non-interactive	hsa:9052	D03879
Non-interactive	hsa:9052	D05341
Non-interactive	hsa:9283	D00094
Non-interactive	hsa:9283	D00136
Non-interactive	hsa:9283	D00397
Non-interactive	hsa:9283	D00442
Non-interactive	hsa:9283	D00606
Non-interactive	hsa:9283	D00837
Non-interactive	hsa:9283	D01020
Non-interactive	hsa:9283	D01652
Non-interactive	hsa:9283	D01994
Non-interactive	hsa:9283	D03858
Non-interactive	hsa:9283	D04375
Non-interactive	hsa:9283	D06056
Non-interactive	hsa:9934	D00136
Non-interactive	hsa:9934	D00356
Non-interactive	hsa:9934	D00371
Non-interactive	hsa:9934	D00410
Non-interactive	hsa:9934	D00415
Non-interactive	hsa:9934	D00493
Non-interactive	hsa:9934	D00509
Non-interactive	hsa:9934	D00609
Non-interactive	hsa:9934	D00683
Non-interactive	hsa:9934	D00684
Non-interactive	hsa:9934	D00954
Non-interactive	hsa:9934	D00965
Non-interactive	hsa:9934	D01782
Non-interactive	hsa:9934	D01828
Non-interactive	hsa:9934	D02147
Non-interactive	hsa:9934	D03880
Non-interactive	hsa:9934	D03881
Non-interactive	hsa:9934	D04625
