# Supplementary material for: iGPCR-Drug: A Web Server for Predicting Interaction between GPCRs and Drugs in Cellular Networking
Source: PLoS One. 2013 Aug 27;8(8):e72234. doi: 10.1371/journal.pone.0072234 (PMC3754978; doi:10.1371/journal.pone.0072234)
Supplement: Supporting Information S2 — The fingerprints for the drug codes listed in Supporting Information S1. Each of these fingerprints is a 256-D vectors generated by the OpenBabel software downloaded from http://openbabel.org/. (PDF) [file pone.0072234.s002.pdf]

Online Supporting Information S2. The fingerprints for the drug codes listed in Online Supporting Information S1. Each of these fingerprints is a 256-D vectors generated by the OpenBabel software downloaded from http://openbabel.org/. See the text of the main paper for further explanation.

```
>D00419
00050000  01000000  00000800  00010100  00080000  00000000
00000000  00000402  00000000  00108000  00000200  40009000
14028008  00800000  00000404  00080008  00820000  00000000
00402000  02000001  00000000  00000a00  01008000  00000010
00200000  00000000  00404000  80000000  00000200  81000000
24020000  00000000

>D00415
00000002  00405808  00100300  41010710  00041080  00008000
00002000  80000004  00400b00  00090840  08300004  40008800
10008800  00400100  00001040  001e000c  00020000  02080004
00600400  00000001  40000100  18208c18  23800040  91400018
00042000  80000002  18000040  00000000  04400200  00060003
80040200  00000620

>D00411
08000012  45c00808  21080108  41b10710  000c5000  00000008
06002001  81001200  00400304  001838c0  08382004  60018800
80008802  00508101  813040c1  201c202c  0287a080  82900008
08680c00  00426205  c0000000  18028e18  21802002  8a40801c
02042000  e0001006  c8000080  4000008c  00008780  a1060003
a2044200  00010760

>D00410
00008002  00804800  00004100  00800210  00041000  00000000
00002000  00000000  00400800  00080040  00100004  40001000
03008800  00000800  40000000  00040004  00000000  02000004
00200400  00000001  c0000100  08148200  20800000  0840001c
00040000  00000000  08000000  00000000  00400000  00020002
80002000  00000500

>D00400
0001a008  01805019  40100100  00010600  00000080  08020008
00000004  22081000  00000100  000008c0  00208000  4001b000
86008882  00200000  a0080400  0008200c  10020000  02000800
04400000  10000201  80060100  18008e00  13008000  00504018
00000000  c0200000  10000000  90000040  00004104  00060001
a0044200  08002200
```

>D00397
00000002 00800800 00104100 00010600 00040040 00000000
00000000 00001000 00000080 00080840 08200004 40009000
91008802 00000000 40040400 000c000c 08000008 02000000
00a00000 00000200 c0000000 18208600 21800080 81400018
00004001 c0000000 00000000 80000000 04000100 00060003
2000a000 00000620

>D00394
08004002 00000000 00180100 00010600 20000020 00002040
01000010 00000040 00102800 08480960 08340004 40808000
00000400 00000000 00020040 08000002 0004c200 02000001
0800000a 00000001 65100100 18100000 20000000 08500010
00000800 80000000 02000008 00001080 00088000 00a60012
00000080 20000700

>D00380
00000010 00801000 01100100 02b10e00 00000008 00000000
08000000 00003000 00000000 000028c0 00202000 e0008400
00009000 00000041 80200000 00000000 02002000 02000000
00400000 00006001 80002000 18050400 00000000 08400018
00000000 80000000 80000000 1000042c 00000400 20060001
00080000 00010260

>D00371
0000002a 00801800 00080000 02010600 00080040 0000c000
00008000 00000000 06400100 800000a0 0c00800c 00000000
00009000 00400000 00280080 000c000c 00800000 00008000
00400000 00000000 80000100 10000040 21800041 00300004
00040000 c0200000 60000000 00040000 00004000 00460000
c0000000 00000280

>D00364
0008800a 0d405000 00104300 10810610 2a061020 0008a002
84082480 09001409 04400a00 001a08c8 08308004 50818a00
02008826 00200800 82020022 021e208e 01020400 0a080045
40340c00 00000201 40100100 18148d50 29c0c000 08550818
01042000 a2000083 08442040 50a08200 02400286 00060c03
84020000 20030700

>D00356
00010000 81000000 00000102 00010300 00280000 00000000
00000000 00000402 00000800 00188848 08100000 40009000

04028000 02a00000 00000400 00081008 00820000 02000000
01402000 00002001 00000120 08000a00 01008040 00400010
00200400 80000000 00404000 80000000 00000202 01020002
24020000 00000400

>D00336
00008010 09801808 21100108 02b10e00 00001008 00020100
0e000000 02003404 00000080 001828c8 08202000 e0018c00
02009806 00000041 81200401 00002008 02002000 02000400
02402000 00026201 80002000 18278710 00000000 8950001c
00002000 c0000000 80100800 9000042c 04000700 20060803
a40c0000 00030660

>D00332
00030000 01000000 00000000 00000400 00180000 00000000
00000000 00000000 00000000 00000080 00000000 40011000
04008008 00800000 00000000 00080008 00000000 00000000
02402000 00000001 00000000 10000a10 01000000 00000010
00000000 00000000 00000000 00000000 00000000 00040000
00020000 00000000

>D00318
00030002 00000200 00000800 00b00d40 09000204 0000000c
00040100 18028008 00400000 0001c003 00216a00 52040000
00010408 01044009 04032000 0004000c 05000800 02300780
00000010 01200004 00000002 10804080 20000a00 00010010
004c1000 0c108400 c0200000 00020254 0004000c 04062020
02000000 01111060

>D00306
04004001 c3000038 2000010a 80210302 80081000 c2800000
42000005 00000642 00044900 00980848 08100000 40008840
00821002 20a00084 11000401 00082008 08800000 22108020
02682000 00022a01 80000124 08000000 01008040 00400410
00200000 c0000000 00404000 80000000 20010302 00034002
24020000 00000400

>D00301
00020040 01000200 00100100 00410f00 00180040 00000010
00000000 21002840 00000800 010808c0 08302000 50019000
15008002 00880002 40040000 000c100c 08e20000 02000000
05c02000 00000201 00010120 18000a10 01800480 00500010
00000401 a0000000 00000200 50001000 00000280 0006000e
00022000 00832ea0

```
>D00300
00000000  00000000  00000100  80010600  00000090  00004000
00000000  01000000  00000100  00080840  08201000  40008000
00000000  00010000  00001008  02080008  00000000  02000000
00000000  00000000  00000000  08000020  12000000  00400010
00000400  a0002000  00080000  40000000  00000280  00060002
40000000  00000600

>D00295
00000042  40008000  21004000  00300c10  00090040  00000002
00000004  00002000  00000100  02014002  10208000  42000800
00004005  00340008  00020010  001c8004  24800800  02200300
00000000  40001080  00000102  00010000  21200600  08000000
00440000  01000000  42004000  00010220  00002014  00460000
40000001  02012200

>D00283
08208246  01840000  00100100  0c210600  00020001  80100004
18c00010  02301044  10012700  8003c843  12202004  44018c30
0200080e  c010a809  040604c0  18180080  04658808  13084002
09000002  30801200  40022000  38028100  e3004020  02401000
01082000  c8000180  42880410  9d011880  08888918  01a60000
40040081  52020200

>D00281
00008042  00000000  00000000  00000e80  00000000  00000020
00000000  00002000  00000400  00000842  10200004  44008820
02000004  40000408  000000c0  00100080  00000800  00002000
01000000  00000000  40000100  28010100  22000000  00000000
00082008  08000400  40000000  00000800  08000010  00860080
00000000  00020200

>D00274
0400800a  89005c08  20100108  82010700  00101080  02020110
06000000  22001400  00000000  001009e8  08200004  50018800
02808806  08001000  81081441  00082008  10200000  02000400
00c00000  00420601  40000000  180a8700  33001080  0050001c
00002001  c0000400  00900a00  90000001  00000300  00068811
a4040000  00022a40

>D00270
0020c002  00820200  00120000  01100600  20040000  08002000
08010010  00000000  00022000  00400d60  00244004  61008800
```

02000004 00000001 20020040 02000002 04841000 20000000
08200002 02024000 e2100000 18000900 20800000 00180019
00006401 10000100 12204010 00000080 00008200 01060010
0000a000 00030220

>D00255
04002002 00000008 20100108 00210602 00041080 02000000
06080000 00000008 00000000 00000840 00200004 40008000
00804806 00400000 01281041 020ca00c 10200000 00000004
00a00000 00060200 40000000 18048008 37800080 88000010
00002001 00000000 00000000 00800001 00000200 00060008
00040200 002002c0

>D00241
00028000 00000000 00000100 00010600 00100000 00000000
00000000 00000000 00000800 000808c0 08100000 40009800
06008024 00000000 00000000 00080008 00000000 02000000
00400008 00000001 00000100 18000b00 01000040 81400010
00002800 80000000 00040000 00000000 04000000 00060002
00000000 00020400

>D00235
04000000 00000008 20100108 00010600 00001080 02000000
02040000 00000000 00000000 00080840 08200000 40009000
01808002 00000000 41200001 00082008 10200000 02000000
00800000 00020200 00000000 18000600 07000080 89400010
00000001 80000000 00120000 00800000 04000200 00060002
00002000 00000640

>D00234
00000006 01800608 20100108 02010600 000c1040 00004012
02048000 20101000 00100190 200809e0 0920800c 50018000
00001802 00401000 012000c1 000c000c 00000000 02004100
00600000 00b20201 c0000000 5c208030 21800841 81700010
00000000 80000000 02128000 10000001 05000218 0046981a
400c1000 00002680

>D00232
00010040 01000000 00100100 00010700 00080000 00000010
00000000 20000000 00000000 00000080 00200000 50019000
04008002 00880000 00000000 00081008 00a20000 00000000
00c02000 00000201 00000020 10000a10 01000080 00100010
00000401 00000000 00000000 10001000 00000200 00040006
00020008 00002200

>D00227
0000002a 00801800 00080000 02010600 00080040 0000c000
00008000 00000000 06400100 800000a0 0c00800c 40000000
00009000 00400000 00280080 000c000c 00800000 00008000
00400000 00000000 80000100 30000040 21800041 00300004
00040000 c0200000 60000000 00040000 00004000 00460000
c0000000 00000280

>D00225
0080800a 06840019 402c0500 98010780 41000040 00880026
14840000 01201401 10000300 00090845 09010134 44068c00
02100884 08088a08 80068940 080c258c 0a208800 020a0207
00600408 00260000 40122000 2801a100 61001002 08508110
10802010 e00005c0 40084040 45021001 0000009c 00860c02
c00c0080 a4360400

>D00180
00050000 01000000 00000800 00000000 00080000 00000000
00000000 28000402 00000000 00108000 00000200 40001000
14028008 00800000 00000404 00080008 00000000 00000000
00402010 02000001 00000000 00000a00 01008000 00000010
00201000 00000400 00404000 80000000 00000000 81000000
24020000 00000000

>D00139
08000000 00200008 24080008 00000200 00011a14 00400020
02400000 40200200 02024000 40000840 00000000 00408000
4000c402 00200080 01000081 00000009 00000000 00000000
08000000 00020002 00000000 08000408 00000008 00180000
08400100 00000000 00000050 08100000 0a002200 01020000
00000000 00000000

>D00136
00028000 01000a00 00100102 02010600 00000000 00004010
00000000 20000000 00100900 000808c0 08300000 50019800
060080a6 00201000 00200000 000c0008 00000000 02000080
00800000 00100201 00000100 18020320 01000080 00500014
00002001 80000000 00040000 10000001 00000000 0006800a
c0000000 00822600

>D00113
00000040 01000000 00100100 00010700 00080040 00000010
00000000 20000040 00000000 010808c0 08200000 50019000

```
11008002  00880000  40040000  00081008  08a20000  02000000
05c02000  00000201  00010020  18000a10  01000080  00500010
00000401  80000000  00000000  10001000  00000200  00060006
00022000  00002600

>D00110
00010000  01000800  00100100  00010700  000c0050  00204010
00000000  20000000  00000100  010008c0  00200000  50019000
1c00801a  01890000  00000000  02081008  00a20000  02000000
01c02010  00000201  00000020  18020a30  01000080  00500014
00001401  80002000  00088000  10001000  00000200  0006000e
c0020000  02002200

>D00106
00010040  81040000  00000800  80010101  00280041  00000000
00000000  28000402  00000000  01108048  08000200  40009000
84028000  02800000  02080404  01081008  00800004  00000000
11406010  00002001  00000020  00000a00  01008000  00000010
00201400  80000400  00404200  80000000  00004240  01000000
24020000  00000000

>D00095
00000000  4a000008  20100100  00010600  00000080  80004000
02000000  00000000  00000100  00080840  08200000  40008000
00000002  00000000  00000000  00088008  00000000  02000000
00000000  00020200  00000000  18200020  02000000  81400010
00000000  80000000  00520000  00000000  04000000  00060002
40000000  00000600

>D00094
00000400  01040020  00000000  00000000  40000000  00000001
00000000  00000402  01004000  00100000  00000000  40000000
00028000  20020004  00000400  0008000c  00000000  00000020
02092090  00000001  00000000  00000a00  00408000  00000012
00201000  00000000  00404000  80000000  00000000  00000000
24820000  00000000

>D00079
00050000  01000000  00000800  00000000  00080000  00000000
00000000  28000402  00000000  00108008  00000200  40001000
14028008  00800000  00000404  00080008  00000000  00000000
00402010  02000001  00000000  00000a00  01008000  00000010
00201000  00000400  00404200  80000000  00000000  81000000
24020000  00000000
```

>D00059
00000000 42000008 20000100 00010600 00000000 c0000000
02000000 00000000 00000800 00080840 08100000 4000b000
04008002 00000000 00000000 00088008 00000000 02000000
00400008 00020201 00000100 18000a00 03000040 81400010
00000800 80000000 00120000 00000000 04000000 00060002
00000000 00000400

>D00049
00000002 00800800 00004100 00000210 00041000 00004000
00000000 00000000 00000100 00000040 00000004 40000000
00008800 00000000 00000000 000c000c 00000000 02000004
00200000 00000000 c0000000 08048a40 20800000 0040000c
00040000 00000000 08000000 00000000 00400000 00020000
c0000000 00000000

>D00666
00008000 01000000 00100100 00010700 00080010 00004010
00000000 01000400 01000100 000808c0 08200000 40018800
020080a6 00890000 00000000 02081008 00a00000 02000000
00e12010 00000201 00000020 18000b30 01000080 00500010
00003401 a0003000 004c0000 50000000 00000280 00060002
44000000 02022600

>D00665
0000800a 00000200 00104100 10010600 00040020 00080000
00000020 01001400 01000800 000808c0 08308004 40808800
02008824 00000000 00020000 020c008c 00000000 06000001
00210018 00000001 60000100 18008b00 20800000 00400010
00003000 a0001000 00440040 50000100 00000084 0086000a
04000000 20020600

>D00646
00000000 00000208 20100100 00010600 00000001 00000000
02000000 21000200 00000800 000808c0 08300000 40008240
00000002 00000000 00000000 000c2008 00000000 02000000
00000000 00020201 00000100 18000000 01000000 00400010
00000000 a0000000 00020000 50000000 00000080 0006800a
02000000 00000600

>D00645
00004400 00809000 00000100 00d10600 00000000 00000000
00000000 02001200 00000000 00080840 00202000 60008000

00000812 00000002 c0200600 00000000 00000000 02000000
00000000 00002000 80000000 08008000 00000000 08400008
00000000 c0000000 00020000 c0000020 02000100 00060000
22040008 08010220

>D00635
04040000 00000008 20100108 00010600 002010c0 02000000
02040000 00000040 00000000 00080840 08200000 40009000
04808002 00000000 01240001 00082008 18200000 02000000
04c00000 00020201 00000000 18000a00 07000080 08400010
00000001 80000000 00120000 00800000 00000202 00060002
00000000 00000640

>D00632
00000000 43000208 20100102 00010600 00000000 80000000
02000000 20000000 00000880 000808c0 08300000 40008000
00000002 00200000 00000000 00048008 00000000 02000000
00000000 00020201 00000100 18200000 00000000 81400010
00000000 80000000 00120000 10000000 04000000 0006000a
00000000 00022600

>D00627
0800e00a 00800a01 00101500 10050610 000c0061 0000c000
00000000 00081000 80500100 100018d0 0110800c 40818800
82009880 00608000 200300c0 020c000c 00020000 06802004
04680008 50410005 c0100008 38028a30 31880041 81500414
00040000 c0000000 00000040 00000040 0440400c 00c60c08
c00c0804 200004a0

>D00613
00008000 46000008 20110100 00510600 00001020 80000000
16000400 21000201 00000080 00080844 08202000 40808900
02000006 00200002 80000000 00008018 00000200 02100000
00100400 80020200 00000400 18200110 00004000 815a0010
00002000 e0000a00 00120000 40000000 06000080 00060002
20000000 00032620

>D00609
00010082 00202809 20080108 0c080600 06041008 00004000
0608a000 02201808 02004100 20010840 01200034 40009000
4400cc16 00402008 01000461 001c800c 00004000 02004008
00600000 00a60400 40000000 2e048628 20800860 0268020c
00802020 60000000 04140010 88000040 08000398 00460809
c0001000 002a02c0

>D00607
00000040 01000000 00100102 80610d00 00080080 00000004
00001800 00002800 00200200 00000022 00202000 40008000
00001200 00880002 00001008 00389008 20a00040 00000000
02c02000 00000001 00000020 10012000 17000080 08000010
10000402 00000000 40000000 24000020 00000212 00040000
00400000 00010220

>D00606
00008000 00000000 00110100 00010e00 00000000 00000020
00000000 00002000 00200200 00080842 08200000 40009900
03009004 00000400 40000000 00100000 00008008 02000000
00000000 00000000 00010400 18210700 00000000 81400010
00002000 80000200 40000000 00000000 04000010 00060003
00002000 00020600

>D00604
00008042 00000000 00000000 00000e80 00000000 00000020
00000000 00002000 00000400 00000842 10200004 44008820
02000004 40000408 000000c0 00100080 00000800 00002000
01000000 00000000 40000100 28010100 22000000 00000000
00082008 08000400 40000000 00000800 08000010 00860080
00000000 00020200

>D00601
04040000 00000008 20100108 00010600 002010c0 02000000
02040000 00000040 00000000 00080840 08200000 4000d000
14808002 00000000 01240001 00082008 18200000 02000000
05c00000 00020201 00000000 18000a00 07000080 08400010
00000001 80000000 00120000 00800000 00000202 00060002
00000000 00000640

>D00598
04000040 00000008 20100108 00010700 000010c0 02000000
02040000 00000040 00000000 00080840 08200000 40008000
00800002 01000000 01240001 00083008 18200000 02000000
04c00000 00020201 00010020 18000000 07000080 08400010
00000401 80000000 00120000 00800000 00000200 00060002
00000000 00020640

>D00574
00010002 01001800 00100102 40010600 00000000 00000000
00000000 20000004 00100880 000a08c0 08300004 50019000

0500801a 00200000 40000040 00000000 00000000 02000000
00002000 00000201 40020100 18240610 20000000 81500018
00000000 80000000 00000000 10000000 04000000 00060003
00002000 00402600

>D00563
0800400a 00004002 40104120 14010618 00041020 04082040
11002401 23001041 00500180 38090a40 08200004 4080c000
01000ca2 00202000 00020400 021c0086 04259200 02004005
08300400 00a00200 45120008 3e208400 21814821 83740012
00040080 e0000080 08004088 c0001080 0e488198 00860803
200610a0 20010600

>D00560
00000002 01000400 00100102 02010600 000c0040 00004090
00000000 21000000 04300900 800809e0 0d30800c 50018000
00009002 01e41000 003000c0 000c0004 00100000 02000080
00600000 00100201 c0000100 18000030 21800001 00700010
00000000 e0000000 00000000 50000001 00000080 00468012
40080000 00002680

>D00559
00030002 21000300 00100882 03800414 09000120 00000000
034021a0 28028009 00400880 004d0080 00308a00 50802000
0000000a 00201801 02422000 020c4004 00000200 06000080
02006018 80204201 20100800 10a80080 24004800 70010010
02047000 0c000401 00200000 10000184 0402000c 04062008
18000000 20032210

>D00542
00100000 00008000 00100000 00000000 00000000 00000000
00000000 00000000 00000000 00000000 00000000 40000000
00080100 00000000 00000000 00000000 00000000 00000000
00000000 00000000 00001000 00000100 00000000 00000000
00000000 08000000 00000000 90000000 00000000 00000000
00000008 00020200

>D00540
00010040 01000000 00100302 80010700 000800c0 00004010
00000000 00000040 00000900 000808c0 08300000 4000d000
0500800a 00a80000 40041008 00099008 18a60000 02000000
04c02000 00000001 00010120 18000a20 17000080 08400010
10000400 80000000 00000000 10000021 00000202 01060006
40022008 00000600

>D00528
0000002a 00801800 00080000 02010700 00080040 0000c000
00008000 00000000 06400100 800000a0 0c00800c 00000000
00009000 00400000 00280080 000c000c 00800000 00088000
00400000 00000000 80000100 10000040 21800041 00300004
00040000 c0200000 60000000 00040000 00004000 00460000
c0000000 00000280

>D00525
00050042 01000008 00001102 00810710 04080140 00000000
01002000 00000100 00400900 00080000 20008008 40019800
04009000 00100800 000a0000 000c100c 00820000 06000000
00402008 40000001 80100420 10000a00 210000c0 00000410
00040401 42000200 00000000 00000080 00000206 00460002
40000004 20c00c00

>D00524
00000000 00400000 00000200 00000600 00000080 00000000
00000000 00000000 00000000 00000000 00200000 40008000
00008000 00000200 00001000 00180008 00060000 00000000
00000000 00000000 00000000 00000c00 12000000 00000000
00000000 00080002 00000000 00000000 00000200 00040000
00000000 00020200

>D00523
00012042 01801019 40300140 04090e01 00000100 08020008
10010004 22087000 00000300 20010cd3 10218010 4001b000
84108c8a 00202008 20000400 0018200c 00060802 06002800
0411a000 10020201 80270101 38058690 90008011 02504818
00000080 c0220000 50410000 94000040 0a004114 01068001
a0040200 28002200

>D00522
00002002 01801a19 40000104 00050610 800c0050 0802c108
00000004 0008d004 00500300 000008c0 0104800c 40018000
800098a0 00600000 600003c0 021c200c 10020100 82000804
04600000 50040000 c0060140 18068a70 31808041 8140400c
00240000 80200000 50000040 10002040 05004204 00460008
c00c0200 0c2004a0

>D00521
00000000 01000000 00100102 00050600 00100000 00004000
00000000 21000000 00100900 000808c0 08300000 50018800

00000002 00200000 00000000 00080008 00000000 02000000
00400000 00000201 00000100 18000020 01000000 00500010
00000000 a0000000 00000000 50000000 00000080 00060002
40000000 00002e00

>D00520
0000802a 04801800 00080100 82010600 000800d0 0000c000
00008000 01000000 06400100 800808e0 0c20900c 40809000
03009008 00410000 c0281088 020c000c 00800000 02008000
00400000 00002000 80000100 18000160 33800041 00700014
10040400 e0202000 60080000 40040000 00004280 00460002
c0002000 00020680

>D00514
08000002 00000000 00000100 10010610 04000041 00080000
00002000 05001000 00400100 00080840 28008000 40008a00
00000000 00300000 00020000 000c000c 00010000 02000001
00000000 40000000 00000000 18008400 21004000 00400010
00040000 80000200 00000000 40000000 02000084 00420003
40000000 20010400

>D00513
04000002 00000008 20100108 00010600 00041080 02000000
06080000 00000008 00000000 00000840 00200004 40008000
00800806 00400000 01200041 000c200c 10200000 00000004
00a00000 00060200 40000000 18008008 27800080 88000010
00002001 00000000 00000000 00800000 00000200 00060000
00040200 00200240

>D00509
00004002 80000808 20224120 04d10601 00000100 00000040
12000010 00024200 00002000 00000a43 10222014 40008000
00002002 0000a00a 80000040 00100008 000408a2 06002000
08000002 00022400 40300100 28010000 a0000002 02400002
00020080 80000000 42000000 00000080 08008010 00068000
00800000 20010220

>D00503
0020c002 00820600 00120000 05100600 20040080 08002000
08010010 00000000 00022000 00400d60 00244004 61008800
02000006 00002001 20120040 0208000a 14841000 20000000
08200002 02024200 e2100000 38000900 22800021 02180019
00006401 10000100 12204010 00000080 08008200 01060010
0000a000 00030220

```
>D00499
00010000  01001000  00100000  00000c00  00000000  00000000
00000004  00002000  00000100  00000180  00200000  40011000
0d009008  00000000  00000000  00000000  00000000  00000001
00002010  00000001  00020100  100d0610  00000000  00100018
00012000  00000000  00000000  10000000  00000000  00040211
000a0000  00000200

>D00498
00000000  63008208  20100102  00010600  00000020  40000002
02100000  20000401  00000880  000808c0  08300000  50818000
00000002  00210000  00000020  020c0008  20000200  02000100
02002000  00020201  00000100  18200010  00004000  81500010
00000000  80000001  005a0000  10000000  04000002  0006000a
04020000  02002600

>D00494
00204002  00820200  00120020  00100600  20040000  00002000
00010010  00000000  00022000  00400e40  00244004  61008000
00000000  00002000  20020040  02000002  04841000  20000000
08200002  02020000  c2100000  38000000  20800000  0018001b
00004401  10000300  02204010  00000080  08008200  01060000
0000a000  00010220

>D00493
0020c002  00820600  00120000  05100600  20040000  08002000
08010010  00000000  00022000  00400d60  00244004  61008800
02000006  00002001  20020040  02000002  04841000  20000000
08200002  02024200  e2100000  38000900  20800021  02180019
00006401  10000100  12204010  00000080  08008200  01060010
0000a000  00030220

>D00483
04000000  00000008  20100108  00010600  00001080  02000000
02000000  00000000  00000000  00000840  00200000  40008000
00800002  00000000  01200001  00082008  10200000  00000000
00800000  00020200  00000000  18000008  07000080  08000010
00000001  00000000  00000000  00800000  00000200  00060000
00000000  00020240

>D00480
00204002  00820200  00120020  00100600  20040000  00002000
00010010  00000000  00022000  00400e40  00244004  61008000
```

00000000 00002000 20020040 02000002 04841000 20000000
08200002 02020000 c2100000 38000000 20800000 0018001b
00004401 10000300 02204010 00000080 08008200 01060000
0000a000 00030220

>D00465
00010200 01000000 00420102 00010700 00482040 00004000
00000000 00100060 00000980 00080840 08300400 4000d000
0500800a 00a00000 44160000 80091008 08920000 02000000
06402000 00080001 00010120 08000a20 05090000 08400010
00000400 80000100 00000000 00000028 00000202 00060002
40022004 00000602

>D00454
08210246 43842800 02100904 0c200600 00120003 80100004
10c80190 10301054 10016600 80030843 12202204 440584b0
0000081a c010a809 040634c0 18180088 08658809 1308401a
09000002 30809281 40022000 78029080 e7104120 02401000
03088000 4c000180 4a800410 9d011c84 08988918 01ae0000
20000081 50e10201

>D00451
00000002 04000820 00102100 41310610 00041000 00000000
00002004 00008000 00400100 00090844 0820240c 40008800
10001820 00440001 80000040 0004000c 00000018 02000104
00200400 00004000 40000000 18a08008 20800000 91400030
00042000 80000000 08000040 0000000c 04400000 00060002
000c0200 00010660

>D00443
00050040 01000120 00010100 00110304 00080002 00000000
00000081 00000402 05a04000 0010c001 00000000 40029100
04038008 00840004 00080400 00081008 04820000 000000a0
02412000 00100801 40000020 00000a80 01008300 00200010
00200400 00000000 04404000 80000000 00000200 01001822
24020000 81010000

>D00442
00010002 81007a10 60100100 45910710 00041280 00000100
00002000 2000000c 00400880 020f08c0 08300004 4001b000
1e00880e 00442801 80180040 000c000c 10000000 02000000
01e02408 004c2301 40000140 38248638 23800683 97500018
00043801 80800000 08000400 10000000 0c000000 0006802b

20044200 03412620

>D00440
00420002 40000010 22000800 10100c00 00000004 00100006
010400a4 0001a409 14000000 02014000 00210a00 52080080
00010494 00040021 00022000 00040114 04001080 222083e0
00200010 01003043 00001502 80010080 20000200 08810000
02141000 0c100200 00204000 000a1324 00000204 04061000
00002004 02112200

>D00437
00410002 81000019 20200100 80030700 00080040 00900000
02002821 00000400 15400c00 009a0860 4e300004 40019000
0c03e0a2 02002000 20120140 10098159 00830008 03100000
00430018 06006801 40020101 08100a00 31048040 00c00890
84803800 c0000000 00500000 90040101 00100200 80060203
04200000 64000610

>D00432
04000000 63000008 2010050a 00010600 000010c1 c2000000
02001000 00008201 00000810 00080840 08300000 40008140
00800002 20200080 01240001 00082008 18200200 22008000
02c82000 00020201 00000100 18000000 070040c0 08400410
00000001 c0000001 00000000 00800000 00000302 00074802
00000000 00000640

>D00426
0000008a 01002a00 001a1100 33854618 000c0222 00104050
01003820 20008410 06420980 900918f0 2830840c 50818800
00009082 02f09040 00020080 200c0804 01008200 86838002
00600008 40800201 84a02104 18210018 21c04041 10780594
00040000 c0010008 20000030 18041185 84204005 01c7820a
40280084 e00026b8

>D00422
00400082 40200010 20082508 00100e00 0c000000 00000000
00000024 02343400 14004100 02004000 01200020 40081080
c4011c04 40040008 00000020 00000110 040040a0 22008d00
80000000 08003142 00001500 08018000 00000201 08480000
00840020 00000200 04104010 88080120 00000100 0006c000
10082004 02010200

>D01024
14002010 00800008 21102108 00b10602 00001180 00010000

```
02040000  00000000  00800880  00080840  08302000  6000c000
00804002  00000001  81281081  02088108  11000201  02000000
00000000  00022201  80000100  18240000  12000000  89400018
00008000  80000000  00120000  01000009  04000200  2006000a
00000000  000306a9

>D01022
00000004  42000808  20300100  00010601  00000121  80000000
12000000  00004200  00000180  20080843  18210010  40008800
00100042  00200008  00000000  00182008  00040a02  06002000
00000000  00220200  00200100  38210000  c0004801  83400810
00000080  80000000  40020000  00000800  0c000010  00068002
40000000  20020600

>D01020
00000000  4a000008  20000108  00010600  00001000  c0004000
02000000  00000a00  00000900  00080840  08100000  400080c0
00000002  20000000  01000005  00082008  10000000  02000000
00080000  00020201  00000100  18000020  03000000  81400410
00000000  c0000000  00080000  00000000  04000300  00060002
40004000  02020400

>D00996
00000000  4a000008  20100100  00010600  00000080  80004000
02000000  00000000  00000100  00080840  08200000  40008000
00000002  00000000  00000000  00088008  00000000  02000000
00000000  00020200  00000000  18200020  02000000  81400010
00000000  80000000  00520000  00000000  04000000  00060002
40000000  00020600

>D00987
00010002  21005c00  00100102  41810e10  00041100  00000000
00002004  20002401  00510980  000909e0  08300004  50019000
9c00981a  00610800  00000040  000e000c  00000200  02000104
00202450  00000201  40020100  183d8638  21800000  91500018
00053000  80000001  08080040  10800000  04400002  00060013
041e0200  02402620

>D00965
00014006  01003810  40100120  00010e00  24000000  80000000
00000c04  00002009  14000000  00000a70  04280004  4000b004
04019080  09102000  20120140  00000010  00040000  02402000
08000000  00000011  48120100  38050600  20022000  0040001a
00140000  c0000000  00000000  900000a0  0a408000  00060001
```

20104000 04004200

>D00954
00010000 01000020 00000000 00000000 00482000 00000000
00000080 00000402 01004000 00108000 00000080 40001000
04028008 00800004 00000400 00090008 00000000 00000020
02412000 00000001 00000008 00000280 01008000 00000010
00200000 00000000 00404000 80000000 00000000 01000200
24020800 00000000

>D00845
00054002 01005400 00101100 00010700 20380080 00000000
00000080 20000000 00000080 004a09e0 002c0204 d001d080
1400a032 02100000 020a3040 00084008 10040000 02000008
09404000 00000205 60100800 58280e80 37002000 81500018
02002000 04000000 08800200 10000085 04008202 00060013
10000000 00c12e00

>D00838
00000000 63000208 20000102 00010600 00000020 c0000002
02000000 00000001 00000800 000808c0 08100000 40818000
00000002 00200000 00000020 02080008 00000200 02000000
02002000 00020201 00000100 18000010 00004000 81400010
00000000 80000001 00120000 00000000 04000002 0006000a
00020000 00000400

>D00837
00040000 63000208 20100502 00010600 003800e1 40000002
02001000 20000001 00000880 000808c0 08300000 5081d000
14008002 00a00010 000c0020 020c0008 18000200 02000000
03402000 00020201 00000100 18200a10 07004040 81500010
00000000 80000001 00120000 10000000 04000002 0006080a
00020000 00002e00

>D00790
00008000 0c800200 00120300 00110600 00062001 08100420
84480080 01004402 04000080 00180844 08200400 60008800
0a000004 00000011 82000002 00004000 04003400 02000044
00200010 00000000 80000000 18000900 00808020 00400019
01002000 a4000000 10402400 40001000 00400290 00070f12
04001000 00030620

>D00780
02014052 29d0fc00 01900302 c5d10710 000c1180 00038010

```
c08a38c0  a420060b  00c10bc0  001909ea  08302004  5101f600
be02893e  01612b03  c05c124a  583e124c  30800640  82090544
00662d16  00004201  40800530  3a3486f8  33808063  9350003c
31243400  80081803  18da284c  f0200021  0c404200  00260c13
a4044208  1a012620

>D00779
00000000  81000220  00100102  00010600  00080000  00004000
00000000  20000402  00004900  001808c8  08300000  50018000
00020002  00a00004  00000400  000c0008  00080000  02000020
02c02000  02002201  00000100  18000030  01008080  00500010
00200001  80202000  00404000  90000000  00000022  0106000a
64020000  00002600

>D00775
00030002  10000008  20100008  00000200  000c1010  00808000
02080100  00028018  00400000  01001840  00000a04  10408000
00000026  02400000  010020c1  0004040c  00000000  00000080
00200010  00220000  40000000  4c000082  20800808  02210000
00002000  0c000000  40000000  c0000804  02000208  000e2000
00000000  00410600

>D00769
00018008  00000000  02100904  10410600  201000e2  00000000
00000080  02001840  00600080  00480850  08202200  d001ba80
0300a8de  00200003  c20c3400  00084008  18022201  02000028
04004000  00008205  00020c10  58289b80  33004100  81400010
0200a000  c4000200  08014000  90000105  04000300  00060002
38046000  00c30621

>D00765
00000000  01000000  00100300  80010700  00080080  00000010
00000000  20000400  00000000  00000080  00200000  c0018000
00008002  01810000  00281008  00081108  10860000  00000100
02402000  00000201  00000020  10000a10  13000000  00100010
00000400  00000000  00080000  10000001  00000200  01040002
04020008  02002200

>D00760
00040049  46000008  22100108  10010700  20201020  c0000102
42040000  02001601  00400880  00180850  08300000  50819040
0400c8c2  20280000  01000401  0008b008  40220200  02000124
10c80000  00020a01  00020120  18308a00  21404080  81448410
00000401  c0000001  08324000  90000000  04000302  0006000e
```

24040000 00022700

>D00726
00008002 88002808 20000108 84010600 00001000 00020100
06000000 02001408 00000000 00110848 08200004 40008800
02008806 08002000 81000441 00002008 00000000 02000400
00000000 00020400 40000000 28028700 20000000 0240000c
00002000 c0000400 00100800 80000000 08000300 00060801
84040000 00020200

>D00715
00000040 01000000 00100100 80010700 002800c0 00000010
00000000 20000040 00000000 010808c0 08200000 50019000
11008002 02880000 400c1008 00081108 18a20000 02000000
15c02000 00000201 00010020 18000a10 13000080 00500010
10000401 80000000 00000000 10001001 00000200 00060006
00022008 00002600

>D00688
00000001 4a000008 20100100 00410600 00000080 80004000
02000000 00000800 00000180 00080840 08202000 40008000
00000002 00000002 00000000 00080008 10000000 02000000
00000000 00020a00 00000000 18200020 02000000 81400010
00000000 80000000 00000000 00000000 04000000 00060002
40000000 00010620

>D00687
00000040 01000808 20100102 00010700 00081081 00024000
02000000 20000800 00000980 000808c0 a8300000 40018000
00008002 00a00000 00080000 00083008 10800000 02000000
00402000 40020201 00000120 18220a38 03000000 81500014
00000400 80000000 00520000 10000000 04000200 00060002
c0000000 00002600

>D00684
04000000 4a000808 20100108 40750600 00001080 80004000
02040000 00000200 00000180 00080840 08202000 40008000
0080c202 20010082 81010005 02088008 10020000 02000000
00480000 00020200 00000000 18220a20 12000000 81c01014
02000000 80002000 00520000 00000004 04000200 0006000a
c0000000 10010620

>D00683
00000000 00000008 20100100 00410600 00001081 00004000

02000000 00000800 00000180 00080840 a8202000 40008000
00000002 00000002 00000000 00082008 10000000 02000000
00000000 40020200 00000000 18200020 02000000 81400010
00000000 80000000 00520000 00000000 04000000 00060002
40000000 00010620

>D00682
00010000 81000000 00000800 00000400 00280000 00000000
00000000 28000402 00000000 00108008 00000200 40001000
04028000 00800000 00000404 00080008 00000000 00000000
01402010 00002001 00000000 00000a00 03008000 00000010
00201000 00000400 00404200 80000000 00000000 01040000
24020000 00000000

>D00676
00040002 04000820 00102100 41310610 00241000 00000000
00002004 00008000 00400100 00090844 0820240c 40009800
14009820 00440001 80000040 000c000c 00000018 02000104
00600400 00004001 40000000 18a08a08 21800000 91400030
00042000 80000000 08000040 0000000c 04400002 00060002
000c0200 00010660

>D00675
00000006 00808808 40100100 41010610 00041040 0000c004
0000a000 00001000 00400100 00090840 0820000c 40008800
90008800 00400000 00040040 000c000c 08008000 02000004
00600400 00200000 c0000000 18228a68 21800040 91400014
00042000 80000000 08008040 00000000 04400000 00060002
c0040200 00000720

>D00674
00000002 05004a00 00100100 40b10610 00041201 00000000
00022000 20000000 00410900 010908c0 08302404 50018800
08000802 00440801 80000040 00040004 00000000 02008004
00200400 00014201 40000100 98008008 20800000 82540030
00042000 80000000 08010040 1000000c 00400000 0006000a
00040200 01030660

>D00673
00400082 40200010 20082508 00100e00 0c000000 00000000
00000024 02343400 14004100 02004000 01200020 40081080
c4011c04 40040008 00000020 00000110 040040a0 22008d00
80000000 08003142 00001500 08018000 00000201 08480000
00840020 00000200 04104010 88080120 00000100 0006c000

10082004 02030200

>D02356
00000000 43000008 2010010a 00010600 00001000 c0000000
02040000 20000000 00100880 000808c0 08300000 40018000
00004002 20200000 01000001 0004800c 00400000 02000000
00082000 00020201 00000100 38200010 00800400 81500010
00000000 80000000 00120000 10000000 04000200 0006000a
04000000 00002680

>D02354
00205002 00c20600 00120000 06100600 20040000 00022000
00011010 00000000 00022000 00404d60 00644004 61008020
00000002 00042100 20020140 02000002 04841000 20000000
08200002 02020200 e2100000 38000000 20800021 02180019
00004401 10000100 02204810 00000080 08008200 01060010
1100a000 00010220

>D02349
04000000 4a000008 20100108 00210600 00001080 82004000
02040000 00000000 00000100 00080840 08200000 40009000
0080c202 20000080 01010005 0208a008 00020000 02000000
00080000 00020200 00000000 18200a20 13000000 81400010
02000000 80000000 00520000 00000004 04000200 0006000a
40000000 10000640

>D02342
04000000 00000008 a0100108 00010600 00001090 02004000
02040000 00000040 00002100 00000840 00200000 40008000
00800002 00010000 01200001 02082008 10200000 02000000
00800000 00020200 00000000 18000020 07000080 08400010
00000401 80002000 004a0000 00800020 00000200 00060000
40000000 00000240

>D02340
00a48076 08060028 2008010c 8c010600 80403000 40000130
1600c014 02401400 00410504 4111084b 18210004 c4008830
02000816 4020b00a 810005c3 00186888 02030800 0e020400
0100a000 00020000 61440100 2c00810c 27000220 0a420000
01087020 c8000004 60100500 802108a0 0908033a 10860460
44160c00 b0060240

>D02338
04004002 00000808 60100108 00010600 24001080 82022000

```
02000000  00000208  00400010  000808d0  08280004  40009020
04808022  00100000  01220041  10082008  10240000  22008000
08800000  00020201  44100000  18020600  27002080  08400014
00040001  c0000000  00040800  10800080  20408300  00060003
80000000  00000640

>D02327
0004000a  00000000  02004100  90010600  002400b0  00084000
00000000  01001000  00000300  00080840  08209004  40809000
04008800  00010000  00021008  020c008c  00008000  02800001
00600000  00400001  40000000  08008a20  b3802002  00400110
00000400  a0002000  00080040  40000000  00000286  00060402
40000000  20000600

>D02279
00010000  01000000  00000000  00000400  00080000  00000000
00000000  00000000  00000000  00000080  00000000  40013000
04008008  00800000  00000000  00080008  00000000  00000000
00402000  00000001  00000000  10000a30  03000000  00000010
00000000  00000000  00000000  00000000  00000000  00040000
00000000  00020000

>D02278
00010000  01000000  00000000  00000400  00080000  00000000
00000000  00000000  00000000  00000080  00000000  40013000
04008008  00800000  00000000  00080008  00000000  00000000
00402000  00000001  00000000  10000a30  03000000  00000010
00000000  00000000  00000000  00000000  00000000  00040000
00000000  00000000

>D02250
00010002  81007a10  60100100  45910710  00041280  00000100
00002000  2000000c  00400880  020f08c0  08300004  4001b000
1e00880e  00442801  80180040  000c000c  10000000  02000000
01e02408  004c2301  40000140  38248e38  23800683  97500018
00043801  80800000  08000400  10000000  0c000000  0006802b
20044200  03412620

>D02237
00000000  01000000  00100000  04400c00  00000000  00000000
00000000  20002800  00000400  00000082  00202000  40010000
00002002  00002002  00000000  00100000  00010010  00000000
00002000  00000201  00000000  30010010  00000001  02100010
00000000  00000000  40000000  10000000  08000010  00040000
```

00020000 00012220

>D02234
01084200 09100100 00100300 00010600 22000000 00006002
80080480 01000440 04200000 001808e8 08200180 50018008
00a00002 00200000 00020023 00002002 04000400 02000040
00100000 00000201 00100000 18400000 0048c020 00500810
01000000 a0002000 00406000 50000000 02424080 00060c02
04000000 00820600

>D02150
00000000 4a000008 20100100 00010600 00000080 80004000
02000000 00000000 00000180 00080840 08200000 40008000
00000002 00000000 00000000 00088008 10000000 02000000
00000000 00020200 00000000 18200020 02000000 81400010
00000000 80000000 00520000 00000000 04000000 00060002
40000000 00020600

>D02149
00040000 4a000008 20100100 00010600 00200080 80004000
02000000 00000000 00000100 00080840 08200000 4000d000
14008002 00000000 00000000 00088008 00000000 02000000
01400000 00020201 00000000 18200a20 07000000 81400010
00000000 80000000 00520000 00000000 04000002 00060002
40000000 00000600

>D02147
00000000 00000008 20100100 00010600 00001081 00004000
02000000 00000800 00000180 00080840 a8200000 40008000
00000002 00000000 00000000 00082008 10000000 02000000
00000000 40020200 00000000 18200020 02000000 81400010
00000000 80000000 00520000 00000000 04000000 00060002
40000000 00000600

>D02082
0001014a 01800a00 08101500 52550610 00080045 04904000
810002a3 08001700 81800904 18f838d0 0030a208 c0009880
8c009890 00100002 80033082 000c004c 10008002 0e300008
00490018 40010807 80000900 580a8aa0 21208040 81410414
03441800 c6000000 80400081 00009684 84010004 80c6080a
cc0c0084 20c10432

>D02076
0004c006 04808010 00000000 00000e01 00240140 00000000

00028404 01006001 00020000 01020842 10200014 4000d000
1400a814 00400008 808400c0 001c000c 08040802 04402008
09600000 00000005 c0220100 28058a00 a5820000 00080030
00102080 00000000 40068080 00000080 0a008092 000e0000
00048108 2d004240

>D02070
00000040 01000000 00100100 00010700 00080040 00004010
00000000 20000040 00000100 000808c0 08200000 5001d000
01008002 00880000 40040000 00089008 08a20000 02000000
04c02000 00000201 00010020 18000a30 05000080 08500010
00000401 80000000 00000000 10001020 00000200 00060006
40022008 00002600

>D02066
00000000 4a000008 20100100 00410600 00000080 80004000
02000000 00000800 00000180 00080840 08202000 40008000
00000002 00000002 00000000 00088008 10000000 02000000
00000000 00020200 00000000 18200020 02000000 81400010
00000000 80000000 00520000 00000000 04000000 00060002
40000000 00010620

>D02007
04070002 01003000 00100000 05800410 04200240 00000010
00002020 2000000c 00400980 000b0280 20208000 50017800
16008012 00102801 81020000 000c000c 00000000 0e000000
01400018 40000201 20000c00 30240e20 25000003 12100018
00041000 00000200 00000000 10000100 0c000006 00460009
60004000 20002200

>D01994
0405800a 89005c08 20100108 82010600 00281080 04020110
06000000 02001400 00000000 00120968 08200004 4000d820
16808826 0a001000 81081449 00082108 10000000 02008500
01400000 00120601 40000040 180a8f00 37000000 0040001c
20002400 c0000400 00900800 80000001 00000302 00068811
a4040000 00020200

>D01973
00000002 05804a00 00120102 41910610 00041100 40000810
00002000 00000000 00410980 010948c0 08702004 60018800
18010802 00640800 82200040 00040004 04000000 02008004
00202400 00002201 c0000100 18208018 20800000 9b540019
00042000 80000000 08000040 14000000 04400002 00060002

```
10040208  01012620

>D01965
16006022  00000808  20100108  40210602  20001081  00010050
02002000  01001000  00800880  20080ac0  08300004  40018000
0080c802  00008000  010a1141  0a08800a  10048000  82000000
08800400  00820201  40100100  18268600  33000080  81400015
00040001  80000000  08000000  90801081  04088200  0006000a
80040000  60002680

>D01964
04010000  c1000008  20100908  00010300  00281000  02000000
02200400  28000402  00000000  00108858  00000300  40009008
04828002  01800080  01200405  00083008  00820000  02000000
01482010  0002a201  00000020  08000a00  0500a000  08400010
00201400  80000400  00404200  90800000  00000200  01020000
24120000  00000200

>D01925
04030002  01003000  00100000  05800c10  04000240  00000010
00002024  2000200c  00600980  000b03a0  20208000  50033800
0f00b012  00102801  81020000  000c0004  00000010  0e000001
00000058  40000201  20020d00  302d0620  21000003  12100018
00053000  00000200  00000000  10000100  0c000006  00460019
60084000  20802200

>D01891
04050000  81000028  20000808  80010300  00081000  02000000
02000000  28000402  00004000  00108848  00000300  40009000
1482800a  00800200  01200405  00082428  020a0000  00000000
00402010  02028001  00040000  08000a00  0500a000  08000010
00201000  00000400  04404240  80800000  00000220  81020000
24020000  00000000

>D01871
00000000  01800200  00120102  00110600  00060001  00100420
00400000  21004002  04100800  000808c0  08300400  70018000
08000002  00200010  02000002  00004000  04003000  02000004
00202010  00000201  80000100  18000810  00800020  00500019
00003000  a0000000  00000400  50001000  00000292  00070012
00021100  00032620

>D01828
00000002  00a40800  00084100  00010210  00041600  80014060
```

00000400 40000200 02084120 40080850 08000024 4000b002
40008c00 01600088 00020050 000c000c 00000000 42004004
04200080 00600000 c8000000 0e068a68 20a04800 8078001c
08040050 80000005 88000090 18140000 00402008 01860002
c0140000 80000400

>D01782
0000020a 00008000 00104100 10010600 00040020 00080010
80100080 29001408 00000100 004808c0 08208004 40808800
00000802 00010002 00020002 02040084 20000400 12000101
00200000 00800201 41000000 18008000 20800000 00410010
00000000 a2000000 00482040 50010200 00400084 00060002
04000000 22020600

>D01745
00018002 01000400 00100000 00000600 001c0040 00004080
00000000 20000000 04200100 800009e0 0d20800c 50018800
0200900e 01c40000 003200c0 000c0004 00100000 00000000
00600000 00000201 c0000000 18000130 21800001 00300010
00006000 40000000 00000000 10000000 00000000 00460010
40080000 00422280

>D01717
00020000 01000200 00100102 02010600 00100040 00000010
00000000 20000000 00100800 000808c0 08300000 50019000
05008002 00201000 40040000 000c000c 08400000 02000080
02402000 00100201 00000100 38000a10 01800400 00500010
00000000 80000000 00000000 10000001 00000002 0006800a
00022000 00822e80

>D01713
0800404a 00004012 40000120 10010e09 00000120 04082042
11030401 23007041 00100000 18080e52 19200014 4080c000
01002ca3 0020000c 00020040 02140082 04249802 06002003
08100000 00040040 45322108 38018000 a0014030 81540110
00100080 a0000000 40004008 d0001080 0e288190 00860003
200600a0 a4034600

>D01712
0000002a 00801800 00080000 02010600 00080040 0000c000
00008000 00000000 06400100 800000a0 0c00800c 40000000
00009000 00400000 00280080 000c000c 00800000 00008000
00400000 00000000 80000100 10000a40 21800041 00300004
00040000 c0200000 60000000 00040000 00004000 00460000

c0000000 00000a80

>D01699
04031000 41000208 2010010a 00010600 00001041 02000010
02040000 21000240 00300880 000808c0 08300008 40039000
0580c012 00210000 410c0001 100c2018 08000000 06000000
0c400000 00020201 00000100 18210620 01000000 81500010
00000400 a0000000 00120000 50800004 04000380 0006000a
40002008 00802600

>D01692
00010002 01001409 20100008 88090600 020c1088 00004000
0608a000 20100808 00000100 200009e0 01200004 4001d000
0400c026 01400000 01081049 080c800c 10000000 80004008
00600000 00a60601 40000020 1c080628 33800840 0030001c
10002400 00000000 00040000 10000041 00000218 00460819
40001000 002a22c0

>D01652
04808020 42c4460f 60301160 8d111680 c3884580 02e00807
14980000 20128403 94300b80 205f48db 48310130 4587d880
16139806 418c8008 048e9888 4808270f 1400e820 16c200c0
00440060 02360641 62000500 38832700 33008b23 00501818
10a12510 c0000684 40c04244 98021051 0400520c d086303b
c4080084 05138640

>D01603
00000000 00000000 00000100 00410e20 00000000 00000004
00200000 00003800 00000000 00000842 10202010 40008000
00000800 0000000a 00000000 00000000 00000800 02000000
00000000 00000000 00000100 28008000 00000000 00400800
00000008 80100000 40000000 00000000 02000010 00060000
00040000 00010020

>D01462
00000032 2910e800 02100302 45910e10 00041140 00000000
80082084 0400240b 01410980 015b084a 08300004 51018000
9802a902 00612800 00040042 400e014c 20000610 02000544
00232452 00801201 40800100 3a318e08 20808041 93400018
01243000 80001801 0848284c d0010100 0c404000 00260c03
2c040200 00012620

>D01454
04008000 40000008 20100108 00410600 00001080 02000000

```
02000000  00000000  00000000  00000840  00200000  40008800
02800006  00000080  01200001  00082008  10200000  02000000
02880000  00020200  00000000  18000100  07000080  08400010
00002001  80008800  00040000  00800000  00000200  00060000
00000000  00020240

>D01441
0001e202  00010800  80004100  44550610  09041004  04004000
00002800  02081200  00000100  00010840  00282004  6000d810
82008802  0010a002  80020440  800c0004  00060000  12006004
0c600000  10a00006  40100000  2c02a600  31812860  0240011c
000c0003  c0180000  08000600  800110e0  08c0810c  00460801
c0040800  200102a2

>D01390
00000000  4a000008  20100100  00010600  00000080  80004000
02000000  00000000  00000180  00080840  08200000  40008000
00000002  00000000  00000000  00088008  10000000  02000000
00000000  00020200  00000000  18200020  02000000  81400010
00000000  80000000  00520000  00000000  04000000  00060002
40000000  00020600

>D01386
00000000  00000000  00100100  00010600  00000080  00004000
00000000  00000000  00000900  00080840  08300000  40008000
00000000  00000000  00000000  00080008  00000000  02000000
00000000  00000001  00000100  18200020  03000000  81400010
00000000  80000000  00000000  00000000  04000000  00060002
40000000  00020600

>D01358
0800400a  00004002  40100120  14010608  00000020  04082040
11000401  23001041  00100080  18090a40  08200004  4080c000
01000ca2  00202000  00020440  02040082  04249200  02000001
08100000  00000200  45120008  38208000  20014021  83540012
00000080  e0000000  00004008  c0001080  0e088180  00860003
200600a0  20030600

>D01352
00010000  81000000  00000800  00000400  00280000  00000000
00000000  28000402  00000000  00108008  00000200  40001000
04028000  00800000  00000404  00080008  00000000  00000000
01402010  00002001  00000000  00000a00  03008000  00000010
00201000  00000400  00404200  80000000  00000000  01040000
```

24020000 00000000

>D01346
0000c002 00003a08 20100120 84090600 20000000 00006000
02000000 0200100c 00000980 00090ac0 08380004 4000b000
06008802 0010a000 00020440 000c0008 00040000 02000000
08000008 00020201 40100100 38228e20 20002000 8340001e
00000800 c0000000 00120000 90000080 0c008100 0006000b
e0044000 00000600

>D01332
14090040 09000800 00500b02 80010600 2a120004 0000a002
800804c0 01000401 05000800 005848c8 08300200 d0019280
0101801a 00200001 41003022 0008200a 05030400 82000048
00352010 00004205 00100900 5c980ac0 0940c000 10500814
03005000 a4001181 00413000 50800004 02c00082 00060c02
8c022020 00c14f10

>D01324
00204002 01820600 00120000 00100600 20040000 00002000
00010010 20000000 00022000 00400de0 00244004 71018000
00000002 00000000 20020040 02000002 04841000 20000000
08200002 02020201 e2100000 18000020 20800000 00180019
00004401 10000100 02204010 10000080 00008200 01060010
0000a000 00012220

>D01297
02000022 00800400 00104180 44000700 001c0040 00004060
00008000 00000000 06200100 c00008e0 0d20100c 4001a000
02009802 01e02001 003408c0 000c0004 08030000 0000c000
00600000 00000000 c0000000 38008678 21800063 82100000
08000000 c0000000 00008000 00040000 08000000 00460013
400c4000 00020e80

>D01295
00000000 00008000 00000100 04010600 00000000 00000000
80100080 03001400 00000000 00490840 08200000 40008000
00000802 00012000 00000402 00000000 20000400 02000100
00000000 00801000 00000000 28008000 00000020 02400010
00000000 e0000000 00482000 c0010000 08400180 00060002
04040000 00000600

>D01269
00040008 01c01800 02100300 90010700 203000a0 00008000

00000400 a3001005 00400280 000808d0 08202000 50819000
040088c2 00200300 00081480 081a0008 10060200 02080020
00500800 00000201 80020000 18208e10 33004000 8150001c
00000000 e0081002 08000200 d0200001 06000382 00060003
a0040000 00000e00

>D01242
0020c02a 04821a00 001a0020 02110600 200c0040 0000e000
00018010 00000000 06422100 80400ee0 0c24c00c 61809000
03009008 00402000 e02a00c0 020c000e 04841000 20008000
08600002 02022000 c2100100 38000140 21800041 0038001f
10044401 d0200300 62204010 00040080 0800c200 01460000
c000a000 000302a0

>D01236
0d00e202 00001803 00110100 d1010e10 24000060 0400a048
11002000 0208304c 00510180 18090a50 20298104 4080c800
03008802 00308001 a1020440 820e0006 04268200 1a102000
0c002000 40400200 44120808 18228600 31016020 9054001d
10841001 c1080280 40000210 800050c0 0409c104 03660c01
e00408a0 20022200

>D01227
04004016 80801018 6100050a 18b90600 8100102a 04004004
02809100 00000808 90104380 a0080840 08042004 60049840
02044482 0a000103 816a0005 023cc02c 04082000 1a004008
08402100 10266001 814a0200 1a241900 26c40044 08402018
00000a00 b0000200 10220000 0880002c 0400062c 2046000a
50004400 082505e0

>D01164
0400c002 80000208 22100128 05810700 30101000 06002040
0a040000 28000000 00400800 00080ad0 08380024 50019800
06808006 08102001 11020041 000c2008 00048080 12000000
08400008 00026601 40100100 38200700 21002021 03410016
00002800 81100400 00120210 10001080 88008200 0006000b
00000000 20020e00

>D01126
00010000 01000000 00100000 00000c00 00080000 00000000
00000000 00002000 00200200 00000082 00200000 40033000
04009010 01000000 00000000 00180008 00000000 00000000
00400000 00000001 00000000 10010a00 03000000 00100010
00000000 00000000 40000000 10000000 00000010 00040000

80080000 00020200

>D01118
00204002 00820200 00120020 04100600 20040000 00002000
00010010 00000000 00022000 00400e40 00244004 61008000
00000002 00002000 20020040 02000002 04841000 20000000
08200002 02020000 c2100000 38000000 20800000 0218001b
00004401 10000300 02204010 00000080 08008200 01060000
0000a000 00030220

>D01103
00000040 01000000 00100100 00010700 00080040 00004010
00000000 21000040 00000100 000808c0 08200000 5001d000
01008002 00880000 40040000 00089008 08a20000 02000000
04c02000 00000201 00010020 18000a30 05000080 08500010
00000401 a0000000 00000000 50001020 00000280 00060006
40022000 00022600

>D01071
00010000 09001020 00900000 00000c00 00000000 00000000
00080044 00002402 00004140 001001a0 00200000 41011000
0d029008 00000004 00000400 00000000 00000000 00000061
42002010 00000001 00020100 100d0610 00008000 00100018
00212000 00000000 00404000 90000000 00000000 00040611
040a0000 00000200

>D01051
00024006 01000c00 00100122 02010e00 20000000 00000010
00000004 20002001 00300800 00080be0 08300004 5003b000
04009002 00203000 00220040 00000000 00040000 02402080
08000000 00100a01 60100100 38030600 20020000 00500016
00110000 80000080 00000000 10000081 08008000 00068013
80185000 00806600

>D06396
00008002 89001c08 20100108 80010600 00001000 00020100
06000000 22001400 00000000 001009e8 08200004 50018800
02008806 08000000 81000441 00002008 00000000 02000400
00002000 00020601 40000000 180e8730 20000000 0050001c
00002000 c0000400 00100800 90000000 00000300 00060811
a4060000 00022200

>D06056
0000000a 01000808 20100108 04010e10 00043008 00000000

060c0000 81086000 00000300 000008d3 00201044 40218000
80001806 00400000 01000041 001c000c 00008000 02000000
00200400 00020001 40080000 1c018008 60840002 80500010
02042000 80000800 48020100 10000004 00000238 008e0000
c01c0a00 00000320

>D05938
00014002 01005400 00101100 00010700 20100080 00000000
00000080 20000000 00000080 004809e0 002c0204 d0019080
0000a012 00100000 020a3040 00084008 10040000 02000008
08404000 00000205 60100800 58280680 33002000 81500018
02002000 04000000 08800200 10000085 04008200 00060013
10000000 00c12e00

>D05792
00000040 01000008 20100102 00010700 00081081 00004000
02000000 20000800 00000980 000808c0 a8300000 40018000
00000002 00a00000 00080000 00083008 10800000 02000000
00402000 40020201 00000120 18200030 03000000 81500010
00000400 80000000 00520000 10000000 04000200 00060002
40000000 00002600

>D05740
00000006 00808808 40100100 41410610 00041040 00008004
0000a000 00001800 00400100 00090840 0820200c 40008800
90000800 00400002 00040040 000c000c 08008000 02000004
00600400 00200000 c0000000 18208048 21800040 91400010
00042000 80000000 08008040 00000000 04400000 00060002
80040200 00010720

>D05341
00010000 01000000 00000000 00000000 00080000 00000000
00000000 00000000 00000000 00000000 00000000 40001000
04008008 00800000 00000000 00080008 00000000 00000000
00402000 00000001 00000000 00000a00 01000000 00000010
00000000 00000000 00000000 00000000 00000000 00000000
00020000 00000000

>D05246
0000200a 01801219 40101500 10010618 04080040 08028408
00002004 00081004 80400300 500c18d0 20088028 42008800
800098a0 00300000 20030080 080c200c 00028000 87000800
04480008 50010001 84060100 18008b40 3100c044 8140441c
00040000 c1200a00 10000010 10001040 04084005 00c60809

c00c0284 28000408

>D05113
04051000 63000208 2010050a 00210700 001810e1 c2000002
07001000 20008241 00050880 010818c0 08340008 5081d140
0480c01a 23a10080 110c0021 120ca018 18800200 a6008000
0f482008 00020201 00000100 18210210 03004040 83500410
00000c00 c0000001 00120208 10000000 04000302 0006c80a
40020000 00002e40

>D04716
00000000 01000200 00100102 00010700 00180040 00000000
00000000 01000040 00000800 000808c0 08300000 40018000
00008000 00200000 00040000 000c1008 08820000 02000000
04402000 00000001 00010120 18000a10 01000000 00500010
00000400 a0000000 00000200 50000000 00000280 0006000a
00020000 00000e00

>D04625
00000000 4b000008 20100102 00010600 00000080 c0004000
02000000 00000000 00000980 00080840 08300000 40008000
00000002 00200000 00000000 00088008 10000000 02000000
00400000 00020201 00000100 18200020 03000000 81400010
00000000 80000000 00520000 00000000 04000000 00060002
40000000 00000600

>D04375
00008008 00000000 00000100 00012a00 0000a000 00000020
00000000 81084000 00000100 00000843 00000000 40008800
82000004 00000400 00000000 00080000 00000000 02800000
00000400 00000000 00080000 08000100 40040000 00400000
00002000 80000a00 40000100 00000000 00000028 000e0000
40100800 00020000

>D04040
0001014a 01800a00 08101500 52050610 00080045 04904000
810002a3 08001500 81800904 18f838d0 00308208 c0009880
8c009890 00100000 00033082 000c004c 10008002 0e300008
00490018 40010807 80000900 580a8aa0 21208040 81410414
03441800 c6000000 80400081 00009684 84010004 80c6080a
cc0c0084 20c10412

>D04034
0020c002 00820a08 20120100 01110600 20040000 0800e000

0a010450 05000000 00022100 00480d60 08244004 61008800
02008006 00200001 20020040 0208000a 04841000 22000000
08200002 02024000 e2100000 18020b60 20804000 0058001d
00006401 b0000300 12324010 40000080 02008280 01060012
c000a000 00030620

>D04006
0000002a 00801800 00080000 02000600 00080040 0000c040
00008000 00000000 06400100 800000a0 0c00800c 40010000
00009000 00c00000 00280080 000c000c 00000000 00008000
00400000 00000000 80000100 10000050 21800041 00300014
00040000 c0200000 60000000 00040000 00004000 00460000
c0080000 00000280

>D03881
00040000 43000208 20100102 00010600 00200000 80000000
02000000 20000000 00000880 000808c0 08300000 4000d000
14008002 00200000 00000000 000c8008 00000000 02000000
01400000 00020201 00000100 18200a00 05000000 81400010
00000000 80000000 00120000 10000000 04000002 0006000a
00000000 00002600

>D03880
00050040 43000208 20100102 00010700 80280040 80000000
02002800 28000000 00200880 010808e0 08300000 4000d000
1400802a 02a00000 00000000 002e9008 00800000 02000100
1140a000 00020201 00100120 18200a00 05000000 89400010
00000400 80000000 00120000 34000020 04000200 0006000a
00a00000 00002600

>D03879
00000000 43000208 20100102 00010600 00000000 80000000
02000000 20000000 00000880 000808c0 08300000 40008000
00000002 00200000 00000000 00048008 00000000 02000000
00000000 00020201 00000100 18200000 00000000 81400010
00000000 80000000 00120000 10000000 04000000 0006000a
00000000 00002600

>D03858
00000000 01000000 00100102 00010600 00100000 00004000
00000000 21000000 00100900 000808c0 08300000 50018000
00000002 00200000 00000000 00080008 00000000 02000000
00400000 00000201 00000100 18000020 01000000 00500010
00000000 a0000000 00000000 50000000 00000080 00060002

40000000 00002e00

>D03654
04031000 41000208 2010010a 00010600 00001041 02000010
02040000 21000240 00300880 000808c0 08300008 40039000
0580c012 00210000 410c0001 100c2018 08000000 06000000
0c400000 00020201 00000100 18210620 01000000 81500010
00000400 a0000000 00120000 50800004 04000380 0006000a
40002000 00802600

>D03642
00008050 20800200 01000100 01b10600 00080040 10000000
00010000 00000000 00000100 00480840 08002000 60009800
03008005 00000001 c0240000 00080008 08002000 02000000
00400000 00006400 80000000 18000b00 01000000 99400018
02002000 80000004 10000000 0000000c 04000400 20060002
00002000 00030460

>D03621
00000000 00000000 00000100 04010600 00000000 00000000
00000000 03001000 00000000 00090840 08200000 40008000
00000802 00002000 00000400 00000000 00000000 02000000
00000000 00000000 00000000 28008000 00000020 02400010
00000000 e0000000 00000000 c0000000 08000180 00060002
00040000 00000600

>D03503
00000042 40008000 21004000 00300c10 00090040 00000002
00000004 00002000 00000100 02014002 10208000 42000800
00004005 00340008 00020010 001c8004 24800800 02200300
00000000 40001080 00000102 00010000 21200600 08000000
00440000 01000000 42004000 00010220 00002014 00460000
40000001 02032200

>D03490
04000000 00000008 20100108 00010700 00001080 02000000
02000000 00000040 00002000 00000840 00200000 40008000
00800002 01000000 01200001 0008b008 10200000 00000000
00c00002 00020201 00000020 18000000 07000080 08000010
00000401 00000000 00080000 00800020 00000200 00060000
00000000 02020240

>D03415
04002002 00000008 20100108 00210602 00041080 02000000

06080000 00000008 00000000 00000840 00200004 40008000
00804806 00420000 01281041 020ca00c 10200020 00000004
00a00000 00060200 40000000 1c048008 37800080 88000010
00002001 00000000 00000000 00810001 00000200 00060008
00040200 002082c0

>D03274
0020c002 00820a08 20120101 01110600 20040000 0800e000
0a010450 05000000 00022100 00480d60 08244004 61008800
02008006 00200001 20020040 0208000a 04841000 22000000
08300002 02024000 e2100000 18020b60 20804000 0058001d
00006401 b0000300 12324010 40000080 02008280 01060012
c000a000 00030620

>D03210
10004002 01809c00 01184102 14050e10 00041020 10008040
10004040 22005002 00108948 000809e1 09310004 50018000
81018812 00208000 00820400 020d001e 00020400 02002404
00204400 00800601 c0020100 180c8e00 20800000 0050001c
00040000 c0000004 48126040 92000002 0040010c 00060013
e0080808 a8002620

>D03187
00050000 01000000 00000800 00010100 00080000 00000000
00000000 00000402 00000000 00108000 00000200 40009000
14028008 00800010 00000404 00080008 00820000 00000000
00402000 02000001 00000000 00000a00 01008000 00000010
00200000 00000000 00404000 80000000 00000200 81000000
24020000 00000000

>D03165
02014052 29d0fc00 01900302 c5910710 000c1180 00038010
c08a38c0 a420040b 00c10bc0 001909ea 08302004 5101f600
be02893e 01612b03 c05c124a 583e124c 30800640 82090544
00662d16 00004201 40800530 3a3486f8 33808063 9350003c
31243400 80081803 18da284c f0200021 0c404200 00260c13
a4044208 1a012620

>D02910
04403000 41200808 a038010b 04010602 00001281 0000810a
02050400 41200209 00024900 00080840 0c300020 40008c00
40888443 02202080 41083021 10080018 10004020 02010000
00100100 00021001 00410540 0806024a 52004000 00480014
08000012 a0004201 04520014 4a840015 02002280 01068002

80200000 40000600

>D02884
0000002a 00801800 00080000 02010600 00080040 0000c000
00008000 00000000 06400100 800000a0 0c00800c 40000000
00009000 00400000 00280080 000c000c 00800000 00008000
00400000 00000000 80000100 10000040 23800041 00300014
00040000 c0200000 60000000 00040000 00004000 00460000
c0000000 00000280

>D02826
04006048 03000418 2010310a 00210f02 80081080 82000000
02010004 20002242 00100a00 000809e2 18300010 40018040
01801402 00200008 11081001 00182008 18000800 02028000
00580040 00020201 80002124 38050000 33000040 00500010
00010000 c0000102 40030000 10000081 2a000350 10064013
00100000 00022600

>D02725
04050000 01000008 20000808 01310600 00081000 02000000
02000000 28001402 00000000 005088c8 00002300 40019000
1482800a 00800001 81600405 00082008 02000000 00000000
00412010 0202c001 00000000 18000600 0500a000 18000010
02201000 00000400 00404200 8080000c 00000200 81060000
24020000 00010270

>D02721
00010200 01000000 00000800 00000000 00482000 00000000
00200000 28000402 00000000 00108008 00000280 40001008
04028000 00800000 00000404 00080008 00000000 00000000
00402010 00000001 00000008 00000a00 01008800 00000010
00201000 00000408 00604200 80000000 00000000 01004000
24020800 00000000

>D02671
00205002 01c20600 00120000 00900600 20040200 00022000
00011010 20000000 00022000 00400de0 00644004 71018020
00000002 00000100 a0220140 02000002 04841000 20000000
08202002 02022201 e2100002 18000010 20800000 08180019
00004401 10000100 02204810 10000080 00008200 01060010
0102a000 00012220

>D02614
00000000 42000008 20100108 00010600 00001080 80004000

02040000 00000000 00000180 00080840 08200000 40008000
00004002 20000000 01080001 00088008 10000000 02000000
00080000 00020200 00000000 18200020 02000000 81400010
00000000 80000000 00520000 00000000 04000200 0006000a
40000000 00002600

>D02588
04010002 43003000 20120000 0d900450 04010240 00000010
80002020 2000000c 00400980 020f42c0 20208000 50053800
06018016 00142901 81020000 000c0004 04000000 0e000000
00000018 40400301 60000c00 30240622 21000243 16100018
18041200 00000200 00004000 10000140 0c000004 00460029
60004000 23012200

>D02578
00000000 00001008 20100108 00010600 00001000 00000000
02040000 00000004 00000080 00080840 08200000 40008000
00008002 00000000 01000001 00000008 00000000 02000000
00000000 00020200 00000000 18200608 00000000 81400018
00000000 80000000 00120000 00000000 04000200 00060003
00000000 00000600

>D02566
00000000 21000000 00100102 00010600 00000000 00000002
10000400 01000001 00100800 000808c0 08300000 40018000
00000000 00200000 00000020 00080000 00000200 02001000
02102000 00000001 00000100 18000010 00084000 00500010
00800000 a0000001 00000000 50000000 02000082 00060002
00020000 00000600

>D02374
04000000 40000008 20100108 00010600 00001081 02000000
02000000 00000000 00000110 00000840 00200000 40008000
00808202 00200080 01210001 00082008 10220000 22008000
00880000 00020200 00000000 18000a00 17000080 08400010
00000001 80000000 00080000 00800001 00000300 00060000
00000000 12000240

>D02361
00204002 00821a00 40120120 00110600 20040000 00002000
00010010 00000000 00422800 00480e50 08344004 61009000
00008000 00012800 20020040 12000002 04841000 22000001
08200002 02020001 c7100100 38060200 20800000 0058001f
00044401 90000300 02204010 00000080 28408200 01060002

8000a000 00010620

>D02359
00000000 00000008 20100100 00010600 00000080 00004000
02000000 00000000 00000980 00080840 08300000 40008000
00000002 00000000 00080000 00080008 10000000 02000000
00000000 00020201 00000100 18200020 03000000 81400010
00000000 80000000 00520000 00000000 04000000 00060002
40000000 00002600

>D02358
04000000 00000008 20100108 00010600 000010c0 02000000
02040000 00000040 00000000 00080840 08200000 40008000
00800002 00000000 01240001 00082008 18200000 02000000
04800000 00020200 00000000 18000000 07000080 08400010
00000001 80000000 00120000 00800000 00000200 00060002
00000000 00000640

>D02357
00000012 2910dc00 01900302 41910610 00045180 00000000
800822c0 04000403 004109c0 001909ea 08300004 51019000
98028902 00610800 000c0042 480e024c 30000600 82800144
00622412 00000201 40800500 1a308618 23808041 9140001c
01243000 80000801 0848284c d0000000 04404000 00260c53
24040200 02012620
