# Supplementary material for: iGPCR-Drug: A Web Server for Predicting Interaction between GPCRs and Drugs in Cellular Networking
Source: PLoS One. 2013 Aug 27;8(8):e72234. doi: 10.1371/journal.pone.0072234 (PMC3754978; doi:10.1371/journal.pone.0072234)
Supplement: Supporting Information S3 — The protein sequences for the GPCRs listed in Supporting Information S1. (PDF) [file pone.0072234.s003.pdf]

Online Supporting Information S3. The protein sequences for the GPCRs listed in Online Supporting Information S1.

>hsa:10161
MVSVNSSHCFYNDSFKYTLYGCMFSMVFVLGLISNCVAIYIFICVLKVRNETTTYMINLA
MSDLLFVFTLPFRIFYFTTRNWPFGDLLCKISVMLFYTNMYGSILFLTCISVDRFLAIVY
PFKSKTLRTKRNAKIVCTGVWLTVIGGSAPAVFVQSTHSQGNNASEACFENFPEATWKTY
LSRIVIFIEIVGFFIPLILNVTCSSMVLKTLTKPVTLSRSKINKTKVLKMIFVHLIIFCF
CFVPYNINLILYSLVRTQTFVNCSVVAAVRTMYPITLCIAVSNCCFDPIVYYFTSDTIQN
SIKMKNWSVRRSDFRFSEVHGAENFIQHNLQTLKSKIFDNESAA

>hsa:10800
MDETGNLTVSSATCHDTIDDFRNQVYSTLYSMISVVGFFGNGFVLYVLIKTYHKKSAFQV
YMINLAVADLLCVCTLPLRVVYYVHKGIWLFGDFLCRLSTYALYVNLYCSIFFMTAMSFF
RCIAIVFPVQNINLVTQKKARFVCVGIWIFVILTSSPFLMAKPQKDEKNNTKCFEPPQDN
QTKNHVLVLHYVSLFVGFIIPFVIIIVCYTMIILTLLKKSMKKNLSSHKKAIGMIMVVTA
AFLVSFMPYHIQRTIHLHFLHNETKPCDSVLRMQKSVVITLSLAASNCCFDPLLYFFSGG
NFRKRLSTFRKHSLSSVTYVPRKKASLPEKGEEICKV

>hsa:11255
MERAPPDGPLNASGALAGEAAAAGGARGFSAAWTAVLAALMALLIVATVLGNALVMLAFV
ADSSLRTQNNFFLLNLAISDFLVGAFCIPLYVPYVLTGRWTFGRGLCKLWLVVDYLLCTS
SAFNIVLISYDRFLSVTRAVSYRAQQGDTRRAVRKMLLVWVLAFLLYGPAILSWEYLSGG
SSIPEGHCYAEFFYNWYFLITASTLEFFTPFLSVTFFNLSIYLNIQRRTRLRLDGAREAA
GPEPPPEAQPSPPPPPGCWGCWQKGHGEAMPLHRYGVGEAAVGAEAGEATLGGGGGGGSV
ASPTSSSGSSSRGTERPRSLKRGSKPSASSASLEKRMKMVSQSFTQRFRLSRDRKVAKSL
AVIVSIFGLCWAPYTLLMIIRAACHGHCVPDYWYETSFWLLWANSAVNPVLYPLCHHSFR
RAFTKLLCPQKLKIQPHSSLEHCWK

>hsa:1128
MNTSAPPAVSPNITVLAPGKGPWQVAFIGITTGLLSLATVTGNLLVLISFKVNTELKTVN
NYFLLSLACADLIIGTFSMNLYTTYLLMGHWALGTLACDLWLALDYVASNASVMNLLLIS
FDRYFSVTRPLSYRAKRTPRRAALMIGLAWLVSFVLWAPAILFWQYLVGERTVLAGQCYI
QFLSQPIITFGTAMAAFYLPVTVMCTLYWRIYRETENRARELAALQGSETPGKGGGSSSS
SERSQPGAEGSPETPPGRCCRCCRAPRLLQAYSWKEEEEEDEGSMESLTSSEGEEPGSEV
VIKMPMVDPEAQAPTKQPPRSSPNTVKRPTKKGRDRAGKGQKPRGKEQLAKRKTFSLVKE
KKAARTLSAILLAFILTWTPYNIMVLVSTFCKDCVPETLWELGYWLCYVNSTINPMCYAL
CNKAFRDTFRLLLLCRWDKRRWRKIPKRPGSVHRTPSRQC

>hsa:1129
MNNSTNSSNNSLALTSPYKTFEVVFIVLVAGSLSLVTIIGNILVMVSIKVNRHLQTVNNY
FLFSLACADLIIGVFSMNLYTLYTVIGYWPLGPVVCDLWLALDYVVSNASVMNLLIISFD
RYFCVTKPLTYPVKRTTKMAGMMIAAAWVLSFILWAPAILFWQFIVGVRTVEDGECYIQF
FSNAAVTFGTAIAAFYLPVIIMTVLYWHISRASKSRIKKDKKEPVANQDPVSPSLVQGRI

VKPNNNNMPSSDDGLEHNKIQNGKAPRDPVTENCVQGEEKESSNDSTSVSAVASNMRDDE
ITQDENTVSTSLGHSKDENSKQTCIRIGTKTPKSDSCTPTNTTVEVVGSSGQNGDEKQNI
VARKIVKMTKQPAKKKPPPSREKKVTRTILAILLAFIITWAPYNVMVLINTFCAPCIPNT
VWTIGYWLCYINSTINPACYALCNATFKKTFKHLLMCHYKNIGATR

>hsa:1131
MTLHNNSTTSPLFPNISSSWIHSPSDAGLPPGTVTHFGSYNVSRAAGNFSSPDGTTDDPL
GGHTVWQVVFIAFLTGILALVTIIGNILVIVSFKVNKQLKTVNNYFLLSLACADLIIGVI
SMNLFTTYIIMNRWALGNLACDLWLAIDYVASNASVMNLLVISFDRYFSITRPLTYRAKR
TTKRAGVMIGLAWVISFVLWAPAILFWQYFVGKRTVPPGECFIQFLSEPTITFGTAIAAF
YMPVTIMTILYWRIYKETEKRTKELAGLQASGTEAETENFVHPTGSSRSCSSYELQQQSM
KRSNRRKYGRCHFWFTTKSWKPSSEQMDQDHSSSDSWNNNDAASLENSASSDEEDIGSE
TRAIYSIVLKLPGHSTILNSTKLPSSDNLQVPEEELGMVDLERKADKLQAQKSVDDGGSF
PKSFSKLPIQLESAVDTAKTSDVNSSVGKSTATLPLSFKEATLAKRFALKTRSQITKRKR
MSLVKEKKAAQTLSAILLAFIITWTPYNIMVLVNTFCDSCIPKTFWNLGYWLCYINSTVN
PVCYALCNKTFRTTFKMLLLCQCDKKKRRKQQYQQRQSVIFHKRAPEQAL

>hsa:1132
MANFTPVNGSSGNQSVRLVTSSSHNRYETVEMVFIATVTGSLSLVTVVGNILVMLSIKVN
RQLQTVNNYFLFSLACADLIIGAFSMNLYTVYIIKGYWPLGAVVCDLWLALDYVVSNASV
MNLLIISFDRYFCVTKPLTYPARRTTKMAGLMIAAAWVLSFVLWAPAILFWQFVVGKRTV
PDNQCFIQFLSNPAVTFGTAIAAFYLPVVIMTVLYIHISLASRSRVHKHRPEGPKEKKAK
TLAFLKSPLMKQSVKKPPPGEAAREELRNGKLEEAPPPALPPPPRPVADKDTSNESSSGS
ATQNTKERPATELSTTEATTPAMPAPPLQPRALNPASRWSKIQIVTKQTGNECVTAIEIV
PATPAGMRPAANVARKFASIARNQVRKKRQMAARERKVTRTIFAILLAFILTWTPYNVMV
LVNTFCQSCIPDTVWSIGYWLCYVNSTINPACYALCNATFKKTFRHLLLCQYRNIGTAR

>hsa:1133
MEGDSYHNATTVNGTPVNHQPLERHRLWEVITIAAVTAVVSLITIVGNVLVMISFKVNSQ
LKTVNNYYLLSLACADLIIGIFSMNLYTTYILMGRWALGSLACDLWLALDYVASNASVMN
LLVISFDRYFSITRPLTYRAKRTPKRAGIMIGLAWLISFILWAPAILCWQYLVGKRTVPL
DECQIQFLSEPTITFGTAIAAFYIPVSVMTILYCRIYRETEKRTKDLADLQGSDSVTKAE
KRKPAHRALFRSCLRCPRPTLAQRERNQASWSSSRRSTSTTGKPSQATGPSANWAKAEQL
TTCSSYPSSEDEDKPATDPVLQVVYKSQGKESPGEEFSAEETEETFVKAETEKSDYDTPN
YLLSPAAAHRPKSQKCVAYKFRLVVKADGNQETNNGCHKVKIMPCPFPVAKEPSTKGLNP
NPSHQMTKRKRVVLVKERKAAQTLSAILLAFIITWTPYNIMVLVSTFCDKCVPVTLWHLG
YWLCYVNSTVNPICYALCNRTFRKTFKMLLLCRWKKKKVEEKLYWQGNSKLP

>hsa:1234
MDYQVSSPIYDINYYTSEPCQKINVKQIAARLLPPLYSLVFIFGFVGNMLVILILINCKR
LKSMTDIYLLNLAISDLFFLLTVPFWAHYAAAQWDFGNTMCQLLTGLYFIGFFSGIFFII
LLTIDRYLAVVHAVFALKARTVTFGVVTSVITWVVAVFASLPGIIFTRSQKEGLHYTCSS
HFPYSQYQFWKNFQTLKIVILGLVLPLLVMVICYSGILKTLLRCRNEKKRHRAVRLIFTI
MIVYFLFWAPYNIVLLLNTFQEFFGLNNCSSSNRLDQAMQVTETLGMTHCCINPIIYAFV

GEKFRNYLLVFFQKHIAKRFCKCCSIFQQEAPERASSVYTRSTGEQEISVGL

>hsa:1241
MNTTSSAAPPSLGVEFISLLAIILLSVALAVGLPGNSFVVWSILKRMQKRSVTALMVLNL
ALADLAVLLTAPFFLHFLAQGTWSFGLAGCRLCHYVCGVSMYASVLLITAMSLDRSLAVA
RPFVSQKLRTKAMARRVLAGIWVLSFLLATPVLAYRTVVPWKTNMSLCFPRYPSEGHRAF
HLIFEAVTGFLLPFLAVVASYSDIGRRLQARRFRRSRRTGRLVVLIILTFAAFWLPYHVV
NLAEAGRALAGQAAGLGLVGKRLSLARNVLIALAFLSSSVNPVLYACAGGGLLRSAGVGF
VAKLLEGTGSEASSTRRGGSLGQTARSGPAALEPGPSESLTASSPLKLNELN

>hsa:1268
MKSILDGLADTTFRTITTDLLYVGSNDIQYEDIKGDMASKLGYFPQKFPLTSFRGSPFQE
KMTAGDNPQLVPADQVNITEFYNKSLSSFKENEENIQCGENFMDIECFMVLNPSQQLAIA
VLSLTLGTFTVLENLLVLCVILHSRSLRCRPSYHFIGSLAVADLLGSVIFVYSFIDFHVF
HRKDSRNVFLFKLGGVTASFTASVGSLFLTAIDRYISIHRPLAYKRIVTRPKAVVAFCLM
WTIAIVIAVLPLLGWNCEKLQSVCSDIFPHIDETYLMFWIGVTSVLLLFIVYAYMYILWK
AHSHAVRMIQRGTQKSIIIHTSEDGKVQVTRPDQARMDIRLAKTLVLILVVLIICWGPLL
AIMVYDVFGKMNKLIKTVFAFCSMLCLLNSTVNPIIYALRSKDLRHAFRSMFPSCEGTAQ
PLDNSMGDSDCLHKHANNAASVHRAAESCIKSTVKIAKVTMSVSTDTSAEAL

>hsa:134
MPPSISAFQAAYIGIEVLIALVSVPGNVLVIWAVKVNQALRDATFCFIVSLAVADVAVGA
LVIPLAILINIGPQTYFHTCLMVACPVLILTQSSILALLAIAVDRYLRVKIPLRYKMVVT
PRRAAVAIAGCWILSFVVGLTPMFGWNNLSAVERAWAANGSMGEPVIKCEFEKVISMEYM
VYFNFFVWVLPPLLLMVLIYLEVFYLIRKQLNKKVSASSGDPQKYYGKELKIAKSLALIL
FLFALSWLPLHILNCITLFCPSCHKPSILTYIAIFLTHGNSAMNPIVYAFRIQKFRVTFL
KIWNDHFRCQPAPPIDEDLPEERPDD

>hsa:135
MPIMGSSVYITVELAIAVLAILGNVLVCWAVWLNSNLQNVTNYFVVSLAAADIAVGVLAI
PFAITISTGFCAACHGCLFIACFVLVLTQSSIFSLLAIAIDRYIAIRIPLRYNGLVTGTR
AKGIIAICWVLSFAIGLTPMLGWNNCGQPKEGKNHSQGCGEGQVACLFEDVVPMNYMVYF
NFFACVLVPLLLMLGVYLRIFLAARRQLKQMESQPLPGERARSTLQKEVHAAKSLAIIVG
LFALCWLPLHIINCFTFFCPDCSHAPLWLMYLAIVLSHTNSVVNPFIYAYRIREFRQTFR
KIIRSHVLRQQEPFKAAGTSARVLAAHGSDGEQVSLRLNGHPPGVWANGSAPHPERRPNG
YALGLVSGGSAQESQGNTGLPDVELLSHELKGVCPEPPGLDDPLAQDGAGVS

>hsa:136
MLLETQDALYVALELVIAALSVAGNVLVCAAVGTANTLQTPTNYFLVSLAAADVAVGLFA
IPFAITISLGFCTDFYGCLFLACFVLVLTQSSIFSLLAVAVDRYLAICVPLRYKSLVTGT
RARGVIAVLWVLAFGIGLTPFLGWNSKDSATNNCTEPWDGTTNESCCLVKCLFENVVPMS
YMVYFNFFGCVLPPLLIMLVIYIKIFLVACRQLQRTELMDHSRTTLQREIHAAKSLAMIV
GIFALCWLPVHAVNCVTLFQPAQGKNKPKWAMNMAILLSHANSVVNPIVYAYRNRDFRYT

FHKIISRYLLCQADVKSGNGQAGVQPALGVGL

>hsa:140
MPNNSTALSLANVTYITMEIFIGLCAIVGNVLVICVVKLNPSLQTTTFYFIVSLALADIA
VGVLVMPLAIVVSLGITIHFYSCLFMTCLLLIFTHASIMSLLAIAVDRYLRVKLTVRYKR
VTTHRRIWLALGLCWLVSFLVGLTPMFGWNMKLTSEYHRNVTFLSCQFVSVMRMDYMVYF
SFLTWIFIPLVVMCAIYLDIFYIIRNKLSLNLSNSKETGAFYGREFKTAKSLFLVLFLFA
LSWLPLSIINCIIYFNGEVPQLVLYMGILLSHANSMMNPIVYAYKIKKFKETYLLILKAC
VVCHPSDSLDTSIEKNSE

>hsa:146
MTFRDLLSVSFEGPRPDSSAGGSSAGGGGGSAGGAAPSEGPAVGGVPGGAGGGGGVVGAG
SGEDNRSSAGEPGSAGAGGDVNGTAAVGGLVVSAQGVGVGVFLAAFILMAVAGNLLVILS
VACNRHLQTVTNYFIVNLAVADLLLSATVLPFSATMEVLGFWAFGRAFCDVWAAVDVLCC
TASILSLCTISVDRYVGVRHSLKYPAIMTERKAAAILALLWVVALVVSVGPLLGWKEPVP
PDERFCGITEEAGYAVFSSVCSFYLPMAVIVVMYCRVYVVARSTTRSLEAGVKRERGKAS
EVVLRIHCRGAATGADGAHGMRSAKGHTFRSSLSVRLLKFSREKKAAKTLAIVVGVFVLC
WFPFFFVLPLGSLFPQLKPSEGVFKVIFWLGYFNSCVNPLIYPCSSREFKRAFLRLLRCQ
CRRRRRRRPLWRVYGHHWRASTSGLRQDCAPSSGDAPPGAPLALTALPDPDPEPPGTPEM
QAPVASRRKPPSAFREWRLLGPFRRPTTQLRAKVSSLSHKIRAGGAQRAEAACAQRSEVE
AVSLGVPHEVAEGATCQAYELADYSNLRETDI

>hsa:147
MNPDLDTGHNTSAPAHWGELKNANFTGPNQTSSNSTLPQLDITRAISVGLVLGAFILFAI
VGNILVILSVACNRHLRTPTNYFIVNLAMADLLLSFTVLPFSAALEVLGYWVLGRIFCDI
WAAVDVLCCTASILSLCAISIDRYIGVRYSLQYPTLVTRRKAILALLSVWVLSTVISIGP
LLGWKEPAPNDDKECGVTEEPFYALFSSLGSFYIPLAVILVMYCRVYIVAKRTTKNLEAG
VMKEMSNSKELTLRIHSKNFHEDTLSSTKAKGHNPRSSIAVKLFKFSREKKAAKTLGIVV
GMFILCWLPFFIALPLGSLFSTLKPPDAVFKVVFWLGYFNSCLNPIIYPCSSKEFKRAFV
RILGCQCRGRGRRRRRRRRRLGGCAYTYRPWTRGGSLERSQSRKDSLDDSGSCLSGSQRT
LPSASPSPGYLGRGAPPPVELCAFPEWKAPGALLSLPAPEPPGRRGRHDSGPLFTFKLLT
EPESPGTDGGASNGGCEAAADVANGQPGFKSNMPLAPGQF

>hsa:148
MVFLSGNASDSSNCTQPPAPVNISKAILLGVILGGLILFGVLGNILVILSVACHRHLHSV
THYYIVNLAVADLLLTSTVLPFSAIFEVLGYWAFGRVFCNIWAAVDVLCCTASIMGLCII
SIDRYIGVSYPLRYPTIVTQRRGLMALLCVWALSLVISIGPLFGWRQPAPEDETICQINE
EPGYVLFSALGSFYLPLAIILVMYCRVYVVAKRESRGLKSGLKTDKSDSEQVTLRIHRKN
APAGGSGMASAKTKTHFSVRLLKFSREKKAAKTLGIVVGCFVLCWLPFFLVMPIGSFFPD
FKPSETVFKIVFWLGYLNSCINPIIYPCSSQEFKKAFQNVLRIQCLCRKQSSKHALGYTL
HPPSQAVEGQHKDMVRIPVGSRETFYRISKTDGVCEWKFFSSMPRGSARITVSKDQSSCT
TARVRSKSFLQVCCCVGPSTPSLDKNHQVPTIKVHTISLSENGEEV

>hsa:150
MFRQEQPLAEGSFAPMGSLQPDAGNASWNGTEAPGGGARATPYSLQVTLTLVCLAGLLML
LTVFGNVLVIIAVFTSRALKAPQNLFLVSLASADILVATLVIPFSLANEVMGYWYFGKAW
CEIYLALDVLFCTSSIVHLCAISLDRYWSITQAIEYNLKRTPRRIKAIIITVWVISAVIS
FPPLISIEKKGGGGGPQPAEPRCEINDQKWYVISSCIGSFFAPCLIMILVYVRIYQIAKR
RTRVPPSRRGPDAVAAPPGGTERRPNGLGPERSAGPGGAEAEPLPTQLNGAPGEPAPAGP
RDTDALDLEESSSSDHAERPPGPRRPERGPRGKGKARASQVKPGDSLPRRGPGATGIGTP
AAGPGEERVGAAKASRWRGRQNREKRFTFVLAVVIGVFVVCWFPFFFTYTLTAVGCSVPR
TLFKFFFWFGYCNSSLNPVIYTIFNHDFRRAFKKILCRGDRKRIV

>hsa:151
MDHQDPYSVQATAAIAAAITFLILFTIFGNALVILAVLTSRSLRAPQNLFLVSLAAADIL
VATLIIPFSLANELLGYWYFRRTWCEVYLALDVLFCTSSIVHLCAISLDRYWAVSRALEY
NSKRTPRRIKCIILTVWLIAAVISLPPLIYKGDQGPQPRGRPQCKLNQEAWYILASSIGS
FFAPCLIMILVYLRIYLIAKRSNRRGPRAKGGPGQGESKQPRPDHGGALASAKLPALASV
ASAREVNGHSKSTGEKEEGETPEDTGTRALPPSWAALPNSGQGQKEGVCGASPEDEAEEE
EEEEEEEEECEPQAVPVSPASACSPPLQQPQGSRVLATLRGQVLLGRGVGAIGGQWWRRR
AQLTREKRFTFVLAVVIGVFVLCWFPFFFSYSLGAICPKHCKVPHGLFQFFFWIGYCNSS
LNPVIYTIFNQDFRRAFRRILCRPWTQTAW

>hsa:152
MASPALAAALAVAAAAGPNASGAGERGSGGVANASGASWGPPRGQYSAGAVAGLAAVVGF
LIVFTVVGNVLVVIAVLTSRALRAPQNLFLVSLASADILVATLVMPFSLANELMAYWYFG
QVWCGVYLALDVLFCTSSIVHLCAISLDRYWSVTQAVEYNLKRTPRRVKATIVAVWLISA
VISFPPLVSLYRQPDGAAYPQCGLNDETWYILSSCIGSFFAPCLIMGLVYARIYRVAKLR
TRTLSEKRAPVGPDGASPTTENGLGAAAGAGENGHCAPPPADVEPDESSAAAERRRRRGA
LRRGGRRRAGAEGGAGGADGQGAGPGAAESGALTASRSPGPGGRLSRASSRSVEFFLSRR
RRARSSVCRRKVAQAREKRFTFVLAVVMGVFVLCWFPFFFSYSLYGICREACQVPGPLFK
FFFWIGYCNSSLNPVIYTVFNQDFRRSFKHILFRRRRRGFRQ

>hsa:153
MGAGVLVLGASEPGNLSSAAPLPDGAATAARLLVPASPPASLLPPASESPEPLSQQWTAG
MGLLMALIVLLIVAGNVLVIVAIAKTPRLQTLTNLFIMSLASADLVMGLLVVPFGATIVV
WGRWEYGSFFCELWTSVDVLCVTASIETLCVIALDRYLAITSPFRYQSLLTRARARGLVC
TVWAISALVSFLPILMHWWRAESDEARRCYNDPKCCDFVTNRAYAIASSVVSFYVPLCIM
AFVYLRVFREAQKQVKKIDSCERRFLGGPARPPSPSPSPVPAPAPPPGPPRPAAAAATAP
LANGRAGKRRPSRLVALREQKALKTLGIIMGVFTLCWLPFFLANVVKAFHRELVPDRLFV
FFNWLGYANSAFNPIIYCRSPDFRKAFQGLLCCARRAARRRHATHGDRPRASGCLARPGP
PPSPGAASDDDDDDVVGATPPARLLEPWAGCNGGAAADSDSSLDEPCRPGFASESKV

>hsa:154
MGQPGNGSAFLLAPNRSHAPDHDVTQQRDEVWVVGMGIVMSLIVLAIVFGNVLVITAIAK
FERLQTVTNYFITSLACADLVMGLAVVPFGAAHILMKMWTFGNFWCEFWTSIDVLCVTAS
IETLCVIAVDRYFAITSPFKYQSLLTKNKARVIILMVWIVSGLTSFLPIQMHWYRATHQE

AINCYANETCCDFFTNQAYAIASSIVSFYVPLVIMVFVYSRVFQEAKRQLQKIDKSEGRF
HVQNLSQVEQDGRTGHGLRRSSKFCLKEHKALKTLGIIMGTFTLCWLPFFIVNIVHVIQD
NLIRKEVYILLNWIGYVNSGFNPLIYCRSPDFRIAFQELLCLRRSSLKAYGNGYSSNGNT
GEQSGYHVEQEKENKLLCEDLPGTEDFVGHQGTVPSDNIDSQGRNCSTNDSLL

>hsa:155
MAPWPHENSSLAPWPDLPTLAPNTANTSGLPGVPWEAALAGALLALAVLATVGGNLLVIV
AIAWTPRLQTMTNVFVTSLAAADLVMGLLVVPPAATLALTGHWPLGATGCELWTSVDVLC
VTASIETLCALAVDRYLAVTNPLRYGALVTKRCARTAVVLVWVVSAAVSFAPIMSQWWRV
GADAEAQRCHSNPRCCAFASNMPYVLLSSSVSFYLPLLVMLFVYARVFVVATRQLRLLRG
ELGRFPPEESPPAPSRSLAPAPVGTCAPPEGVPACGRRPARLLPLREHRALCTLGLIMGT
FTLCWLPFFLANVLRALGGPSLVPGPAFLALNWLGYANSAFNPLIYCRSPDFRSAFRRLL
CRCGRRLPPEPCAAARPALFPSGVPAARSSPAQPRLCQRLDGASWGVS

>hsa:1812
MRTLNTSAMDGTGLVVERDFSVRILTACFLSLLILSTLLGNTLVCAAVIRFRHLRSKVTN
FFVISLAVSDLLVAVLVMPWKAVAEIAGFWPFGSFCNIWVAFDIMCSTASILNLCVISVD
RYWAISSPFRYERKMTPKAAFILISVAWTLSVLISFIPVQLSWHKAKPTSPSDGNATSLA
ETIDNCDSSLSRTYAISSSVISFYIPVAIMIVTYTRIYRIAQKQIRRIAALERAAVHAKN
CQTTTGNGKPVECSQPESSFKMSFKRETKVLKTLSVIMGVFVCCWLPFFILNCILPFCGS
GETQPFCIDSNTFDVFVWFGWANSSLNPIIYAFNADFRKAFSTLLGCYRLCPATNNAIET
VSINNNGAAMFSSHHEPRGSISKECNLVYLIPHAVGSSEDLKKEEAAGIARPLEKLSPAL
SVILDYDTDVSLEKIQPITQNGQHPT

>hsa:1813
MDPLNLSWYDDDLERQNWSRPFNGSDGKADRPHYNYYATLLTLLIAVIVFGNVLVCMAVS
REKALQTTTNYLIVSLAVADLLVATLVMPWVVYLEVVGEWKFSRIHCDIFVTLDVMMCTA
SILNLCAISIDRYTAVAMPMLYNTRYSSKRRVTVMISIVWVLSFTISCPLLFGLNNADQN
ECIIANPAFVVYSSIVSFYVPFIVTLLVYIKIYIVLRRRRKRVNTKRSSRAFRAHLRAPL
KGNCTHPEDMKLCTVIMKSNGSFPVNRRRVEAARRAQELEMEMLSSTSPPERTRYSPIPP
SHHQLTLPDPSHHGLHSTPDSPAKPEKNGHAKDHPKIAKIFEIQTMPNGKTRTSLKTMSR
RKLSQQKEKKATQMLAIVLGVFIICWLPFFITHILNIHCDCNIPPVLYSAFTWLGYVNSA
VNPIIYTTFNIEFRKAFLKILHC

>hsa:1814
MASLSQLSGHLNYTCGAENSTGASQARPHAYYALSYCALILAIVFGNGLVCMAVLKERAL
QTTTNYLVVSLAVADLLVATLVMPWVVYLEVTGGVWNFSRICCDVFVTLDVMMCTASILN
LCAISIDRYTAVVMPVHYQHGTGQSSCRRVALMITAVWVLAFAVSCPLLFGFNTTGDPTV
CSISNPDFVIYSSVVSFYLPFGVTVLVYARIYVVLKQRRRKRILTRQNSQCNSVRPGFPQ
QTLSPDPAHLELKRYYSICQDTALGGPGFQERGGELKREEKTRNSLSPTIAPKLSLEVRK
LSNGRLSTSLKLGPLQPRGVPLREKKATQMVAIVLGAFIVCWLPFFLTHVLNTHCQTCHV
SPELYSATTWLGYVNSALNPVIYTTFNIEFRKAFLKILSC

>hsa:1815

MGNRSTADADGLLAGRGPAAGASAGASAGLAGQGAAALVGGVLLIGAVLAGNSLVCVSVA
TERALQTPTNSFIVSLAAADLLLALLVLPLFVYSEVQGGAWLLSPRLCDALMAMDVMLCT
ASIFNLCAISVDRFVAVAVPLRYNRQGGSRRQLLLIGATWLLSAAVAAPVLCGLNDVRGR
DPAVCRLEDRDYVVYSSVCSFFLPCPLMLLLYWATFRGLQRWEVARRAKLHGRAPRRPSG
PGPPSPTPPAPRLPQDPCGPDCAPPAPGLPRGPCGPDCAPAAPSLPQDPCGPDCAPPAPG
LPPDPCGSNCAPPDAVRAAALPPQTPPQTRRRRRAKITGRERKAMRVLPVVVGAFLLCWT
PFFVVHITQALCPACSVPPRLVSAVTWLGYVNSALNPVIYTVFNAEFRNVFRKALRACC

>hsa:1816
MLPPGSNGTAYPGQFALYQQLAQGNAVGGSAGAPPLGPSQVVTACLLTLLIIWTLLGNVL
VCAAIVRSRHLRANMTNVFIVSLAVSDLFVALLVMPWKAVAEVAGYWPFGAFCDVWVAFD
IMCSTASILNLCVISVDRYWAISRPFRYKRKMTQRMALVMVGLAWTLSILISFIPVQLNW
HRDQAASWGGLDLPNNLANWTPWEEDFWEPDVNAENCDSSLNRTYAISSSLISFYIPVAI
MIVTYTRIYRIAQVQIRRISSLERAAEHAQSCRSSAACAPDTSLRASIKKETKVLKTLSV
IMGVFVCCWLPFFILNCMVPFCSGHPEGPPAGFPCVSETTFDVFVWFGWANSSLNPVIYA
FNADFQKVFAQLLGCSHFCSRTPVETVNISNELISYNQDIVFHKEIAAAYIHMMPNAVTP
GNREVDNDEEEGPFDRMFQIYQTSPDGDPVAESVWELDCEGEISLDKITPFTPNGFH

>hsa:185
MILNSSTEDGIKRIQDDCPKAGRHNYIFVMIPTLYSIIFVVGIFGNSLVVIVIYFYMKLK
TVASVFLLNLALADLCFLLTLPLWAVYTAMEYRWPFGNYLCKIASASVSFNLYASVFLLT
CLSIDRYLAIVHPMKSRLRRTMLVAKVTCIIIWLLAGLASLPAIIHRNVFFIENTNITVC
AFHYESQNSTLPIGLGLTKNILGFLFPFLIILTSYTLIWKALKKAYEIQKNKPRNDDIFK
IIMAIVLFFFFSWIPHQIFTFLDVLIQLGIIRDCRIADIVDTAMPITICIAYFNNCLNPL
FYGFLGKKFKRYFLQLLKYIPPKAKSHSNLSTKMSTLSYRPSDNVSSSTKKPAPCFEVE

>hsa:1909
METLCLRASFWLALVGCVISDNPERYSTNLSNHVDDFTTFRGTELSFLVTTHQPTNLVLP
SNGSMHNYCPQQTKITSAFKYINTVISCTIFIVGMVGNATLLRIIYQNKCMRNGPNALIA
SLALGDLIYVVIDLPINVFKFYQDVKDWWLFGFYFCMPLVCTAIFYTLMTCEMLNRRNGS
LRIALSEHLKQRREVAKTVFCLVVIFALCWFPLHLSRILKKTVYNEMDKNRCELLSFLLL
MDYIGINLATMNSCINPIALYFVSKKFKNCFQSCLCCCCYQSKSLMTSVPMNGTSIQWKN
HDQNNHNTDRSSHKDSMN

>hsa:1910
MQPPPSLCGRALVALVLACGLSRIWGEERGFPPDRATPLLQTAEIMTPPTKTLWPKGSNA
SLARSLAPAEVPKGDRTAGSPPRTISPPPCQGPIEIKETFKYINTVVSCLVFVLGIIGNS
TLLRIIYKNKCMRNGPNILIASLALGDLLHIVIDIPINVYKLLAEDWPFGAEMCKLVPFI
QKASVGITVLSLCALSIDRYRAVASWSRIKGIGVPKWTAVEIVLIWVVSVVLAVPEAIGF
DIITMDYKGSYLRICLLHPVQKTAFMQFYKTAKDWWLFSFYFCLPLAITAFFYTLMTCEM
LRKKSGMQIALNDHLKQRREVAKTVFCLVLVFALCWLPLHLSRILKLTLYNQNDPNRCEL
LSFLLVLDYIGINMASLNSCINPIALYLVSKRFKNCFKSCLCCWCQSFEEKQSLEEKQSC
LKFKANDHGYDNFRSSNKYSSS

>hsa:222545
MAFLIILITCFVIILATSQPCQTPDDFVAATSPGHIIIGGLFAIHEKMLSSEDSPRRPQI
QECVGFEISVFLQTLAMIHSIEMINNSTLLPGVKLGYEIYDTCTEVTVAMAATLRFLSKF
NCSRETVEFKCDYSSYMPRVKAVIGSGYSEITMAVSRMLNLQLMPQVGYESTAEILSDKI
RFPSFLRTVPSDFHQIKAMAHLIQKSGWNWIGIITTDDDYGRLALNTFIIQAEANNVCIA
FKEVLPAFLSDNTIEVRINRTLKKIILEAQVNVIVVFLRQFHVFDLFNKAIEMNINKMWI
ASDNWSTATKITTIPNVKKIGKVVGFAFRRGNISSFHSFLQNLHLLPSDSHKLLHEYAMH
LSACAYVKDTDLSQCIFNHSQRTLAYKANKAIERNFVMRNDFLWDYAEPGLIHSIQLAVF
ALGYAIRDLCQARDCQNPNAFQPWELLGVLKNVTFTDGWNSFHFDAHGDLNTGYDVVLWK
EINGHMTVTKMAEYDLQNDVFIIPDQETKNEFRNLKQIQSKCSKECSPGQMKKTTRSQHI
CCYECQNCPENHYTNQTDMPHCLLCNNKTHWAPVRSTMCFEKEVEYLNWNDSLAILLLIL
SLLGIIFVLVVGIIFTRNLNTPVVKSSGGLRVCYVILLCHFLNFASTSFFIGEPQDFTCK
TRQTMFGVSFTLCISCILTKSLKILLAFSFDPKLQKFLKCLYRPILIIFTCTGIQVVICT
LWLIFAAPTVEVNVSLPRVIILECEEGSILAFGTMLGYIAILAFICFIFAFKGKYENYNE
AKFITFGMLIYFIAWITFIPIYATTFGKYVPAVEIIVILISNYGILYCTFIPKCYVIICK
QEINTKSAFLKMIYSYSSHSVSSIALSPASLDSMSGNVTMTNPSSSGKSATWQKSKDLQA
QAFAHICRENATSVSKTLPRKRMSSI

>hsa:23620
METSSPRPPRPSSNPGLSLDARLGVDTRLWAKVLFTALYALIWALGAAGNALSVHVVLKA
RAGRAGRLRHHVLSLALAGLLLLLVGVPVELYSFVWFHYPWVFGDLGCRGYYFVHELCAY
ATVLSVAGLSAERCLAVCQPLRARSLLTPRRTRWLVALSWAASLGLALPMAVIMGQKHEL
ETADGEPEPASRVCTVLVSRTALQVFIQVNVLVSFVLPLALTAFLNGVTVSHLLALCSQV
PSTSTPGSSTPSRLELLSEEGLLSFIVWKKTFIQGGQVSLVRHKDVRRIRSLQRSVQVLR
AIVVMYVICWLPYHARRLMYCYVPDDAWTDPLYNFYHYFYMVTNTLFYVSSAVTPLLYNA
VSSSFRKLFLEAVSSLCGEHHPMKRLPPKPQSPTLMDTASGFGDPPETRT

>hsa:2550
MLLLLLLAPLFLRPPGAGGAQTPNATSEGCQIIHPPWEGGIRYRGLTRDQVKAINFLPVD
YEIEYVCRGEREVVGPKVRKCLANGSWTDMDTPSRCVRICSKSYLTLENGKVFLTGGDLP
ALDGARVDFRCDPDFHLVGSSRSICSQGQWSTPKPHCQVNRTPHSERRAVYIGALFPMSG
GWPGGQACQPAVEMALEDVNSRRDILPDYELKLIHHDSKCDPGQATKYLYELLYNDPIKI
ILMPGCSSVSTLVAEAARMWNLIVLSYGSSSPALSNRQRFPTFFRTHPSATLHNPTRVKL
FEKWGWKKIATIQQTTEVFTSTLDDLEERVKEAGIEITFRQSFFSDPAVPVKNLKRQDAR
IIVGLFYETEARKVFCEVYKERLFGKKYVWFLIGWYADNWFKIYDPSINCTVDEMTEAVE
GHITTEIVMLNPANTRSISNMTSQEFVEKLTKRLKRHPEETGGFQEAPLAYDAIWALALA
LNKTSGGGGRSGVRLEDFNYNNQTITDQIYRAMNSSSFEGVSGHVVFDASGSRMAWTLIE
QLQGGSYKKIGYYDSTKDDLSWSKTDKWIGGSPPADQTLVIKTFRFLSQKLFISVSVLSS
LGIVLAVVCLSFNIYNSHVRYIQNSQPNLNNLTAVGCSLALAAVFPLGLDGYHIGRNQFP
FVCQARLWLLGLGFSLGYGSMFTKIWWVHTVFTKKEEKKEWRKTLEPWKLYATVGLLVGM
DVLTLAIWQIVDPLHRTIETFAKEEPKEDIDVSILPQLEHCSSRKMNTWLGIFYGYKGLL
LLLGIFLAYETKSVSTEKINDHRAVGMAIYNVAVLCLITAPVTMILSSQQDAAFAFASLA
IVFSSYITLVVLFVPKMRRLITRGEWQSEAQDTMKTGSSTNNNEEEKSRLLEKENRELEK
IIAEKEERVSELRHQLQSRQQLRSRRHPPTPPEPSGGLPRGPPEPPDRLSCDGSRVHLLY

K

>hsa:2846
MGDRRFIDFQFQDSNSSLRPRLGNATANNTCIVDDSFKYNLNGAVYSVVFILGLITNSVS
LFVFCFRMKMRSETAIFITNLAVSDLLFVCTLPFKIFYNFNRHWPFGDTLCKISGTAFLT
NIYGSMLFLTCISVDRFLAIVYPFRSRTIRTRRNSAIVCAGVWILVLSGGISASLFSTTN
VNNATTTCFEGFSKRVWKTYLSKITIFIEVVGFIIPLILNVSCSSVVLRTLRKPATLSQI
GTNKKKVLKMITVHMAVFVVCFVPYNSVLFLYALVRSQAITNCFLERFAKIMYPITLCLA
TLNCCFDPFIYYFTLESFQKSFYINAHIRMESLFKTETPLTTKPSLPAIQEEVSDQTTNN
GGELMLESTF

>hsa:2911
MVGLLLFFFPAIFLEVSLLPRSPGRKVLLAGASSQRSVARMDGDVIIGALFSVHHQPPAE
KVPERKCGEIREQYGIQRVEAMFHTLDKINADPVLLPNITLGSEIRDSCWHSSVALEQSI
EFIRDSLISIRDEKDGINRCLPDGQSLPPGRTKKPIAGVIGPGSSSVAIQVQNLLQLFDI
PQIAYSATSIDLSDKTLYKYFLRVVPSDTLQARAMLDIVKRYNWTYVSAVHTEGNYGESG
MDAFKELAAQEGLCIAHSDKIYSNAGEKSFDRLLRKLRERLPKARVVVCFCEGMTVRGLL
SAMRRLGVVGEFSLIGSDGWADRDEVIEGYEVEANGGITIKLQSPEVRSFDDYFLKLRLD
TNTRNPWFPEFWQHRFQCRLPGHLLENPNFKRICTGNESLEENYVQDSKMGFVINAIYAM
AHGLQNMHHALCPGHVGLCDAMKPIDGSKLLDFLIKSSFIGVSGEEVWFDEKGDAPGRYD
IMNLQYTEANRYDYVHVGTWHEGVLNIDDYKIQMNKSGVVRSVCSEPCLKGQIKVIRKGE
VSCCWICTACKENEYVQDEFTCKACDLGWWPNADLTGCEPIPVRYLEWSNIESIIAIAFS
CLGILVTLFVTLIFVLYRDTPVVKSSSRELCYIILAGIFLGYVCPFTLIAKPTTTSCYLQ
RLLVGLSSAMCYSALVTKTNRIARILAGSKKKICTRKPRFMSAWAQVIIASILISVQLTL
VVTLIIMEPPMPILSYPSIKEVYLICNTSNLGVVAPLGYNGLLIMSCTYYAFKTRNVPAN
FNEAKYIAFTMYTTCIIWLAFVPIYFGSNYKIITTCFAVSLSVTVALGCMFTPKMYIIIA
KPERNVRSAFTTSDVVRMHVGDGKLPCRSNTFLNIFRRKKAGAGNANSNGKSVSWSEPGG
GQVPKGQHMWHRLSVHVKTNETACNQTAVIKPLTKSYQGSGKSLTFSDTSTKTLYNVEEE
EDAQPIRFSPPGSPSMVVHRRVPSAATTPPLPSHLTAEETPLFLAEPALPKGLPPPLQQQ
QQPPPQQKSLMDQLQGVVSNFSTAIPDFHAVLAGPGGPGNGLRSLYPPPPPPQHLQMLPL
QLSTFGEELVSPPADDDDDSERFKLLQEYVYEHEREGNTEEDELEEEEEDLQAASKLTPD
DSPALTPPSPFRDSVASGSSVPSSPVSESVLCTPPNVSYASVILRDYKQSSSTL

>hsa:2912
MGSLLALLALLLLWGAVAEGPAKKVLTLEGDLVLGGLFPVHQKGGPAEDCGPVNEHRGIQ
RLEAMLFALDRINRDPHLLPGVRLGAHILDSCSKDTHALEQALDFVRASLSRGADGSRHI
CPDGSYATHGDAPTAITGVIGGSYSDVSIQVANLLRLFQIPQISYASTSAKLSDKSRYDY
FARTVPPDFFQAKAMAEILRFFNWTYVSTVASEGDYGETGIEAFELEARARNICVATSEK
VGRAMSRAAFEGVVRALLQKPSARVAVLFTRSEDARELLAASQRLNASFTWVASDGWGAL
ESVVAGSEGAAEGAITIELASYPISDFASYFQSLDPWNNSRNPWFREFWEQRFRCSFRQR
DCAAHSLRAVPFEQESKIMFVVNAVYAMAHALHNMHRALCPNTTRLCDAMRPVNGRRLYK
DFVLNVKFDAPFRPADTHNEVRFDRFGDGIGRYNIFTYLRAGSGRYRYQKVGYWAEGLTL
DTSLIPWASPSAGPLPASRCSEPCLQNEVKSVQPGEVCCWLCIPCQPYEYRLDEFTCADC

GLGYWPNASLTGCFELPQEYIRWGDAWAVGPVTIACLGALATLFVLGVFVRHNATPVVKA
SGRELCYILLGGVFLCYCMTFIFIAKPSTAVCTLRRLGLGTAFSVCYSALLTKTNRIARI
FGGAREGAQRPRFISPASQVAICLALISGQLLIVVAWLVVEAPGTGKETAPERREVVTLR
CNHRDASMLGSLAYNVLLIALCTLYAFKTRKCPENFNEAKFIGFTMYTTCIIWLAFLPIF
YVTSSDYRVQTTTMCVSVSLSGSVVLGCLFAPKLHIILFQPQKNVVSHRAPTSRFGSAAA
RASSSLGQGSGSQFVPTVCNGREVVDSTTSSL

>hsa:2913
MKMLTRLQVLTLALFSKGFLLSLGDHNFLRREIKIEGDLVLGGLFPINEKGTGTEECGRI
NEDRGIQRLEAMLFAIDEINKDDYLLPGVKLGVHILDTCSRDTYALEQSLEFVRASLTKV
DEAEYMCPDGSYAIQENIPLLIAGVIGGSYSSVSIQVANLLRLFQIPQISYASTSAKLSD
KSRYDYFARTVPPDFYQAKAMAEILRFFNWTYVSTVASEGDYGETGIEAFEQEARLRNIC
IATAEKVGRSNIRKSYDSVIRELLQKPNARVVVLFMRSDDSRELIAAASRANASFTWVAS
DGWGAQESIIKGSEHVAYGAITLELASQPVRQFDRYFQSLNPYNNHRNPWFRDFWEQKFQ
CSLQNKRNHRRVCDKHLAIDSSNYEQESKIMFVVNAVYAMAHALHKMQRTLCPNTTKLCD
AMKILDGKKLYKDYLLKINFTAPFNPNKDADSIVKFDTFGDGMGRYNVFNFQNVGGKYSY
LKVGHWAETLSLDVNSIHWSRNSVPTSQCSDPCAPNEMKNMQPGDVCCWICIPCEPYEYL
ADEFTCMDCGSGQWPTADLTGCYDLPEDYIRWEDAWAIGPVTIACLGFMCTCMVVTVFIK
HNNTPLVKASGRELCYILLFGVGLSYCMTFFFIAKPSPVICALRRLGLGSSFAICYSALL
TKTNCIARIFDGVKNGAQRPKFISPSSQVFICLGLILVQIVMVSVWLILEAPGTRRYTLA
EKRETVILKCNVKDSSMLISLTYDVILVILCTVYAFKTRKCPENFNEAKFIGFTMYTTCI
IWLAFLPIFYVTSSDYRVQTTTMCISVSLSGFVVLGCLFAPKVHIILFQPQKNVVTHRLH
LNRFSVSGTGTTYSQSSASTYVPTVCNGREVLDSTTSSL

>hsa:2914
MPGKRGLGWWWARLPLCLLLSLYGPWMPSSLGKPKGHPHMNSIRIDGDITLGGLFPVHGR
GSEGKPCGELKKEKGIHRLEAMLFALDRINNDPDLLPNITLGARILDTCSRDTHALEQSL
TFVQALIEKDGTEVRCGSGGPPIITKPERVVGVIGASGSSVSIMVANILRLFKIPQISYA
STAPDLSDNSRYDFFSRVVPSDTYQAQAMVDIVRALKWNYVSTVASEGSYGESGVEAFIQ
KSREDGGVCIAQSVKIPREPKAGEFDKIIRRLLETSNARAVIIFANEDDIRRVLEAARRA
NQTGHFFWMGSDSWGSKIAPVLHLEEVAEGAVTILPKRMSVRGFDRYFSSRTLDNNRRNI
WFAEFWEDNFHCKLSRHALKKGSHVKKCTNRERIGQDSAYEQEGKVQFVIDAVYAMGHAL
HAMHRDLCPGRVGLCPRMDPVDGTQLLKYIRNVNFSGIAGNPVTFNENGDAPGRYDIYQY
QLRNDSAEYKVIGSWTDHLHLRIERMHWPGSGQQLPRSICSLPCQPGERKKTVKGMPCCW
HCEPCTGYQYQVDRYTCKTCPYDMRPTENRTGCRPIPIIKLEWGSPWAVLPLFLAVVGIA
ATLFVVITFVRYNDTPIVKASGRELSYVLLAGIFLCYATTFLMIAEPDLGTCSLRRIFLG
LGMSISYAALLTKTNRIYRIFEQGKRSVSAPRFISPASQLAITFSLISLQLLGICVWFVV
DPSHSVVDFQDQRTLDPRFARGVLKCDISDLSLICLLGYSMLLMVTCTVYAIKTRGVPET
FNEAKPIGFTMYTTCIVWLAFIPIFFGTSQSADKLYIQTTTLTVSVSLSASVSLGMLYMP
KVYIILFHPEQNVPKRKRSLKAVVTAATMSNKFTQKGNFRPNGEAKSELCENLEAPALAT
KQTYVTYTNHAI

>hsa:2915
MVLLLILSVLLLKEDVRGSAQSSERRVVAHMPGDIIIGALFSVHHQPTVDKVHERKCGAV

REQYGIQRVEAMLHTLERINSDPTLLPNITLGCEIRDSCWHSAVALEQSIEFIRDSLISS
EEEEGLVRCVDGSSSSFRSKKPIVGVIGPGSSSVAIQVQNLLQLFNIPQIAYSATSMDLS
DKTLFKYFMRVVPSDAQQARAMVDIVKRYNWTYVSAVHTEGNYGESGMEAFKDMSAKEGI
CIAHSYKIYSNAGEQSFDKLLKKLTSHLPKARVVACFCEGMTVRGLLMAMRRLGLAGEFL
LLGSDGWADRYDVTDGYQREAVGGITIKLQSPDVKWFDDYYLKLRPETNHRNPWFQEFWQ
HRFQCRLEGFPQENSKYNKTCNSSLTLKTHHVQDSKMGFVINAIYSMAYGLHNMQMSLCP
GYAGLCDAMKPIDGRKLLESLMKTNFTGVSGDTILFDENGDSPGRYEIMNFKEMGKDYFD
YINVGSWDNGELKMDDDEVWSKKSNIIRSVCSEPCEKGQIKVIRKGEVSCCWTCTPCKEN
EYVFDEYTCKACQLGSWPTDDLTGCDLIPVQYLRWGDPEPIAAVVFACLGLLATLFVTVV
FIIYRDTPVVKSSSRELCYIILAGICLGYLCTFCLIAKPKQIYCYLQRIGIGLSPAMSYS
ALVTKTNRIARILAGSKKKICTKKPRFMSACAQLVIAFILICIQLGIIVALFIMEPPDIM
HDYPSIREVYLICNTTNLGVVTPLGYNGLLILSCTFYAFKTRNVPANFNEAKYIAFTMYT
TCIIWLAFVPIYFGSNYKIITMCFSVSLSATVALGCMFVPKVYIILAKPERNVRSAFTTS
TVVRMHVGDGKSSSAASRSSSLVNLWKRRGSSGETLSSNGKSVTWAQNEKSSRGQHLWQR
LSIHINKKENPNQTAVIKPFPKSTESRGLGAGAGAGGSAGGVGATGGAGCAGAGPGGPES
PDAGPKALYDVAEAEEHFPAPARPRSPSPISTLSHRAGSASRTDDDVPSLHSEPVARSSS
SQGSLMEQISSVVTRFTANISELNSMMLSTAAPSPGVGAPLCSSYLIPKEIQLPTTMTTF
AEIQPLPAIEVTGGAQPAAGAQAAGDAARESPAAGPEAAAAKPDLEELVALTPPSPFRDS
VDSGSTTPNSPVSESALCIPSSPKYDTLIIRDYTQSSSSL

>hsa:2916
MARPRRAREPLLVALLPLAWLAQAGLARAAGSVRLAGGLTLGGLFPVHARGAAGRACGQL
KKEQGVHRLEAMLYALDRVNADPELLPGVRLGARLLDTCSRDTYALEQALSFVQALIRGR
GDGDEVGVRCPGGVPPLRPAPPERVVAVVGASASSVSIMVANVLRLFAIPQISYASTAPE
LSDSTRYDFFSRVVPPDSYQAQAMVDIVRALGWNYVSTLASEGNYGESGVEAFVQISREA
GGVCIAQSIKIPREPKPGEFSKVIRRLMETPNARGIIIFANEDDIRRVLEAARQANLTGH
FLWVGSDSWGAKTSPILSLEDVAVGAITILPKRASIDGFDQYFMTRSLENNRRNIWFAEF
WEENFNCKLTSSGTQSDDSTRKCTGEERIGRDSTYEQEGKVQFVIDAVYAIAHALHSMHQ
ALCPGHTGLCPAMEPTDGRMLLQYIRAVRFNGSAGTPVMFNENGDAPGRYDIFQYQATNG
SASSGGYQAVGQWAETLRLDVEALQWSGDPHEVPSSLCSLPCGPGERKKMVKGVPCCWHC
EACDGYRFQVDEFTCEACPGDMRPTPNHTGCRPTPVVRLSWSSPWAAPPLLLAVLGIVAT
TTVVATFVRYNNTPIVRASGRELSYVLLTGIFLIYAITFLMVAEPGAAVCAARRLFLGLG
TTLSYSALLTKTNRIYRIFEQGKRSVTPPPFISPTSQLVITFSLTSLQVVGMIAWLGARP
PHSVIDYEEQRTVDPEQARGVLKCDMSDLSLIGCLGYSLLLMVTCTVYAIKARGVPETFN
EAKPIGFTMYTTCIIWLAFVPIFFGTAQSAEKIYIQTTTLTVSLSLSASVSLGMLYVPKT
YVILFHPEQNVQKRKRSLKATSTVAAPPKGEDAEAHK

>hsa:2917
MVQLRKLLRVLTLMKFPCCVLEVLLCALAAAARGQEMYAPHSIRIEGDVTLGGLFPVHAK
GPSGVPCGDIKRENGIHRLEAMLYALDQINSDPNLLPNVTLGARILDTCSRDTYALEQSL
TFVQALIQKDTSDVRCTNGEPPVFVKPEKVVGVIGASGSSVSIMVANILRLFQIPQISYA
STAPELSDDRRYDFFSRVVPPDSFQAQAMVDIVKALGWNYVSTLASEGSYGEKGVESFTQ
ISKEAGGLCIAQSVRIPQERKDRTIDFDRIIKQLLDTPNSRAVVIFANDEDIKQILAAAK
RADQVGHFLWVGSDSWGSKINPLHQHEDIAEGAITIQPKRATVEGFDAYFTSRTLENNRR

NVWFAEYWEENFNCKLTISGSKKEDTDRKCTGQERIGKDSNYEQEGKVQFVIDAVYAMAH
ALHHMNKDLCADYRGVCPEMEQAGGKKLLKYIRNVNFNGSAGTPVMFNKNGDAPGRYDIF
QYQTTNTSNPGYRLIGQWTDELQLNIEDMQWGKGVREIPASVCTLPCKPGQRKKTQKGTP
CCWTCEPCDGYQYQFDEMTCQHCPYDQRPNENRTGCQDIPIIKLEWHSPWAVIPVFLAML
GIIATIFVMATFIRYNDTPIVRASGRELSYVLLTGIFLCYIITFLMIAKPDVAVCSFRRV
FLGLGMCISYAALLTKTNRIYRIFEQGKKSVTAPRLISPTSQLAITSSLISVQLLGVFIW
FGVDPPNIIIDYDEHKTMNPEQARGVLKCDITDLQIICSLGYSILLMVTCTVYAIKTRGV
PENFNEAKPIGFTMYTTCIVWLAFIPIFFGTAQSAEKLYIQTTTLTISMNLSASVALGML
YMPKVYIIIFHPELNVQKRKRSFKAVVTAATMSSRLSHKPSDRPNGEAKTELCENVDPNS
PAAKKKYVSYNNLVI

>hsa:2918
MVCEGKRSASCPCFFLLTAKFYWILTMMQRTHSQEYAHSIRVDGDIILGGLFPVHAKGER
GVPCGELKKEKGIHRLEAMLYAIDQINKDPDLLSNITLGVRILDTCSRDTYALEQSLTFV
QALIEKDASDVKCANGDPPIFTKPDKISGVIGAAASSVSIMVANILRLFKIPQISYASTA
PELSDNTRYDFFSRVVPPDSYQAQAMVDIVTALGWNYVSTLASEGNYGESGVEAFTQISR
EIGGVCIAQSQKIPREPRPGEFEKIIKRLLETPNARAVIMFANEDDIRRILEAAKKLNQS
GHFLWIGSDSWGSKIAPVYQQEEIAEGAVTILPKRASIDGFDRYFRSRTLANNRRNVWFA
EFWEENFGCKLGSHGKRNSHIKKCTGLERIARDSSYEQEGKVQFVIDAVYSMAYALHNMH
KDLCPGYIGLCPRMSTIDGKELLGYIRAVNFNGSAGTPVTFNENGDAPGRYDIFQYQITN
KSTEYKVIGHWTNQLHLKVEDMQWAHREHTHPASVCSLPCKPGERKKTVKGVPCCWHCER
CEGYNYQVDELSCELCPLDQRPNMNRTGCQLIPIIKLEWHSPWAVVPVFVAILGIIATTF
VIVTFVRYNDTPIVRASGRELSYVLLTGIFLCYSITFLMIAAPDTIICSFRRVFLGLGMC
FSYAALLTKTNRIHRIFEQGKKSVTAPKFISPASQLVITFSLISVQLLGVFVWFVVDPPH
IIIDYGEQRTLDPEKARGVLKCDISDLSLICSLGYSILLMVTCTVYAIKTRGVPETFNEA
KPIGFTMYTTCIIWLAFIPIFFGTAQSAEKMYIQTTTLTVSMSLSASVSLGMLYMPKVYI
IIFHPEQNVQKRKRSFKAVVTAATMQSKLIQKGNDRPNGEVKSELCESLETNTSSTKTTY
ISYSNHSI

>hsa:3269
MSLPNSSCLLEDKMCEGNKTTMASPQLMPLVVVLSTICLVTVGLNLLVLYAVRSERKLHT
VGNLYIVSLSVADLIVGAVVMPMNILYLLMSKWSLGRPLCLFWLSMDYVASTASIFSVFI
LCIDRYRSVQQPLRYLKYRTKTRASATILGAWFLSFLWVIPILGWNHFMQQTSVRREDKC
ETDFYDVTWFKVMTAIINFYLPTLLMLWFYAKIYKAVRQHCQHRELINRSLPSFSEIKLR
PENPKGDAKKPGKESPWEVLKRKPKDAGGGSVLKSPSQTPKEMKSPVVFSQEDDREVDKL
YCFPLDIVHMQAAAEGSSRDYVAVNRSHGQLKTDEQGLNTHGASEISEDQMLGDSQSFSR
TDSDTTTETAPGKGKLRSGSNTGLDYIKFTWKRLRSHSRQYVSGLHMNRERKAAKQLGFI
MAAFILCWIPYFIFFMVIAFCKNCCNEHLHMFTIWLGYINSTLNPLIYPLCNENFKKTFK
RILHIRS

>hsa:3274
MAPNGTASSFCLDSTACKITITVVLAVLILITVAGNVVVCLAVGLNRRLRNLTNCFIVSL
AITDLLLGLLVLPFSAIYQLSCKWSFGKVFCNIYTSLDVMLCTASILNLFMISLDRYCAV
MDPLRYPVLVTPVRVAISLVLIWVISITLSFLSIHLGWNSRNETSKGNHTTSKCKVQVNE

VYGLVDGLVTFYLPLLIMCITYYRIFKVARDQAKRINHISSWKAATIREHKATVTLAAVM
GAFIICWFPYFTAFVYRGLRGDDAINEVLEAIVLWLGYANSALNPILYAALNRDFRTGYQ
QLFCCRLANRNSHKTSLRSNASQLSRTQSREPRQQEEKPLKLQVWSGTEVTAPQGATDRP
WLCLPECWSVELTHSFIHLFIHSFANIHPIPTTCQEL

>hsa:3350
MDVLSPGQGNNTTSPPAPFETGGNTTGISDVTVSYQVITSLLLGTLIFCAVLGNACVVAA
IALERSLQNVANYLIGSLAVTDLMVSVLVLPMAALYQVLNKWTLGQVTCDLFIALDVLCC
TSSILHLCAIALDRYWAITDPIDYVNKRTPRRAAALISLTWLIGFLISIPPMLGWRTPED
RSDPDACTISKDHGYTIYSTFGAFYIPLLLMLVLYGRIFRAARFRIRKTVKKVEKTGADT
RHGASPAPQPKKSVNGESGSRNWRLGVESKAGGALCANGAVRQGDDGAALEVIEVHRVGN
SKEHLPLPSEAGPTPCAPASFERKNERNAEAKRKMALARERKTVKTLGIIMGTFILCWLP
FFIVALVLPFCESSCHMPTLLGAIINWLGYSNSLLNPVIYAYFNKDFQNAFKKIIKCKFC
RQ

>hsa:3351
MEEPGAQCAPPPPAGSETWVPQANLSSAPSQNCSAKDYIYQDSISLPWKVLLVMLLALIT
LATTLSNAFVIATVYRTRKLHTPANYLIASLAVTDLLVSILVMPISTMYTVTGRWTLGQV
VCDFWLSSDITCCTASILHLCVIALDRYWAITDAVEYSAKRTPKRAAVMIALVWVFSISI
SLPPFFWRQAKAEEEVSECVVNTDHILYTVYSTVGAFYFPTLLLIALYGRIYVEARSRIL
KQTPNRTGKRLTRAQLITDSPGSTSSVTSINSRVPDVPSESGSPVYVNQVKVRVSDALLE
KKKLMAARERKATKTLGIILGAFIVCWLPFFIISLVMPICKDACWFHLAIFDFFTWLGYL
NSLINPIIYTMSNEDFKQAFHKLIRFKCTS

>hsa:3352
MSPLNQSAEGLPQEASNRSLNATETSEAWDPRTLQALKISLAVVLSVITLATVLSNAFVL
TTILLTRKLHTPANYLIGSLATTDLLVSILVMPISIAYTITHTWNFGQILCDIWLSSDIT
CCTASILHLCVIALDRYWAITDALEYSKRRTAGHAATMIAIVWAISICISIPPLFWRQAK
AQEEMSDCLVNTSQISYTIYSTCGAFYIPSVLLIIILYGRIYRAARNRILNPPSLYGKRFT
TAHLITGSAGSSLCSLNSSLHEGHSHSAGSPLFFNHVKIKLADSALERKRISAARERKAT
KILGIILGAFIICWLPFFVVSLVLPICRDSCWIHPALFDFFTWLGYLNSLINPIIYTVFN
EEFRQAFQKIVPFRKAS

>hsa:3354
MNITNCTTEASMAIRPKTITEKMLICMTLVVITTLTTLLNLAVIMAIGTTKKLHQPANYL
ICSLAVTDLLVAVLVMPLSIIYIVMDRWKLGYFLCEVWLSVDMTCCTCSILHLCVIALDR
YWAITNAIEYARKRTAKRAALMILTVWTISIFISMPPLFWRSHRRLSPPPSQCTIQHDHV
IYTIYSTLGAFYIPLTLILILYYRIYHAAKSLYQKRGSSRHLSNRSTDSQNSFASCKLTQ
TFCVSDFSTSDPTTEFEKFHASIRIPPFDNDLDHPGERQQISSTRERKAARILGLILGAF
ILSWLPFFIKELIVGLSIYTVSSEVADFLTWLGYVNSLINPLLYTSFNEDFKLAFKKLIR
CREHT

>hsa:3355
MDFLNSSDQNLTSEELLNRMPSKILVSLTLSGLALMTTTINSLVIAAIIVTRKLHHPANY

LICSLAVTDFLVAVLVMPFSIVYIVRESWIMGQVVCDIWLSVDITCCTCSILHLSAIALD
RYRAITDAVEYARKRTPKHAGIMITIVWIISVFISMPPLFWRHQGTSRDDECIIKHDHIV
STIYSTFGAFYIPLALILILYYKIYRAAKTLYHKRQASRIAKEEVNGQVLLESGEKSTKS
VSTSYVLEKSLSDPSTDFDKIHSTVRSLRSEFKHEKSWRRQKISGTRERKAATTLGLILG
AFVICWLPFFVKELVVNVCDKCKISEEMSNFLAWLGYLNSLINPLIYTIFNEDFKKAFQK
LVRCRC

>hsa:3356
MDILCEENTSLSSTTNSLMQLNDDTRLYSNDFNSGEANTSDAFNWTVDSENRTNLSCEGC
LSPSCLSLLHLQEKNWSALLTAVVIILTIAGNILVIMAVSLEKKLQNATNYFLMSLAIAD
MLLGFLVMPVSMLTILYGYRWPLPSKLCAVWIYLDVLFSTASIMHLCAISLDRYVAIQNP
IHHSRFNSRTKAFLKIIAVWTISVGISMPIPVFGLQDDSKVFKEGSCLLADDNFVLIGSF
VSFFIPLTIMVITYFLTIKSLQKEATLCVSDLGTRAKLASFSFLPQSSLSSEKLFQRSIH
REPGSYTGRRTMQSISNEQKACKVLGIVFFLFVVMWCPFFITNIMAVICKESCNEDVIGA
LLNVFVWIGYLSSAVNPLVYTLFNKTYRSAFSRYIQCQYKENKKPLQLILVNTIPALAYK
SSQLQMGQKKNSKQDAKTTDNDCSMVALGKQHSEEASKDNSDGVNEKVSCV

>hsa:3357
MALSYRVSELQSTIPEHILQSTFVHVISSNWSGLQTESIPEEMKQIVEEQGNKLHWAALL
ILMVIIPTIGGNTLVILAVSLEKKLQYATNYFLMSLAVADLLVGLFVMPIALLTIMFEAM
WPLPLVLCPAWLFLDVLFSTASIMHLCAISVDRYIAIKKPIQANQYNSRATAFIKITVVW
LISIGIAIPVPIKGIETDVDNPNNITCVLTKERFGDFMLFGSLAAFFTPLAIMIVTYFLT
IHALQKKAYLVKNKPPQRLTWLTVSTVFQRDETPCSSPEKVAMLDGSRKDKALPNSGDET
LMRRTSTIGKKSVQTISNEQRASKVLGIVFFLFLLMWCPFFITNITLVLCDSCNQTTLQM
LLEIFVWIGYVSSGVNPLVYTLFNKTFRDAFGRYITCNYRATKSVKTLRKRSSKIYFRNP
MAENSKFFKKHGIRNGINPAMYQSPMRLRSSTIQSSSIILLDTLLLTENEGDKTEEQVSY
V

>hsa:3358
MVNLRNAVHSFLVHLIGLLVWQCDISVSPVAAIVTDIFNTSDGGRFKFPDGVQNWPALSI
VIIIIMTIGGNILVIMAVSMEKKLHNATNYFLMSLAIADMLVGLLVMPLSLLAILYDYVW
PLPRYLCPVWISLDVLFSTASIMHLCAISLDRYVAIRNPIEHSRFNSRTKAIMKIAIVWA
ISIGVSVPIPVIGLRDEEKVFVNNTTCVLNDPNFVLIGSFVAFFIPLTIMVITYCLTIYV
LRRQALMLLHGHTEEPPGLSLDFLKCCKRNTAEEENSANPNQDQNARRRKKKERRPRGTM
QAINNERKASKVLGIVFFVFLIMWCPFFITNILSVLCEKSCNQKLMEKLLNVFVWIGYVC
SGINPLVYTLFNKIYRRAFSNYLRCNYKVEKKPPVRQIPRVAATALSGRELNVNIYRHTN
EPVIEKASDNEPGIEMQVENLELPVNPSSVVSERISSV

>hsa:3360
MDKLDANVSSEEGFGSVEKVVLLTFLSTVILMAILGNLLVMVAVCWDRQLRKIKTNYFIV
SLAFADLLVSVLVMPFGAIELVQDIWIYGEVFCLVRTSLDVLLTTASIFHLCCISLDRYY
AICCQPLVYRNKMTPLRIALMLGGCWVIPTFISFLPIMQGWNNIGIIDLIEKRKFNQNSN
STYCVFMVNKPYAITCSVVAFYIPFLLMVLAYYRIYVTAKEHAHQIQMLQRAGASSESRP
QSADQHSTHRMRTETKAAKTLCIIMGCFCLCWAPFFVTNIVDPFIDYTVPGQVWTAFLWL

GYINSGLNPFLYAFLNKSFRRAFLIILCCDDERYRRPSILGQTVPCSTTTINGSTHVLRD
AVECGGQWESQCHPPATSPLVAAQPSDT

>hsa:3361
MDLPVNLTSFSLSTPSPLETNHSLGKDDLRPSSPLLSVFGVLILTLLGFLVAATFAWNLL
VLATILRVRTFHRVPHNLVASMAVSDVLVAALVMPLSLVHELSGRRWQLGRRLCQLWIAC
DVLCCTASIWNVTAIALDRYWSITRHMEYTLRTRKCVSNVMIALTWALSAVISLAPLLFG
WGETYSEGSEECQVSREPSYAVFSTVGAFYLPLCVVLFVYWKIYKAAKFRVGSRKTNSVS
PISEAVEVKDSAKQPQMVFTVRHATVTFQPEGDTWREQKEQRAALMVGILIGVFVLCWIP
FFLTELISPLCSCDIPAIWKSIFLWLGYSNSFFNPLIYTAFNKNYNSAFKNFFSRQH

>hsa:3362
MVPEPGPTANSTPAWGAGPPSAPGGSGWVAAALCVVIALTAAANSLLIALICTQPALRNT
SNFFLVSLFTSDLMVGLVVMPPAMLNALYGRWVLARGLCLLWTAFDVMCCSASILNLCLI
SLDRYLLILSPLRYKLRMTPLRALALVLGAWSLAALASFLPLLLGWHELGHARPPVPGQC
RLLASLPFVLVASGLTFFLPSGAICFTYCRILLAARKQAVQVASLTTGMASQASETLQVP
RTPRPGVESADSRRLATKHSRKALKASLTLGILLGMFFVTWLPFFVANIVQAVCDCISPG
LFDVLTWLGYCNSTMNPIIYPLFMRDFKRALGRFLPCPRCPRERQASLASPSLRTSHSGP
RPGLSLQQVLPLPLPPDSDSDSDAGSGGSSGLRLTAQLLLPGEATQDPPLPTRAAAAVNF
FNIDPAEPELRPHPLGIPTN

>hsa:3363
MMDVNSSGRPDLYGHLRSFLLPEVGRGLPDLSPDGGADPVAGSWAPHLLSEVTASPAPTW
DAPPDNASGCGEQINYGRVEKVVIGSILTLITLLTIAGNCLVVISVCFVKKLRQPSNYLI
VSLALADLSVAVAVMPFVSVTDLIGGKWIFGHFFCNVFIAMDVMCCTASIMTLCVISIDR
YLGITRPLTYPVRQNGKCMAKMILSVWLLSASITLPPLFGWAQNVNDDKVCLISQDFGYT
IYSTAVAFYIPMSVMLFMYYQIYKAARKSAAKHKFPGFPRVEPDSVIALNGIVKLQKEVE
ECANLSRLLKHERKNISIFKREQKAATTLGIIVGAFTVCWLPFFLLSTARPFICGTSCSC
IPLWVERTFLWLGYANSLINPFIYAFFNRDLRTTYRSLLQCQYRNINRKLSAAGMHEALK
LAERPERPEFVLQNADYCRKKGHDS

>hsa:338442
MNRHHLQDHFLEIDKKNCCVFRDDFIVKVLPPVLGLEFIFGLLGNGLALWIFCFHLKSWK
SSRIFLFNLAVADFLLIICLPFLMDNYVRRWDWKFGDIPCRLMLFMLAMNRQGSIIFLTV
VAVDRYFRVVHPHHALNKISNRTAAIISCLLWGITIGLTVHLLKKKMPIQNGGANLCSSF
SICHTFQWHEAMFLLEFFLPLGIILFCSARIIWSLRQRQMDRHAKIKRAITFIMVVAIVF
VICFLPSVVVRIRIFWLLHTSGTQNCEVYRSVDLAFFITLSFTYMNSMLDPVVYYFSSPS
FPNFFSTLINRCLQRKMTGEPDNNRSTSVELTGDPNKTRGAPEALMANSGEPWSPSYLGP
TSP

>hsa:3577
MSNITDPQMWDFDDLNFTGMPPADEDYSPCMLETETLNKYVVIIAYALVFLLSLLGNSLV
MLVILYSRVGRSVTDVYLLNLALADLLFALTLPIWAASKVNGWIFGTFLCKVVSLLKEVN
FYSGILLLACISVDRYLAIVHATRTLTQKRHLVKFVCLGCWGLSMNLSLPFFLFRQAYHP

NNSSPVCYEVLGNDTAKWRMVLRILPHTFGFIVPLFVMLFCYGFTLRTLFKAHMGQKHRA
MRVIFAVVLIFLLCWLPYNLVLLADTLMRTQVIQESCERRNNIGRALDATEILGFLHSCL
NPIIYAFIGQNFRHGFLKILAMHGLVSKEFLARHRVTSYTSSSVNVSSNL
>hsa:4543
MQGNGSALPNASQPVLRGDGARPSWLASALACVLIFTIVVDILGNLLVILSVYRNKKLRN
AGNIFVVSLAVADLVVAIYPYPLVLMSIFNNGWNLGYLHCQVSGFLMGLSVIGSIFNITG
IAINRYCYICHSLKYDKLYSSKNSLCYVLLIWLLTLAAVLPNLRAGTLQYDPRIYSCTFA
QSVSSAYTIAVVVFHFLVPMIIVIFCYLRIWILVLQVRQRVKPDRKPKLKPQDFRNFVTM
FVVFVLFAICWAPLNFIGLAVASDPASMVPRIPEWLFVASYYMAYFNSCLNAIIYGLLNQ
NFRKEYRRIIVSLCTARVFFVDSSNDVADRVKWKPSPLMTNNNVVKVDSV

>hsa:4985
MEPAPSAGAELQPPLFANASDAYPSACPSAGANASGPPGARSASSLALAIAITALYSAVC
AVGLLGNVLVMFGIVRYTKMKTATNIYIFNLALADALATSTLPFQSAKYLMETWPFGELL
CKAVLSIDYYNMFTSIFTLTMMSVDRYIAVCHPVKALDFRTPAKAKLINICIWVLASGVG
VPIMVMAVTRPRDGAVVCMLQFPSPSWYWDTVTKICVFLFAFVVPILIITVCYGLMLLRL
RSVRLLSGSKEKDRSLRRITRMVLVVVGAFVVCWAPIHIFVIVWTLVDIDRRDPLVVAAL
HLCIALGYANSSLNPVLYAFLDENFKRCFRQLCRKPCGRPDPSSFSRAREATARERVTAC
TPSDGPGGGAAA

>hsa:4986
MDSPIQIFRGEPGPTCAPSACLPPNSSAWFPGWAEPDSNGSAGSEDAQLEPAHISPAIPV
IITAVYSVVFVVGLVGNSLVMFVIIRYTKMKTATNIYIFNLALADALVTTTMPFQSTVYL
MNSWPFGDVLCKIVISIDYYNMFTSIFTLTMMSVDRYIAVCHPVKALDFRTPLKAKIINI
CIWLLSSSVGISAIVLGGTKVREDVDVIECSLQFPDDDYSWWDLFMKICVFIFAFVIPVL
IIIVCYTLMILRLKSVRLLSGSREKDRNLRRITRLVLVVVAVFVVCWTPIHIFILVEALG
STSHSTAALSSYYFCIALGYTNSSLNPILYAFLDENFKRCFRDFCFPLKMRMERQSTSRV
RNTVQDPAYLRDIDGMNKPV

>hsa:4988
MDSSAAPTNASNCTDALAYSSCSPAPSPGSWVNLSHLDGNLSDPCGPNRTDLGGRDSLCP
PTGSPSMITAITIMALYSIVCVVGLFGNFLVMYVIVRYTKMKTATNIYIFNLALADALAT
STLPFQSVNYLMGTWPFGTILCKIVISIDYYNMFTSIFTLCTMSVDRYIAVCHPVKALDF
RTPRNAKIINVCNWILSSAIGLPVMFMATTKYRQGSIDCTLTFSHPTWYWENLLKICVFI
FAFIMPVLIITVCYGLMILRLKSVRMLSGSKEKDRNLRRITRMVLVVVAVFIVCWTPIHI
YVIIKALVTIPETTFQTVSWHFCIALGYTNSCLNPVLYAFLDENFKRCFREFCIPTSSNI
EQQNSTRIRQNTRDHPSTANTVDRTNHQLENLEAETAPLP

>hsa:5028
MTEVLWPAVPNGTDAAFLAGPGSSWGNSTVASTAAVSSSFKCALTKTGFQFYYLPAVYIL
VFIIGFLGNSVAIWMFVFHMKPWSGISVYMFNLALADFLYVLTLPALIFYYFNKTDWIFG
DAMCKLQRFIFHVNLYGSILFLTCISAHRYSGVVYPLKSLGRLKKKNAICISVLVWLIVV
VAISPILFYSGTGVRKNKTITCYDTTSDEYLRSYFIYSMCTTVAMFCVPLVLILGCYGLI
VRALIYKDLDNSPLRRKSIYLVIIVLTVFAVSYIPFHVMKTMNLRARLDFQTPAMCAFND

RVYATYQVTRGLASLNSCVDPILYFLAGDTFRRRLSRATRKASRRSEANLQSKSEDMTLN
ILPEFKQNGDTSL

>hsa:5029
MAADLGPWNDTINGTWDGDELGYRCRFNEDFKYVLLPVSYGVVCVPGLCLNAVALYIFLC
RLKTWNASTTYMFHLAVSDALYAASLPLLVYYYARGDHWPFSTVLCKLVRFLFYTNLYCS
ILFLTCISVHRCLGVLRPLRSLRWGRARYARRVAGAVWVLVLACQAPVLYFVTTSARGGR
VTCHDTSAPELFSRFVAYSSVMLGLLFAVPFAVILVCYVLMARRLLKPAYGTSGGLPRAK
RKSVRTIAVVLAVFALCFLPFHVTRTLYYSFRSLDLSCHTLNAINMAYKVTRPLASANSC
LDPVLYFLAGQRLVRFARDAKPPTGPSPATPARRRLGLRRSDRTDMQRIEDVLGSSEDSR
RTESTPAGSENTKDIRL

>hsa:5030
MASTESSLLRSLGLSPGPGSSEVELDCWFDEDFKFILLPVSYAVVFVLGLGLNAPTLWLF
IFRLRPWDATATYMFHLALSDTLYVLSLPTLIYYYAAHNHWPFGTEICKFVRFLFYWNLY
CSVLFLTCISVHRYLGICHPLRALRWGRPRLAGLLCLAVWLVVAGCLVPNLFFVTTSNKG
TTVLCHDTTRPEEFDHYVHFSSAVMGLLFGVPCLVTLVCYGLMARRLYQPLPGSAQSSSR
LRSLRTIAVVLTVFAVCFVPFHITRTIYYLARLLEADCRVLNIVNVVYKVTRPLASANSC
LDPVLYLLTGDKYRRQLRQLCGGGKPQPRTAASSLALVSLPEDSSCRWAATPQDSSCSTP
RADRL

>hsa:5031
MEWDNGTGQALGLPPTTCVYRENFKQLLLPPVYSAVLAAGLPLNICVITQICTSRRALTR
TAVYTLNLALADLLYACSLPLLIYNYAQGDHWPFGDFACRLVRFLFYANLHGSILFLTCI
SFQRYLGICHPLAPWHKRGGRRAAWLVCVAVWLAVTTQCLPTAIFAATGIQRNRTVCYDL
SPPALATHYMPYGMALTVIGFLLPFAALLACYCLLACRLCRQDGPAEPVAQERRGKAARM
AVVVAAAFAISFLPFHITKTAYLAVRSTPGVPCTVLEAFAAAYKGTRPFASANSVLDPIL
FYFTQKKFRRRPHELLQKLTAKWQRQGR

>hsa:5032
MAANVSGAKSCPANFLAAADDKLSGFQGDFLWPILVVEFLVAVASNGLALYRFSIRKQRP
WHPAVVFSVQLAVSDLLCALTLPPLAAYLYPPKHWRYGEAACRLERFLFTCNLLGSVIFI
TCISLNRYLGIVHPFFARSHLRPKHAWAVSAAGWVLAALLAMPTLSFSHLKRPQQGAGNC
SVARPEACIKCLGTADHGLAAYRAYSLVLAGLGCGLPLLLTLAAYGALGRAVLRSPGMTV
AEKLRVAALVASGVALYASSYVPYHIMRVLNVDARRRWSTRCPSFADIAQATAALELGPY
VGYQVMRGLMPLAFCVHPLLYMAAVPSLGCCCRHCPGYRDSWNPEDAKSTGQALPLNATA
APKPSEPQSRELSQ

>hsa:552
MRLSAGPDAGPSGNSSPWWPLATGAGNTSREAEALGEGNGPPRDVRNEELAKLEIAVLAV
TFAVAVLGNSSVLLALHRTPRKTSRMHLFIRHLSLADLAVAFFQVLPQMCWDITYRFRGP
DWLCRVVKHLQVFGMFASAYMLVVMTADRYIAVCHPLKTLQQPARRSRLMIAAAWVLSFV
LSTPQYFVFSMIEVNNVTKARDCWATFIQPWGSRAYVTWMTGGIFVAPVVILGTCYGFIC
YNIWCNVRGKTASRQSKGAEQAGVAFQKGFLLAPCVSSVKSISRAKIRTVKMTFVIVTAY

IVCWAPFFIIQMWSVWDPMSVWTESENPTITITALLGSLNSCCNPWIYMFFSGHLLQDCV
QSFPCCQNMKEKFNKEDTDSMSRRQTFYSNNRSPTNSTGMWKDSPKSSKSIKFIPVST

>hsa:554
MLMASTTSAVPGHPSLPSLPSNSSQERPLDTRDPLLARAELALLSIVFVAVALSNGLVLA
ALARRGRRGHWAPIHVFIGHLCLADLAVALFQVLPQLAWKATDRFRGPDALCRAVKYLQM
VGMYASSYMILAMTLDRHRAICRPMLAYRHGSGAHWNRPVLVAWAFSLLLSLPQLFIFAQ
RNVEGGSGVTDCWACFAEPWGRRTYVTWIALMVFVAPTLGIAACQVLIFREIHASLVPGP
SERPGGRRRGRRTGSPGEGAHVSAAVAKTVRMTLVIVVVYVLCWAPFFLVQLWAAWDPEA
PLEGAPFVLLMLLASLNSCTNPWIYASFSSSVSSELRSLLCCARGRTPPSLGPQDESCTT
ASSSLAKDTSS

>hsa:56413
MSVCYRPPGNETLLSWKTSRATGTAFLLLAALLGLPGNGFVVWSLAGWRPARGRPLAATL
VLHLALADGAVLLLTPLFVAFLTRQAWPLGQAGCKAVYYVCALSMYASVLLTGLLSLQRC
LAVTRPFLAPRLRSPALARRLLLAVWLAALLLAVPAAVYRHLWRDRVCQLCHPSPVHAAA
HLSLETLTAFVLPFGLMLGCYSVTLARLRGARWGSGRHGARVGRLVSAIVLAFGLLWAPY
HAVNLLQAVAALAPPEGALAKLGGAGQAARAGTTALAFFSSSVNPVLYVFTAGDLLPRAG
PRFLTRLFEGSGEARGGGRSREGTMELRTTPQLKVVGQGRGNGDPGGGMEKDGPEWDL

>hsa:57105
MERKFMSLQPSISVSEMEPNGTFSNNNSRNCTIENFKREFFPIVYLIIFFWGVLGNGLSI
YVFLQPYKKSTSVNVFMLNLAISDLLFISTLPFRADYYLRGSNWIFGDLACRIMSYSLYV
NMYSSIYFLTVLSVVRFLAMVHPFRLLHVTSIRSAWILCGIIWILIMASSIMLLDSGSEQ
NGSVTSCLELNLYKIAKLQTMNYIALVVGCLLPFFTLSICYLLIIRVLLKVEVPESGLRV
SHRKALTTIIITLIIFFLCFLPYHTLRTVHLTTWKVGLCKDRLHKALVITLALAAANACF
NPLLYYFAGENFKDRLKSALRKGHPQKAKTKCVFPVSVWLRKETRV

>hsa:5724
MEPHDSSHMDSEFRYTLFPIVYSIIFVLGVIANGYVLWVFARLYPCKKFNEIKIFMVNLT
MADMLFLITLPLWIVYYQNQGNWILPKFLCNVAGCLFFINTYCSVAFLGVITYNRFQAVT
RPIKTAQANTRKRGISLSLVIWVAIVGAASYFLILDSTNTVPDSAGSGNVTRCFEHYEKG
SVPVLIIHIFIVFSFFLVFLIILFCNLVIIRTLLMQPVQQQRNAEVKRRALWMVCTVLAV
FIICFVPHHVVQLPWTLAELGFQDSKFHQAINDAHQVTLCLLSTNCVLDPVIYCFLTKKF
RKHLTEKFYSMRSSRKCSRATTDTVTEVVVPFNQIPGNSLKN

>hsa:5731
MSPCGPLNLSLAGEATTCAAPWVPNTSAVPPSGASPALPIFSMTLGAVSNLLALALLAQA
AGRLRRRRSAATFLLFVASLLATDLAGHVIPGALVLRLYTAGRAPAGGACHFLGGCMVFF
GLCPLLLGCGMAVERCVGVTRPLLHAARVSVARARLALAAVAAVALAVALLPLARVGRYE
LQYPGTWCFIGLGPPGGWRQALLAGLFASLGLVALLAALVCNTLSGLALLRARWRRRSRR
PPPASGPDSRRRWGAHGPRSASASSASSIASASTFFGGSRSSGSARRARAHDVEMVGQLV
GIMVVSCICWSPMLVLVALAVGGWSSTSLQRPLFLAVRLASWNQILDPWVYILLRQAVLR
QLLRLLPPRAGAKGGPAGLGLTPSAWEASSLRSSRHSGLSHF

>hsa:5732
MGNASNDSQSEDCETRQWLPPGESPAISSVMFSAGVLGNLIALALLARRWRGDVGCSAGR
RSSLSLFHVLVTELVFTDLLGTCLISPVVLASYARNQTLVALAPESRACTYFAFAMTFFS
LATMLMLFAMALERYLSIGHPYFYQRRVSRSGGLAVLPVIYAVSLLFCSLPLLDYGQYVQ
YCPGTWCFIRHGRTAYLQLYATLLLLLIVSVLACNFSVILNLIRMHRRSRRSRCGPSLGS
GRGGPGARRRGERVSMAEETDHLILLAIMTITFAVCSLPFTIFAYMNETSSRKEKWDLQA
LRFLSINSIIDPWVFAILRPPVLRLMRSVLCCRISLRTQDATQTSCSTQSDASKQADL

>hsa:5733
MKETRGYGGDAPFCTRLNHSYTGMWAPERSAEARGNLTRPPGSGEDCGSVSVAFPITMLL
TGFVGNALAMLLVSRSYRRRESKRKKSFLLCIGWLALTDLVGQLLTTPVVIVVYLSKQRW
EHIDPSGRLCTFFGLTMTVFGLSSLFIASAMAVERALAIRAPHWYASHMKTRATRAVLLG
VWLAVLAFALLPVLGVGQYTVQWPGTWCFISTGRGGNGTSSSHNWGNLFFASAFAFLGLL
ALTVTFSCNLATIKALVSRCRAKATASQSSAQWGRITTETAIQLMGIMCVLSVCWSPLLI
MMLKMIFNQTSVEHCKTHTEKQKECNFFLIAVRLASLNQILDPWVYLLLRKILLRKFCQI
RYHTNNYASSSTSLPCQCSSTLMWSDHLER

>hsa:5737
MSMNNSKQLVSPAAALLSNTTCQTENRLSVFFSVIFMTVGILSNSLAIAILMKAYQRFRQ
KSKASFLLLASGLVITDFFGHLINGAIAVFVYASDKEWIRFDQSNVLCSIFGICMVFSGL
CPLLLGSVMAIERCIGVTKPIFHSTKITSKHVKMMLSGVCLFAVFIALLPILGHRDYKIQ
ASRTWCFYNTEDIKDWEDRFYLLLFSFLGLLALGVSLLCNAITGITLLRVKFKSQQHRQG
RSHHLEMVIQLLAIMCVSCICWSPFLVTMANIGINGNHSLETCETTLFALRMATWNQILD
PWVYILLRKAVLKNLYKLASQCCGVHVISLHIWELSSIKNSLKVAAISESPVAEKSAST

>hsa:5739
MADSCRNLTYVRGSVGPATSTLMFVAGVVGNGLALGILSARRPARPSAFAVLVTGLAATD
LLGTSFLSPAVFVAYARNSSLLGLARGGPALCDAFAFAMTFFGLASMLILFAMAVERCLA
LSHPYLYAQLDGPRCARLALPAIYAFCVLFCALPLLGLGQHQQYCPGSWCFLRMRWAQPG
GAAFSLAYAGLVALLVAAIFLCNGSVTLSLCRMYRQQKRHQGSLGPRPRTGEDEVDHLIL
LALMTVVMAVCSLPLTIRCFTQAVAPDSSSEMGDLLAFRFYAFNPILDPWVFILFRKAVF
QRLKLWVCCLCLGPAHGDSQTPLSQLASGRRDPRAPSAPVGKEGSCVPLSAWGEGQVEPL
PPTQQSSGSAVGTSSKAEASVACSLC

>hsa:59340
MPDTNSTINLSLSTRVTLAFFMSLVAFAIMLGNALVILAFVVDKNLRHRSSYFFLNLAIS
DFFVVSESWKDEGSECEPGFFSEWYILAITSFLEFVIPVILVAYFNMNIYWSLWKRDHLS
RCQSHPGLTAVSSNICGHSFRGRLSSRRSLSASTEVPASFHSERQRRKSSLMFSSRTKMN
SNTIASKMGSFSQSDSVALHQREHVELLRARRLAKSLAILLGVFAVCWAPYSLFTIVLSF
YSSATGPKSVWYRIAFWLQWFNSFVNPLLYPLCHKRFQKAFLKIFCIKKQPLPSQHSRSV
SS

>hsa:6010

MNGTEGPNFYVPFSNATGVVRSPFEYPQYYLAEPWQFSMLAAYMFLLIVLGFPINFLTLY
VTVQHKKLRTPLNYILLNLAVADLFMVLGGFTSTLYTSLHGYFVFGPTGCNLEGFFATLG
GEIALWSLVVLAIERYVVVCKPMSNFRFGENHAIMGVAFTWVMALACAAPPLAGWSRYIP
EGLQCSCGIDYYTLKPEVNNESFVIYMFVVHFTIPMIIIFFCYGQLVFTVKEAAAQQQES
ATTQKAEKEVTRMVIIMVIAFLICWVPYASVAFYIFTHQGSNFGPIFMTIPAFFAKSAAI
YNPVIYIMMNKQFRNCMLTTICCGKNPLGDDEASATVSKTETSQVAPA

>hsa:64805
MQAVDNLTSAPGNTSLCTRDYKITQVLFPLLYTVLFFVGLITNGLAMRIFFQIRSKSNFI
IFLKNTVISDLLMILTFPFKILSDAKLGTGPLRTFVCQVTSVIFYFTMYISISFLGLITI
DRYQKTTRPFKTSNPKNLLGAKILSVVIWAFMFLLSLPNMILTNRQPRDKNVKKCSFLKS
EFGLVWHEIVNYICQVIFWINFLIVIVCYTLITKELYRSYVRTRGVGKVPRKKVNVKVFI
IIAVFFICFVPFHFARIPYTLSQTRDVFDCTAENTLFYVKESTLWLTSLNACLDPFIYFF
LCKSFRNSLISMLKCPNSATSLSQDNRKKEQDGGDPNEETPM

>hsa:6751
MFPNGTASSPSSSPSPSPGSCGEGGGSRGPGAGAADGMEEPGRNASQNGTLSEGQGSAIL
ISFIYSVVCLVGLCGNSMVIYVILRYAKMKTATNIYILNLAIADELLMLSVPFLVTSTLL
RHWPFGALLCRLVLSVDAVNMFTSIYCLTVLSVDRYVAVVHPIKAARYRRPTVAKVVNLG
VWVLSLLVILPIVVFSRTAANSDGTVACNMLMPEPAQRWLVGFVLYTFLMGFLLPVGAIC
LCYVLIIAKMRMVALKAGWQQRKRSERKITLMVMMVVMVFVICWMPFYVVQLVNVFAEQD
DATVSQLSVILGYANSCANPILYGFLSDNFKRSFQRILCLSWMDNAAEEPVDYYATALKS
RAYSVEDFQPENLESGGVFRNGTCTSRITTL

>hsa:6752
MDMADEPLNGSHTWLSIPFDLNGSVVSTNTSNQTEPYYDLTSNAVLTFIYFVVCIIGLCG
NTLVIYVILRYAKMKTITNIYILNLAIADELFMLGLPFLAMQVALVHWPFGKAICRVVMT
VDGINQFTSIFCLTVMSIDRYLAVVHPIKSAKWRRPRTAKMITMAVWGVSLLVILPIMIY
AGLRSNQWGRSSCTINWPGESGAWYTGFIIYTFILGFLVPLTIICLCYLFIIIKVKSSGI
RVGSSKRKKSEKKVTRMVSIVVAVFIFCWLPFYIFNVSSVSMAISPTPALKGMFDFVVVL
TYANSCANPILYAFLSDNFKKSFQNVLCLVKVSGTDDGERSDSKQDKSRLNETTETQRTL
LNGDLQTSI

>hsa:6753
MDMLHPSSVSTTSEPENASSAWPPDATLGNVSAGPSPAGLAVSGVLIPLVYLVVCVVGLL
GNSLVIYVVLRHTASPSVTNVYILNLALADELFMLGLPFLAAQNALSYWPFGSLMCRLVM
AVDGINQFTSIFCLTVMSVDRYLAVVHPTRSARWRTAPVARTVSAAVWVASAVVVLPVVV
FSGVPRGMSTCHMQWPEPAAAWRAGFIIYTAALGFFGPLLVICLCYLLIVVKVRSAGRRV
WAPSCQRRRRSERRVTRMVVAVVALFVLCWMPFYVLNIVNVVCPLPEEPAFFGLYFLVVA
LPYANSCANPILYGFLSYRFKQGFRRVLLRPSRRVRSQEPTVGPPEKTEEEDEEEEDGEE
SREGGKGKEMNGRVSQITQPGTSGQERPPSRVASKEQQLLPQEASTGEKSSTMRISYL

>hsa:6755
MEPLFPASTPSWNASSPGAASGGGDNRTLVGPAPSAGARAVLVPVLYLLVCAAGLGGNTL

VIYVVLRFAKMKTVTNIYILNLAVADVLYMLGLPFLATQNAASFWPFGPVLCRLVMTLDG
VNQFTSVFCLTVMSVDRYLAVVHPLSSARWRRPRVAKLASAAAWVLSLCMSLPLLVFADV
QEGGTCNASWPEPVGLWGAVFIIYTAVLGFFAPLLVICLCYLLIVVKVRAAGVRVGCVRR
RSERKVTRMVLVVVLVFAGCWLPFFTVNIVNLAVALPQEPASAGLYFFVVILSYANSCAN
PVLYGFLSDNFRQSFQKVLCLRKGSGAKDADATEPRPDRIRQQQEATPPAHRAAANGLMQ
TSKL

>hsa:6915
MWPNGSSLGPCFRPTNITLEERRLIASPWFAASFCVVGLASNLLALSVLAGARQGGSHTR
SSFLTFLCGLVLTDFLGLLVTGTIVVSQHAALFEWHAVDPGCRLCRFMGVVMIFFGLSPL
LLGAAMASERYLGITRPFSRPAVASQRRAWATVGLVWAAALALGLLPLLGVGRYTVQYPG
SWCFLTLGAESGDVAFGLLFSMLGGLSVGLSFLLNTVSVATLCHVYHGQEAAQQRPRDSE
VEMMAQLLGIMVVASVCWLPLLVFIAQTVLRNPPAMSPAGQLSRTTEKELLIYLRVATWN
QILDPWVYILFRRAVLRRLQPRLSTRPRSLSLQPQLTQRSGLQ

>hsa:7201
MENETVSELNQTQLQPRAVVALEYQVVTILLVLIICGLGIVGNIMVVLVVMRTKHMRTPT
NCYLVSLAVADLMVLVAAGLPNITDSIYGSWVYGYVGCLCITYLQYLGINASSCSITAFT
IERYIAICHPIKAQFLCTFSRAKKIIIFVWAFTSLYCMLWFFLLDLNISTYKDAIVISCG
YKISRNYYSPIYLMDFGVFYVVPMILATVLYGFIARILFLNPIPSDPKENSKTWKNDSTH
QNTNLNVNTSNRCFNSTVSSRKQVTKMLAVVVILFALLWMPYRTLVVVNSFLSSPFQENW
FLLFCRICIYLNSAINPVIYNLMSQKFRAAFRKLCNCKQKPTEKPANYSVALNYSVIKES
DHFSTELDDITVTDTYLSATKVSFDDTCLASEVSFSQS

>hsa:8843
MNRHHLQDHFLEIDKKNCCVFRDDFIAKVLPPVLGLEFIFGLLGNGLALWIFCFHLKSWK
SSRIFLFNLAVADFLLIICLPFVMDYYVRRSDWKFGDIPCRLVLFMFAMNRQGSIIFLTV
VAVDRYFRVVHPHHALNKISNWTAAIISCLLWGITVGLTVHLLKKKLLIQNGTANVCISF
SICHTFRWHEAMFLLEFFLPLGIILFCSARIIWSLRQRQMDRHAKIKRAITFIMVVAIVF
VICFLPSVVVRIHIFWLLHTSGTQNCEVYRSVDLAFFITLSFTYMNSMLDPVVYYFSSPS
FPNFFSTLINRCLQRKITGEPDNNRSTSVELTGDPNKTRGAPEALIANSGEPWSPSYLGP
TSNNHSKKGHCHQEPASLEKQLGCCIE

>hsa:9052
MATTVPDGCRNGLKSKYYRLCDKAEAWGIVLETVATAGVVTSVAFMLTLPILVCKVQDSN
RRKMLPTQFLFLLGVLGIFGLTFAFIIGLDGSTGPTRFFLFGILFSICFSCLLAHAVSLT
KLVRGRKPLSLLVILGLAVGFSLVQDVIAIEYIVLTMNRTNVNVFSELSAPRRNEDFVLL
LTYVLFLMALTFLMSSFTFCGSFTGWKRHGAHIYLTMLLSIAIWVAWITLLMLPDFDRRW
DDTILSSALAANGWVFLLAYVSPEFWLLTKQRNPMDYPVEDAFCKPQLVKKSYGVENRAY
SQEEITQGFEETGDTLYAPYSTHFQLQNQPPQKEFSIPRAHAWPSPYKDYEVKKEGS

>hsa:9283
MRWLWPLAVSLAVILAVGLSRVSGGAPLHLGRHRAETQEQQSRSKRGTEDEEAKGVQQYV
PEEWAEYPRPIHPAGLQPTKPLVATSPNPGKDGGTPDSGQELRGNLTGAPGQRLQIQNPL

YPVTESSYSAYAIMLLALVVFAVGIVGNLSVMCIVWHSYYLKSAWNSILASLALWDFLVL
FFCLPIVIFNEITKQRLLGDVSCRAVPFMEVSSLGVTTFSLCALGIDRFHVATSTLPKVR
PIERCQSILAKLAVIWVGSMTLAVPELLLWQLAQEPAPTMGTLDSCIMKPSASLPESLYS
LVMTYQNARMWWYFGCYFCLPILFTVTCQLVTWRVRGPPGRKSECRASKHEQCESQLNST
VVGLTVVYAFCTLPENVCNIVVAYLSTELTRQTLDLLGLINQFSTFFKGAITPVLLLCIC
RPLGQAFLDCCCCCCCEECGGASEASAANGSDNKLKTEVSSSIYFHKPRESPPLLPLGTP
C

>hsa:9934
MINSTSTQPPDESCSQNLLITQQIIPVLYCMVFIAGILLNGVSGWIFFYVPSSKSFIIYL
KNIVIADFVMSLTFPFKILGDSGLGPWQLNVFVCRVSAVLFYVNMYVSIVFFGLISFDRY
YKIVKPLWTSFIQSVSYSKLLSVIVWMLMLLLAVPNIILTNQSVREVTQIKCIELKSELG
RKWHKASNYIFVAIFWIVFLLLIVFYTAITKKIFKSHLKSSRNSTSVKKKSSRNIFSIVF
VFFVCFVPYHIARIPYTKSQTEAHYSCQSKEILRYMKEFTLLLSAANVCLDPIIYFFLCQ
PFREILCKKLHIPLKAQNDLDISRIKRGNTTLESTDTL
